# Sociodemographic and clinical predictors of malignancy in thyroid nodules: a systematic review and meta-analysis

**DOI:** 10.3389/fendo.2026.1885823

**Published:** 2026-08-26

**Authors:** Vanessa Neto, Catarina Leitão, Cláudia Martins, Margarida Fardilha, Maria Teresa Herdeiro, Alexandra Nunes

**Affiliations:** iBiMED - Institute of Biomedicine, Department of Medical Sciences, University of Aveiro, Aveiro, Portugal

**Keywords:** meta-analysis, risk factors, systematic review, thyroid cancer, thyroid nodules

## Abstract

**Background:**

Thyroid nodules (TNs) are prevalent, particularly in women and older adults, yet only a minority are malignant. Clinical decisions on biopsy and follow-up are complicated by selective use of fine-needle aspiration cytology, indeterminate results, and efforts to limit unnecessary procedures. We systematically identified and synthesized sociodemographic, laboratory, and ultrasonographic (US) factors associated with malignancy in TNs.

**Methods:**

PubMed, Scopus, Web of Science, and EMBASE were searched (PROSPERO CRD42024557085). Two reviewers independently screened studies, extracted data, and assessed risk of bias (Joanna Briggs Institute tools). Random-effects meta-analyses were performed for predictors reported in ≥5 studies, with heterogeneity quantified using I^2^. Pooled odds ratios (ORs) with 95% confidence intervals (CIs) were calculated. Malignancy was defined according to each study’s reference standard (histology and/or cytology). Sensitivity analyses assessed robustness across diagnostic method and key study-level characteristics (e.g. geographic region and sample size).

**Results:**

Of 35,359 records, 194 studies met the inclusion criteria. Of these, 47 with poolable crude ORs (predictors reported in ≥5 studies) contributed to meta-analysis, and the remainder to qualitative synthesis. Male sex, family history of thyroid cancer, higher thyroid-stimulating hormone levels, and several US features (including hypoechogenicity, solid composition, calcifications/microcalcifications, taller-than-wide shape, and irregular margins) were associated with higher odds of malignancy, whereas increasing age and larger nodule size showed inverse associations, though with substantial between-study heterogeneity.

**Conclusions:**

Malignancy in TNs reflects both patient-related and nodule-specific characteristics. Integrating these factors may support pre-biopsy risk assessment and more consistent biopsy and follow-up decisions.

**Systematic Review Registration:**

https://www.crd.york.ac.uk/PROSPERO/, identifier CRD42024557085.

## 1 Introduction

Thyroid nodules (TNs) are a common clinical finding, with reported prevalence varying substantially according to the detection method. While palpation identifies TNs in approximately 5-7% of the general adult population, high-resolution ultrasound can detect nodules in up to 68% of individuals, particularly among older adults and women (1, 2). Despite their high frequency, fewer than 5% of TNs are malignant (3). Therefore, the main clinical challenge is to accurately distinguish malignant from benign nodules to guide appropriate management and minimize unnecessary procedures (2).

Current diagnostic strategies rely primarily on fine-needle aspiration cytology (FNAC), typically performed under ultrasound guidance (4). The Bethesda System for Reporting Thyroid Cytopathology (TBSRTC) standardizes cytological classification, and benign (Bethesda II) and malignant (Bethesda VI) results are generally reliable for clinical decision-making (5). However, important limitations persist. A considerable proportion of TNs fall into indeterminate cytology categories (Bethesda III-V), often leading to diagnostic surgery despite ultimately benign histology (6). Moreover, as current guidelines recommend FNAC only for a subset of nodules based on size and ultrasound risk patterns, many are not evaluated cytologically (7). Pre-biopsy assessment of TNs with suspicious features remains guided by the 2015 American Thyroid Association (ATA) guidelines and parallel systems such as ACR-TIRADS and EU-TIRADS (7–9). The 2025 ATA update focuses exclusively on the management of differentiated thyroid carcinomas (DTC) (10), so the 2015 recommendations continue to serve as the reference for nodule risk stratification. In this context, clarifying which pre-biopsy factors are associated with malignancy may help refine selection for FNAC and support more individualized risk assessment when cytology is available.

Most thyroid cancers (TC) are DTC, primarily papillary (PTC) and follicular (FTC) subtypes (11, 12). Despite their generally favourable prognosis, the incidence of TC has increased in recent decades, raising concerns about overdiagnosis and overtreatment (12, 13). This trend has reinforced the need for robust risk stratification approaches that identify nodules more likely to represent clinically meaningful malignancy (14). In addition to established non-modifiable factors such as age, male sex, and family history, a variety of patient-related, clinical/laboratory variables, including thyroid-stimulating hormone (TSH) levels and obesity, as well as US characteristics have also been investigated as potential predictors of malignancy (15, 16), but results remain dispersed and inconsistently summarized across studies.

Given these diagnostic limitations and uncertainties regarding the contribution of patient-related, and clinical characteristics, consolidating existing evidence is warranted. This systematic review and meta-analysis aims to synthesize the available literature on sociodemographic factors, and clinical variables associated with malignancy in TNs. By identifying the most consistently reported predictors and quantifying pooled associations where feasible, this work seeks to support pre-biopsy risk assessment, inform biopsy and follow-up decisions, and highlight evidence gaps to guide future prospective studies.

## 2 Methods

### 2.1 Protocol and registration

The protocol for this systematic review and meta-analysis was registered in the International Prospective Register of Systematic Reviews (PROSPERO) (CRD42024557085) (17). The review was conducted and reported in accordance with the Preferred Reporting Items for Systematic Review and Meta-Analysis (PRISMA) guidelines (18).

### 2.2 Search strategy

A systematic search was conducted in four electronic databases - MEDLINE via PubMed, Scopus, Web of Science, and EMBASE - to identify studies published between January 1^st^, 2014 and May 31^st^, 2024. The search strategy primarily aimed to identify relevant studies evaluating predictors/risk factors for malignancy in TNs, including sociodemographic and clinical domains (laboratory and US features). The keywords used to search the aforementioned databases were:

(determinant* OR factor* OR demographic) AND thyroid AND (nodule* OR cancer OR carcinoma OR neoplas* OR tumor OR adenoma) AND (progression OR incidence OR risk).

Full search expression for each database is available in Text S1 (**Supplementary Material**).

### 2.3 Study selection and eligibility criteria

Study selection was conducted independently by two reviewers (VN and CL) using Rayyan, a web-based tool specifically designed to efficiently organize and manage systematic reviews (19), in two stages: i) title/abstract screening and ii) full-text review of potentially eligible records. In cases of disagreement, a third researcher (CM) was consulted to reach consensus.

Studies were included in this review if they: i) were written in English, Portuguese or Spanish; ii) had an observational study design; iii) assessed the association between any sociodemographic or clinical feature and malignancy in individuals with TNs, without confirmed TC at baseline. Studies enrolling adults, with no restriction on whether the study population consisted exclusively of adults or included both adults and pediatric participants (mixed-age populations), were eligible. Studies restricted exclusively to children and adolescents were excluded. Malignancy was defined according to each study’s reference standard (histology and/or cytology), and all histological subtypes of TC were eligible for inclusion.

Studies not conducted in humans (e.g., animal or *in vitro* studies), review articles, meta-analysis, experimental studies, qualitative studies, ecological studies, news, magazines, research protocols, reports, thesis, dissertations, abstracts, posters, communications, unpublished work, letters to the editor, commentaries, editorials, books, book chapters, guidelines, statements, position papers, and studies that were not within the scope of our study were excluded. Studies restricted to populations exposed to radiation from nuclear disasters (e.g., Chernobyl, Fukushima) or studies assessing the relationship between unrelated comorbidities and TC in the general population without confirmed TNs were also excluded.

### 2.4 Risk of bias assessment

Risk of bias was assessed using the appropriate Joanna Briggs Institute (JBI) critical appraisal checklist according to each study design (20). Similar to the screening process, two researchers (VN and CM) independently completed the assessments. Disagreements were resolved through discussion to reach consensus, and, when necessary, a third researcher (CL) was consulted to provide the final decision.

### 2.5 Data extraction

Data was extracted independently by two researchers (VN and CL), who retrieved information on authors, publication year, location, study design, sample size (number of TNs and of malignant cases), participants’ characteristics (age and sex), histological subtype of malignant cases (when reported), diagnostic method(s), predictors assessed (sociodemographic and/or clinical), and reported effect estimates [odds ratios (ORs), hazard ratios (HRs), and relative risks (RRs)].

For this systematic review, variables such as age, sex, ethnicity, place of residence, and other related population-level characteristics were considered sociodemographic factors. Clinical variables included anthropometric measurements, thyroid-related clinical/laboratory data (including hormonal and antibody profiles), family history of TC, compressive symptoms, and US nodule features. Although thyroglobulin (Tg) is not a thyroid hormone, it was grouped with thyroid hormones for synthesis purposes due to its clinical relevance in thyroid disease.

### 2.6 Statistical analysis

All statistical analyses were conducted using RStudio software (v.4.5.1) using the metafor package (21), applying random-effects models to account for between-study variability. Heterogeneity was assessed using Cochran’s Q statistic and the inconsistency index (I^2^) (22), interpreted as low (<30%), moderate (30% - <50%), substantial (50% - <75%) or high (≥75%) (23), and visually inspected with forest plots. Meta-analyses were conducted only for predictors reported in at least five studies. Because adjustment strategies varied widely across studies, pooled analyses were restricted to crude ORs. When studies reported only adjusted estimates, or only RR/HR without an OR, these effect sizes were extracted for qualitative synthesis but were not included in quantitative pooling. For continuous predictors, such as age, nodule size, and TSH, which were inconsistently reported as continuous or categorical variables, meta-analysis was restricted to studies reporting effects on a continuous scale to avoid heterogeneity introduced by differing categorical thresholds. Where measurement units differed across studies, effect estimates were rescaled to a common unit prior to pooling (e.g., nodule size converted to OR per 1 cm increase by rescaling the log(OR) and its standard error). Potential publication bias was explored using funnel plots when sufficient studies were available for a given predictor (**Supplementary Figure 1**). When substantial heterogeneity was observed, potential moderators were explored using meta-regression. Where clinically relevant categorical moderators were identified, subgroup analyses were performed. Leave-one-out analyses were conducted to evaluate the influence of individual studies on pooled estimates, with results provided in the **Supplementary Material** on the log-odds scale. Pooled effects are reported as ORs with 95% confidence intervals (CIs), and meta-regression results are presented as regression coefficients (β) on the log-odds scale.

## 3 Results

### 3.1 Study selection and quality assessment

**Figure 1:**
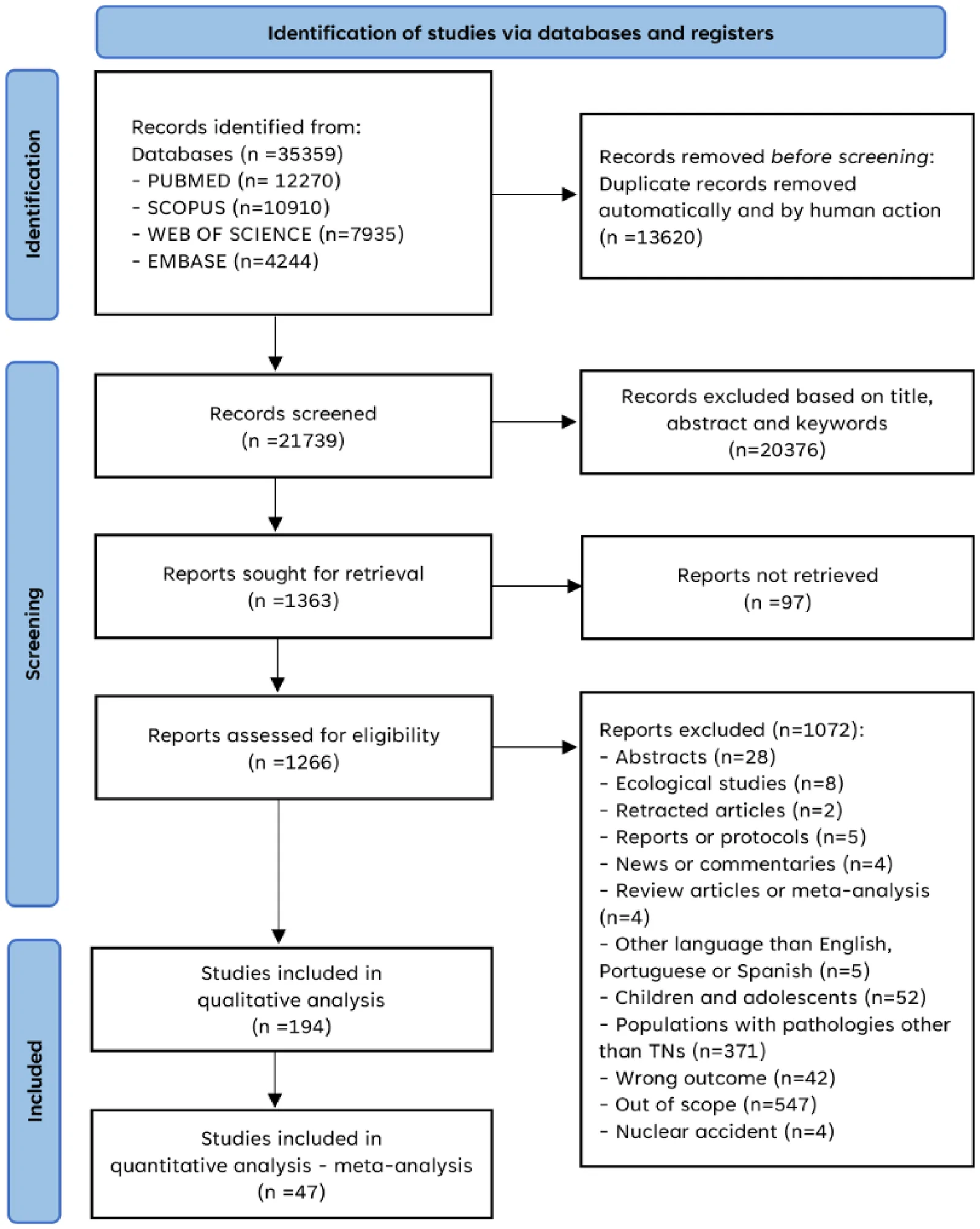
Literature search flowchart.

**Figure 1** presents the literature search flowchart. The systematic database search retrieved 35,359 records. After removal of 13,620 duplicates, title and abstract were screened for 21,739 records with 20,376 excluded. Full texts were assessed for 1,266 papers and a total of 194 studies met eligibility criteria and were included.

Risk of bias was assessed using the JBI checklist by study design (**Supplementary Material**). The most frequent limitations across designs were related to the identification and handling of confounding factors. In cohort studies, additional concerns included ensuring participants were free of the outcome at baseline and incomplete or unclear follow-up. In case-control studies, limitations mainly involved group comparability and matching.

### 3.2 Study characteristics

Baseline characteristics of the 194 included studies are summarized in **Table 1**.

**Table 1:** Characteristics of the included studies.

Author and year	Location	Study design	No. of nodules (no. of malignant cases)	Age (years)	Sex (male/female)	Malignant subtype	Diagnostic method	Risk factors (n)
Ageeli, RS - 2022 (24)	Saudi Arabia	Cohort (retrospective)	366 (52)	≥18	45/321	MN	FNAC	Sociodemographic (2) Clinical (1)
Ajarma, KY - 2020 (25)	Jordan	Cohort (retrospective)	567 (192)	44.3 ± 14.5	126/441	MN	Histopathology	Sociodemographic (2) Clinical (2)
Al Dawish, MA - 2018 (15)	Saudi Arabia	Cohort (retrospective)	315 (95)	46.3 ± 15.1	62/253	MN	Histopathology	Clinical (2)
Al Dawish, MA - 2020 (26)	Saudi Arabia	Cohort (retrospective)	167 (46)	15-90	49/118	MN	Histopathology	Clinical (2)
Alaraifi, AK - 2023 (16)	Saudi Arabia	Cohort (retrospective)	421 (284)	45.24 ± 13.4 (benign) 46.27 ± 14.64 (malignant)	83/338	MN	Histopathology	Sociodemographic (2) Clinical (3)
Al-Bader, A - 2015 (27)	Canada	Cohort (retrospective)	267 (163)	50 ± NR	55/212	WDTC	Histopathology	Clinical (1)
Alqahtani, SM - 2024 (28)	Saudi Arabia	Cohort (retrospective)	102 (48)	40.1 ± 10.7	23/79	MN	Histopathology	Sociodemographic (2) Clinical (10)
Angell, TE - 2017 (29)	USA	Cohort (prospective)	1,489 (126)	52.2 ± 13.6 (benign) 48.6 ± 14.5 (malignant)	155/1,334	MN	FNAC and Histopathology	Clinical (1)
Angell, TE - 2019 (30)	USA	Cohort (prospective)	20,001 (1,974)	18-95	1,595/8,372	MN	FNAC and Histopathology	Sociodemographic (2) Clinical (3)
Apostolou, K - 2021 (31)	Serbia	Cohort (retrospective)	3,233 (1,026)	56.8 ± 12.5 (benign) 55.4 ± 12.7 (malignant)	445/2,788	MN, PTC, PTMC, HCC	Histopathology	Sociodemographic (2) Clinical (1)
Arduc, A - 2015 (32)	Turkey	Cross-sectional	224 (82)	55.75 ± 12.2 (benign) 48.44 ± 10.6 (malignant)	0/224	MN	Histopathology	Sociodemographic (1) Clinical (8)
Aydin, Y - 2014 (33)	Turkey	Cohort (retrospective)	103 (21)	48.6 ± 11.1	22/81	MN	Histopathology	Clinical (1)
Babayid, Y - 2024 (34)	Turkey	Cohort (retrospective)	6,395 (1,188)	55.3 ± 13.0	1,886/7,618	MN	Histopathology	Sociodemographic (2) Clinical (8)
Baser, H - 2016 (35)	Turkey	Cohort (retrospective)	3,206 (460)	49.0 ± NR (benign) 49.0 ± NR (malignant)	284/1,149	MN	Histopathology	Clinical (3)
Batawil, N - 2014 (36)	Saudi Arabia	Cohort (retrospective)	78 (28)	38 ± 12 (benign) 45 ± 16 (malignant)	8/70	MN	Histopathology	Sociodemographic (2) Clinical (5)
Behbahaninia, M - 2022 (37)	Iran	Cross-sectional	984 (144)	45.58 ± 13.21	170/637	MN	FNAC	Clinical (6)
Boonrod, A - 2021 (38)	USA	Cohort (retrospective)	115 (57)	55.2 ± 15 (benign) 62.6 ± 16 (malignant)	45/70	MN	Histopathology	Sociodemographic (2) Clinical (3)
Brophy, C - 2016 (39)	Ireland	Cohort (retrospective)	858 (45)			MN	FNAC and Histopathology	Clinical (2)
Bukasa Kakamba, J-2021 (40)	DRC	Cross-sectional	594 (119)	43.9 ± 13.6 (benign) 45.3 ± 16.7 (malignant)	94/497	MN	Histopathology	Sociodemographic (1) Clinical (5)
Campbell, MJ - 2015 (41)	USA	Cohort (retrospective)	538 (44)	54.1 ± NR (benign) 56.3 ± NR (malignant)	92/446	MN	Histopathology	Sociodemographic (2) Clinical (3)
Canete, EJ - 2014 (42)	Philippines	Cohort (retrospective)	837 (420)	37.59 ± 10.58 (benign) 38.68 ± 12.89 (malignant)	131/706	MN	Histopathology	Sociodemographic (1) Clinical (8)
Cao, SL - 2024 (43)	China	Cross-sectional	280 (163)	49 ± NR (benign) 40 ± NR (malignant)	82/198	MN	FNAC	Sociodemographic (2) Clinical (5)
Cappelli, C - 2019 (44)	Italy	Cross-sectional	242 (128)	54.7 ± 11.4 (benign) 54.0 ± 12.5 (malignant)	50/186	MN	FNAC	Sociodemographic (2) Clinical (4)
Castagna, MG-2014 (45)	Italy	Cohort (retrospective)	3,990 (121)	58.3 ± 14.1	475/2,029	MN	FNAC and Histopathology	Clinical (2)
Cavallo, A – 2017 (46)	USA	Cohort (retrospective)	1,003 (258)	52.8 ± NR		MN	FNAC and Histopathology	Clinical (1)
Chen, WH – 2022 (47)	Taiwan	Cohort (retrospective)	151 (79)	57.2 ± 12.5 (benign) 52.6 ± 12.5 (malignant)	25/126	PTMC	Histopathology	Sociodemographic (1) Clinical (3)
Chen, Z – 2022 (48)	China	Cohort (retrospective)	319 (67)	43.4 ± 13.8 (benign) 45.1 ± 16.0 (malignant)	104/215	FTC	Histopathology	Sociodemographic (2) Clinical (3)
Chen, X – 2023 (49)	China	Cross-sectional	267 (191)	42.08 ± 9.36 (benign) 39.60 ± 11.39 (malignant)	77/190	PTC	FNAC and Histopathology	Clinical (2)
Chiefari, E – 2024 (50)	Italy	Cohort (retrospective)	648 (81)	52.9 ± 11.0	118/531	PTMC	Histopathology	Sociodemographic (1)
Chiofalo, MG – 2018 (51)	Italy	Cross-sectional	902 (435)	18-87	213/689	MN	Histopathology	Sociodemographic (2) Clinical (2)
Cho, YY - 2021 (52)	Korea	Cohort (retrospective)	357 (170)		82/275	DTC	Histopathology	Sociodemographic (2) Clinical (4)
Choi, JS – 2015 (53)	Korea	Cohort (retrospective)	1,269 (342)	50.9 ± 11.6 (benign) 47.6 ± 11.7 (malignant)	176/1,037	MN	FNAC and Histopathology	Sociodemographic (2) Clinical (2)
Cozzolino, A – 2020 (54)	Italy	Cohort (prospective)	65 (25)		15/50	MN	Histopathology	Sociodemographic (2) Clinical (9)
Crudele, L – 2023 (55)	Italy	Cross-sectional	142 (21)	56.1 ± 12.8 (benign) 54.1 ± 12.7 (malignant)	47/95	MN	FNAC	Clinical (2)
Delman, AM – 2023 (56)	USA	Cohort (retrospective)	10,262 (7,672)	52 ± NR	3,235/9,886	MN	Histopathology	Sociodemographic (2)
Deng, XH – 2017 (57)	China	Cohort (retrospective)	1,000 (98)	52.8 ± 12.8 (benign) 49.0 ± 14.0 (malignant)	222/778	MN	FNAC and Histopathology	Sociodemographic (1) Clinical (4)
Deng, Y – 2022 (58)	China	Cohort (retrospective)	514 (140)	≥18	102/412	MN	FNAC and Histopathology	Sociodemographic (2) Clinical (3)
Ding, J – 2019 (59)	China	Cohort (retrospective)	153 (69)	47.0 ± 13.2	35/118	MN	Histopathology	Sociodemographic (2) Clinical (3)
Dobruch-Sobczak, K – 2019 (60)	Poland	Cohort (retrospective)	842 (229)	14-86		MN	Histopathology	Clinical (5)
Dong, S – 2021 (61)	China	Cohort (retrospective)	367 (112)	49.0 ± 13.5	90/277	PTC	Histopathology	Sociodemographic (1) Clinical (5)
Du, J – 2023 (62)	China	Cohort (retrospective)	464 (359)	53.73 ± 13.14 (benign) 46.19 ± 12.00 (malignant)	124/340	DTC	Histopathology	Sociodemographic (2) Clinical (7)
Ebrahim, H – 2023 (63)	Ethiopia	Cross-sectional	424 (70)	37.74 ± 17.78	155/269	MN	FNAC	Sociodemographic (2) Clinical (2)
Egset, AV – 2017 (64)	Denmark	Cohort (prospective)	483 (108)	53.0 ± NR	109/374	MN	Histopathology	Clinical (1)
Eisa, N – 2018 (65)	USA	Cohort (retrospective)	97 (30)	54.8 ± NR (benign) 53.0 ± NR (malignant)		MN	Histopathology	Sociodemographic (2) Clinical (2)
Eissa, MS – 2020 (66)	Egypt	Cross-sectional	203 (19)	42.7 ± 12.3 (benign) 43.3 ± 11.9 (malignant)	9/194	MN	Histopathology	Clinical (2)
El Sayed Ibrahim, MM – 2019 (67)	Egypt	Cohort (retrospective)	83 (33)	36.96 ± 11.1	19/64	MN	Histopathology	Sociodemographic (2) Clinical (4)
Elbalka, SS – 2021 (68)	Egypt	Cohort (retrospective)	1,098 (371)	42.9 ± 12.4 (benign) 42.5 ± 15.6 (malignant)	193/905	MN	Histopathology	Sociodemographic (1) Clinical (1)
Espinosa De Ycaza, AE – 2016 (69)	USA	Cohort (retrospective)	495 (15)	61 ± NR (benign) 49 ± NR (malignant)	131/341	MN	FNAC and Histopathology	Sociodemographic (2) Clinical (7)
Fernández-Trujillo, C – 2020 (70)	Spain	Cohort (retrospective)	127 (49)	50.1 ± 13.5 (benign) 57.0 ± 13.7 (malignant)	16/111	MN	Histopathology	Sociodemographic (1) Clinical (1)
Fighera, TM – 2015 (71)	Brasil	Cohort (retrospective)	622 (110)	50.0 ± 14.0 (benign) 45.8 ± 13.0 (malignant)	55/567	MN	FNAC and Histopathology	Sociodemographic (2) Clinical (3)
Gallegos-Vargas, JA – 2016 (72)	Mexico	Case-control	64 (32)	53.66 ± 13.62 (benign) 54.81 ± 14.77 (malignant)	13/51	MN	Histopathology	Sociodemographic (2)
Garino, F – 2015 (73)	Italy	Cohort (prospective)	108 (33)	50 ± NR	27/81	MN	Histopathology	Clinical (4)
Gezer, E – 2022 (74)	Turkey	Cohort (retrospective)	387 (157)	48.8 ± 11.4 (benign) 46.9 ± 12.3 (malignant)	53/334	DTC	Histopathology	Sociodemographic (2) Clinical (2)
Grani, G – 2015 (75)	Italy	Cohort (prospective)	1,989 (100)	55.89 ± 13.06 (benign) 45.07 ± 16.17 (malignant)	285/1,704	MN	FNAC and Histopathology	Sociodemographic (2) Clinical (6)
Grani, G – 2023 (76)	Italy	Cohort (prospective)	903 (76)	46-66	228/624	MN	FNAC and Histopathology	Clinical (5)
Gu, WJ – 2015 (77)	China	Cohort (retrospective)	2,414 (688)	53.1 ± 13.5 (benign) 52.2 ± 12.9 (malignant)	753/1,661	PTC	Histopathology	Clinical (7)
Guarnotta, V – 2023 (78)	Italy	Cohort (prospective)	103 (61)	56.1 ± 14.5 (benign) 52.9 ± 14.1 (malignant)	27/76	MN	Histopathology	Clinical (7)
Gweon, HM – 2017 (79)	Korea	Cohort (retrospective)	1,866 (1045)	49 (median)	366/1,500	MN	FNAC and Histopathology	Sociodemographic (1) Clinical (3)
Hacim, NA – 2021 (80)	Turkey	Cohort (retrospective)	159 (91)	45.9 ± 12.5 (benign) 49.7 ± 13.8 (malignant)	45/114	MN	Histopathology	Clinical (7)
Handelsman, RS – 2019 - (81)	USA	Cohort (retrospective)	991 (517)	48 ± 14	150/841	PTC	Histopathology	Sociodemographic (1)
Hong, IK – 2016 (82)	Korea	Cohort (retrospective)	129 (31)	52.54 ± 11.84 (benign) 49.32 ± 11.66 (malignant)	21/108	MN	FNAC and Histopathology	Clinical (1)
Hong, SH – 2018 (83)	Korea	Cohort (retrospective)	96 (70)	51 ± 11 (benign) 52 ± 11 (malignant)	9/87	MN	FNAC and Histopathology	Clinical (5)
Hou, CJ – 2018 (84)	China	Cohort (retrospective)	246 (123)	26-80	0/246	PTMC	Histopathology	Clinical (4)
Huan, Q – 2014 (85)	China	Cohort (retrospective)	6,304 (2,156)	49.31 ± 12.41 (benign) 44.59 ± 12.22 (malignant)	1,494/4,810	MN	FNAC and Histopathology	Sociodemographic (2) Clinical (8)
Huang, J – 2020 (86)	China	Cohort (retrospective)	574 (257)	47.19 ± 12.03 (benign) 40.38 ± 10.40 (malignant)	150/424	PTC	Histopathology	Sociodemographic (3) Clinical (2)
Huang, J – 2021 (87)	China	Cohort (retrospective)	272 (110)	43.8 ± 11.2	68/204	PTC	Histopathology	Clinical (6)
Hwang, SH – 2016 (88)	Korea	Cohort (retrospective)	1,310 (579)	51.2 ± 12.0 (benign) 47.6 ± 12.8 (malignant)	301/1,009	MN	FNAC and Histopathology	Sociodemographic (2) Clinical (3)
Ito, Y – 2016 (89)	Japan	Cohort (retrospective)	330 (69)	16-81	76/254	OFTC	Histopathology	Sociodemographic (2) Clinical (2)
Jabar, ASS – 2019 (90)	India	Cross-sectional	127 (23)	46.6 ± 13.3 (benign) 47.3 ± 12.6 (malignant)	17/110	MN	FNAC and Histopathology	Clinical (5)
Jasim, S – 2020 (91)	USA	Cohort (retrospective)	3,241 (335)	18-97	667/2,635	MN	FNAC and Histopathology	Sociodemographic (2) Clinical (3)
Jia, X – 2020 (92)	China	Cohort (retrospective)	4,246 (2,889)	50.35 ± 11.66 (benign) 44.36 ± 10.70 (malignant)	1,161/2,885	PTC	Histopathology	Sociodemographic (2) Clinical (3)
Kaliszewski, K – 2018 (93)	Poland	Cohort (retrospective)	127 (13)	49.5 ± 15.9 (benign) 52.5 ± 14.1 (malignant)	22/105	MN	Histopathology	Sociodemographic (2) Clinical (6)
Kaliszewski, K – 2021 (94)	Poland	Cohort (retrospective)	161 (19)	51.26 ± 15.41	58/284	MN	Histopathology	Sociodemographic (2) Clinical (5)
Kaliszewski, K – 2021 (95)	Poland	Cohort (retrospective)	161 (19)	50.6 ± 16.12	31/130	MN	Histopathology	Sociodemographic (1) Clinical (6)
Kaliszewski, K – 2022 (96)	Poland	Cohort (retrospective)	342 (47)	52.1 ± 15.5 (benign) 46.0 ± 14.0 (malignant)	58/284	MN	Histopathology	Sociodemographic (2) Clinical (6)
Karabacak, U – 2022 (97)	Turkey	Cohort (retrospective)	156 (44)	21-87	39/115	MN	Histopathology	Clinical (2)
Khodabandelu, S – 2022 (98)	Iran	Cohort (retrospective)	431 (61)	48.15 ± 12.00 (benign) 20.65 ± 14.42 (malignant)	54/377	MN	FNAC	Sociodemographic (2) Clinical (8)
Kim, GR – 2016 (99)	Korea	Cohort (retrospective)	2,005 (1,237)	44.0 ± 11.7	414/1,591	PTC	FNAC and Histopathology	Sociodemographic (2) Clinical (5)
Kim, HJ – 2017 (100)	Korea	Cohort (retrospective)	198 (32)	51 ± 13 (benign) 48 ± 16 (malignant)	39/159	FTC	FNAC and Histopathology	Sociodemographic (2) Clinical (2)
Kim, HJ – 2017 (101)	Korea	Cross-sectional	1,170 (206)	18-75	203/967	MN	FNAC and Histopathology	Sociodemographic (2) Clinical (3)
Kim, K – 2021 (102)	Korea	Cohort (retrospective)	416 (209)	48.3 ± 13.7 (benign) 45.8 ± 13.6 (malignant)	99/317	FTC	Histopathology	Sociodemographic (2)
Kişioğlu, SV – 2022 (103)	Turkey	Cohort (retrospective)	255 (99)	49.86 ± 12.52	62/149	MN	FNAC and Histopathology	Clinical (4)
Kong, J – 2017 (104)	China	Cohort (retrospective)	113 (79)	52.71 ± 14.49 (benign) 39.97 ± 9.04 (malignant)	48/44	MN	Histopathology	Clinical (3)
Kornelius, E – 2020 (105)	Taiwan	Cohort (retrospective)	132 (19)	50.8 ± 13.6	35/97	MN	Histopathology	Sociodemographic (2) Clinical (2)
Kotecka-Blicharz, A – 2022 (106)	Poland	Cohort (retrospective)	175 (58)	52 [41.5, 63.0]	25/150	MN	Histopathology	Clinical (4)
Krátký, J – 2018 - (107)	Czech Republic	Cross-sectional	273 (91)	57 ± NR	60/213	MN	FNAC and Histopathology	Clinical (3)
Kuo, TC – 2020 (108)	Taiwan	Cohort (retrospective)	188 (49)	48 ± 15.6 (benign) 51 ± 18.5 (malignant)	58/130	FTC	Histopathology	Clinical (2)
Kuru, B – 2016 (109)	Turkey	Cohort (retrospective)	153 (35)		32/121	MN	Histopathology	Clinical (5)
Kuru, B – 2018 (110)	Turkey	Cohort (retrospective)	139 (51)		28/111	MN	Histopathology	Clinical (5)
Lee, KH – 2014 (111)	Korea	Cohort (retrospective)	152 (64)	47.9 ± NR (benign) 45.4 ± NR (malignant)	35/117	MN	FNAC and Histopathology	Sociodemographic (1) Clinical (5)
Li, T – 2015 (112)	China	Cohort (retrospective)	13,980 (2,966)	48.56 ± 12.35 (benign) 47.28 ± 12.41 (malignant)	3,170/10,810	MN	Histopathology	Sociodemographic (1) Clinical (7)
Li, W – 2021 (113)	China	Case-control	81 (28)	48.6 ± 12.2 (benign) 45.1 ± 17.3 (malignant)	17/64	FTC	Histopathology	Clinical (4)
Li, W – 2024 (114)	China	Cross-sectional	248 (125)	40 [33, 52]	46/131	MN	Histopathology	Clinical (3)
Liu, Y – 2016 (115)	China	Cross-sectional	102 (52)	19-80	29/73	PTC	Histopathology	Clinical (2)
Liu, J – 2018 (116)	China	Cross-sectional	2,984 (524)	48.5 ± 11.5 (benign) 43.5 ± 11.6 (malignant)	541/2,443	MN	Histopathology	Sociodemographic (1) Clinical (9)
Lourdes Ng, J – 2023 (117)	Philippines	Cohort (retrospective)	59 (38)	46.57 ± 10.14 (benign) 46.68 ± 12.34 (malignant)	8/51	MN	FNAC and Histopathology	Sociodemographic (2) Clinical (6)
Lynch, CA – 2022 (118)	USA	Cohort (retrospective)	256 (63)	53.3 ± 14.0	56/200	MN	Histopathology	Sociodemographic (3) Clinical (2)
Macias, CA – 2015 (119)	USA	Cohort (retrospective)	151 (35)	53.7 ± 13.6 (benign) 47.2 ± 16.3 (malignant)	40/111	MN	Histopathology	Sociodemographic (1) Clinical (2)
Maeda, H – 2016 (120)	Japan	Cohort (retrospective)	125 (64)	53.0 ± 14.3 (benign) 54.2 ± 17.3 (malignant)	16/108	MN	Histopathology	Clinical (1)
Mazzone, M – 2021 (121)	Italy	Cross-sectional	246 (123)	57.0 [47.0, 65.5] (benign) 52 [44, 63] (malignant)	44/202	MN	Histopathology	Sociodemographic (3) Clinical (2)
Melak, T – 2014 (122)	Ethiopia	Cross-sectional	846 (62)	2-80	185/661	MN	FNAC and Histopathology	Sociodemographic (2) Clinical (2)
Mileva, M – 2018 (123)	North Macedonia	Cohort (retrospective)	112 (35)	54.9 ± 11.7 (benign) 48.0 ± 14.2 (malignant)	18/87	MN	FNAC and Histopathology	Sociodemographic (1) Clinical (4)
Mohamed, TZ – 2022 (124)	Egypt	Cohort (retrospective)	98 (21)	47.13 ± 11.94 (benign) 48.67 ± 10.66 (malignant)	20/78	MN	Histopathology	Sociodemographic (1)
Molina-Vega, M – 2019 (125)	Spain	Cross-sectional	585 (135)	49.4 ± 13.4 (benign) 46.8 ± 15.8 (malignant)	102/483	MN	FNAC and Histopathology	Sociodemographic (2) Clinical (5)
Moosa, NA – 2021 (126)	Kingdom of Bahrain	Cohort (retrospective)	87 (27)	41.1 ± 13.1 (benign) 38.1 ± 10.8 (malignant)	11/76	MN	Histopathology	Sociodemographic (2) Clinical (7)
Mu, N – 2018 (127)	Sweden	Cohort (retrospective)	234 (85)	54 (median - benign) 66 (median - malignant)	71/163	FTC	Histopathology	Sociodemographic (2) Clinical (1)
Muhanhali, D – 2024 (128)	China	Cohort (retrospective)	1,594 (1,225)	49.99 ± 12.30 (benign) 43.54 ± 11.97 (malignant)	475/1,119	PTC	Histopathology	Clinical (1)
Naidu, K – 2023 (129)	South Africa	Cohort (retrospective)	313 (24)	48.0 ± 12.7	63/250	MN	FNAC	Clinical (6)
Nawarathna, NJ – 2018 (130)	Sri Lanka	Cross-sectional	684 (97)	48.0 ± 12.5	73/611	PTC	Histopathology	Sociodemographic (2)
Nguyen, BQ – 2023 (131)	Vietnam	Cross-sectional	272 (42)	48.3 ± 11.8 (benign) 42.2 ± 12.0 (malignant)	27/181	MN	FNAC and Histopathology	Sociodemographic (2) Clinical (6)
Nitipir, C – 2018 (132)	Romania	Cohort (prospective)	189 (47)	48.94 ± 12.7	18/171	MN	Histopathology	Sociodemographic (2)
Nonchev, BI – 2017 (133)	Bulgaria	Cross-sectional	270 (28)	48.89 ± 0.77	21/249	MN	FNAC and Histopathology	Clinical (5)
Öcal, B – 2019 (134)	Turkey	Cohort (retrospective)	233 (141)	46 ± NR	29/204	MN	Histopathology	Sociodemographic (1) Clinical (3)
Ou, D – 2020 (135)	China	Cohort (retrospective)	294 (225)	52.03 ± 14.269 (benign) 46.57 ± 12.729 (malignant)	94/200	FTC	Histopathology	Clinical (8)
Öz, B – 2018 (136)	Turkey	Cohort (retrospective)	80 (23)	45.7 ± 1.6 (benign) 53.6 ± 2.5 (malignant)	21/59	MN	Histopathology	Sociodemographic (1) Clinical (2)
Özdemir, A – 2022 (137)	Turkey	Cohort (retrospective)	221 (108)	51.11 ± 11.06 (benign) 50.84 ± 12.54 (malignant)	44/177	MN	Histopathology	Clinical (2)
Pak, K – 2017 (138)	Korea	Cohort (retrospective)	238 (62)	63 ± NR (benign) 54 ± NR (malignant)	74/164	MN	FNAC	Sociodemographic (2) Clinical (2)
Pappa, T – 2022 (139)	USA	Cohort (prospective)	4,282 (867)	51.0 ± 15.3	1,651/2,631	MN	FNAC and Histopathology	Sociodemographic (2) Clinical (2)
Peng, X – 2020 (140)	China	Cross-sectional	322 (231)	54 ± NR (benign) 46 ± NR (malignant)	61/261	PTC	Histopathology	Sociodemographic (1) Clinical (2)
Peng, B – 2024 (141)	China	Cross-sectional	199 (13)	52.44 ± 10.21 (benign) 42.46 ± 8.75 (malignant)	46/153	MN	Histopathology	Sociodemographic (1) Clinical (3)
Petric, R – 2014 (142)	Slovenia	Cohort (retrospective)	244 (62)	9-82	30/214	MN	Histopathology	Sociodemographic (1) Clinical (1)
Pikis, G – 2021 (143)	Cyprus	Cohort (retrospective)	99 (18)	49.5 ± 13.1 (benign) 48.4 ± 13.7 (malignant)	20/79	MN	Histopathology	Sociodemographic (2) Clinical (8)
Pompili, GG – 2018 (144)	Italy	Cohort (prospective)	231 (17)	59 [48, 70]	67/164	MN	FNAC and Histopathology	Clinical (6)
Qureshi, K – 2022 (145)	Pakistan	Cross-sectional	70 (34)	30 [25.25, 40] (benign) 45 [35, 50] (malignant)	6/64	MN	Histopathology	Clinical (2)
Rago, T – 2014 (146)	Italy	Cohort (retrospective)	1,520 (371)	46.1 ± 13.2		MN	Histopathology	Sociodemographic (2) Clinical (1)
Ramundo, V – 2019 (147)	Italy	Cross-sectional	557 (44)	56.2 ± 14.0		MN	FNAC and Histopathology	Clinical (2)
Ren, T – 2023 (148)	China	Cross-sectional	217 (111)	51.90 ± 10.13 (benign) 44.28 ± 9.46 (malignant)	62/155	MN	FNAC and Histopathology	Clinical (4)
Rianto, BUD – 2019 (149)	Indonesia	Case-control	80 (40)	17-71	30/50	DTC	Histopathology	Clinical (1)
Rios, A – 2016 (150)	Spain	Cohort (prospective)	221 (32)	51.9 ± 16.7	31/190	MN	FNAC and Histopathology	Clinical (3)
Rosenblum, RC – 2019 (151)	Israel	Cohort (retrospective)	128 (18)	56.7 ± 15.5	537/2,137	DTC	FNAC and Histopathology	Sociodemographic (1)
Ryu, YJ – 2014 (152)	Korea	Cohort (retrospective)	116 (41)	43.4 ± 15.1 (benign) 49.8 ± 13.2 (malignant)	25/91	MN	Histopathology	Sociodemographic (1) Clinical (1)
ŞahÜnal, FT – 2024 (153)	Turkey	Case-control (retrospective)	606 (68)	53.53 ± 12.13 (benign) 46.60 ± 13.42 (malignant)	178/428	MN	FNAC and Histopathology	Sociodemographic (2) Clinical (4)
Sahib, AM – 2023 (154)	Iraq	Cohort (prospective)	100 (13)	37.71 ± 11.823 (benign) 37.38 ± 9.614 (malignant)	24/76	MN	Histopathology	Sociodemographic (1)
Sasson, M – 2017 (155)	Canada	Case-control (retrospective)	228 (142)	51 ± NR	58/170	DTC	Histopathology	Clinical (1)
Seo, JW – 2017 (156)	Korea	Cohort (retrospective)	62 (41)	52.95 ± 13.88 (benign) 50.12 ± 12.26 (malignant)	9/53	MN	Histopathology	Sociodemographic (2) Clinical (1)
Shayganfar, A – 2020 (157)	Iran	Cross-sectional	239 (12)	47.74 ± 11.97 (benign) 40.25 ± 7.24 (malignant)	34/205	MN	FNAC	Clinical (1)
Shin, TJ – 2020 (158)	USA	Cohort (retrospective)	166 (25)	52.9 ± 13.2 (benign) 52.2 ± 13.2 (malignant)	39/127	MN	Histopathology	Sociodemographic (3) Clinical (1)
Slijepcevic, N – 2015 (159)	Serbia	Cohort (retrospective)	2,466 (403)	14-85	338/2,128	PTMC	Histopathology	Sociodemographic (2) Clinical (1)
Słowińska-Klencka, D – 2020 (160)	Poland	Cross-sectional	1,000 (162)	54.7 ± 11.6 (benign) 50.3 ± 13.9 (malignant)		MN	Histopathology	Clinical (6)
Sohn, SY – 2014 (161)	Korea	Cohort (retrospective)	3,791 (2,881)	44.1 ± 0.43 (benign) 46.8 ± 0.22 (malignant)	597/3,194	PTC	Histopathology	Sociodemographic (2) Clinical (2)
Song, Q – 2021 (162)	China	Cohort (retrospective)	177 (58)	43.80 ± 12.66 (benign) 45.26 ± 14.64 (malignant)	37/140	MN	FNAC and Histopathology	Clinical (2)
Sun, J – 2023 (163)	China	Cross-sectional	1,998 (1,457)	52 ± NR (benign) 43 ± NR (malignant)	521/1,477	PTC	Histopathology	Clinical (1)
Sutton, W – 2021 (164)	USA	Cohort (retrospective)	443 (24)	18-55	80/363	MN	Histopathology	Sociodemographic (3) Clinical (4)
Swan, KZ – 2018 (165)	Denmark	Case-control (retrospective)	998 (265)	51.2 ± 15.0 (benign) 51.2 ± 15.6 (malignant)	204/794	DTC	Histopathology	Clinical (2)
Talmor, G – 2022 (166)	USA	Cohort (retrospective)	230 (96)	56.0 ± NR (benign) 51.9 ± NR (malignant)	62/168	MN	Histopathology	Sociodemographic (1) Clinical (1)
Tam, AA – 2020 (167)	Turkey	Cohort (retrospective)	2,043 (744)	18-85	448/1,595	MN	Histopathology	Sociodemographic (2) Clinical (1)
Tang, Q – 2023 (168)	China	Cohort (prospective)	77 (41)	50 ± 12 (benign) 42 ± 10 (malignant)	22/55	PTC	Histopathology	Clinical (2)
Teklewold, B – 2022 (169)	Ethiopia	Cohort (retrospective)	110 (32)	41.7 ± NR (benign) 48.3 ± NR (malignant)	12/98	MN	Histopathology	Sociodemographic (2) Clinical (4)
Trimboli, P – 2018 (170)	Italy	Cohort (retrospective)	173 (43)	50 ± 14	46/127	MN	Histopathology	Clinical (1)
Turkyilmaz, S – 2017 (171)	Turkey	Cohort (retrospective)	305 (111)	47.6 ± 11.9 (benign) 49.7 ± 13.0 (malignant)	49/256	MN	FNAC and Histopathology	Sociodemographic (2) Clinical (7)
Tutuncu, Y – 2014 (172)	Turkey	Cohort (retrospective)	201 (69)	47.1 ± 14.1 (benign) 48.9 ± 12.8 (malignant)	23/178	MN	Histopathology	Clinical (4)
Vasileiadis, I – 2014 (173)	Greece	Cohort (retrospective)	836 (389)	51.69 ± 15.26 (benign) 50.89 ± 13.12 (malignant)	167/669	PTC	Histopathology	Sociodemographic (2) Clinical (4)
Wang, L – 2015 (174)	China	Cohort (retrospective)	400 (242)			MN	Histopathology	Clinical (2)
Wang, H – 2018 (175)	China	Cross-sectional	259 (111)	57.4 ± 13.4 (benign) 45.5 ± 12.7 (malignant)	38/143	MN	Histopathology	Clinical (1)
Wang, Z – 2020 (176)	China	Cross-sectional	274 (114)	53 ± NR (benign) 40 ± NR (malignant)	38/236	MN	FNAC and Histopathology	Clinical (4)
Wang, Y – 2021 (177)	China	Cohort (retrospective)	217 (108)	48 [37–57]	59/158	MN	FNAC and Histopathology	Clinical (2)
Wang, G – 2022 (178)	China	Case-control (retrospective)	1,113 (627)	18-77	239/874	PTC	FNAC and Histopathology	Sociodemographic (1) Clinical (5)
Witczak, J – 2016 (179)	UK	Cohort (retrospective)	536 (50)	51.2 ± 15.9 (benign) 50.9 ± 15.9 (malignant)	86/450	MN	FNAC and Histopathology	Sociodemographic (2) Clinical (7)
Wong, SL – 2015 (180)	Australia	Cohort (retrospective)	960 (202)	10-90	205/755	MN	Histopathology	Clinical (1)
Wong, MW – 2017 (181)	Malaysia	Cross-sectional	699 (188)	42.12 ± 13.2 (benign) 47.25 ± 15.3 (malignant)	105/594	MN	Histopathology	Sociodemographic (2) Clinical (1)
Wu, X – 2014 (182)	China	Cohort (retrospective)	2,132 (537)	49.32 ± 0.29 (benign) 44.96 ± 0.58 (malignant)	482/1,650	PTC	Histopathology	Sociodemographic (2) Clinical (2)
Wu, SJ – 2022 (183)	China	Case-control (retrospective)	577 (118)	40.28 ± 13.42 (benign) 47.11 ± 16.68 (malignant)	138/439	FTC	Histopathology	Clinical (4)
Xi, X – 2021 (184)	China	Case-control (retrospective)	279 (50)	50.6 ± 13.2	82/185	MN	Histopathology	Clinical (4)
Xin, Y – 2021 (185)	China	Cohort (retrospective)	262 (76)	48.6 ± 13.0 (benign) 50.1 ± 12.0 (malignant)	63/199	MN	FNAC and Histopathology	Clinical (6)
Xu, SY – 2015 (186)	China	Cohort (retrospective)	2,445 (952)	9-92	606/1,839	MN	Histopathology	Clinical (6)
Xu, DH – 2018 (187)	China	Case-control (retrospective)	220 (155)	54.88 ± 10.67 (benign) 42.08 ± 10.55 (malignant)	220/0	DTC	FNAC and Histopathology	Clinical (1)
Xu, S – 2022 (188)	China	Cohort (retrospective)	712 (453)	49.25 ± 13.06 (benign) 41.66 ± 10.73 (malignant)	205/507	MN	Histopathology	Sociodemographic (2) Clinical (4)
Xu, N – 2022 (189)	China	Cross-sectional	323 (61)	50.3 ± 14.0 (benign) 45.9 ± 13.0 (malignant)	97/226	DTC	FNAC	Sociodemographic (2) Clinical (2)
Yan, HX – 2017 (190)	China	Cohort (retrospective)	7,305 (3,218)	49 ± 12 (benign) 44 ± 12 (malignant)	2,108/5,197	DTC	Histopathology	Sociodemographic (2) Clinical (3)
Yang, TT – 2016 (191)	China	Cross-sectional	96 (74)	49.8 ± 11.3	22/74	MN	Histopathology	Clinical (1)
Yazdani-Charati, J – 2018 (192)	Iran	Cohort (retrospective)	1,817 (169)	41.07 ± 13.81	28/141 (with cancer)	MN		Clinical (3)
Yazgan, A – 2016 (193)	Turkey	Cohort (retrospective)	128		17/111	MN	Histopathology	Clinical (1)
Yazici, P – 2016 (194)	Turkey	Cohort (prospective)	202 (68)	47 ± 12 (benign) 49 ± 12 (malignant)	34/168	DTC	Histopathology	Sociodemographic (2) Clinical (2)
Yeon, EK – 2020 (195)	Korea	Cohort (retrospective)	95 (41)	55.5 ± 13.9 (benign) 47.6 ± 14.5 (malignant)	24/71	MN	FNAC and Histopathology	Sociodemographic (1) Clinical (5)
Yi, D – 2022 (196)	China	Cohort (retrospective)	300 (161)	48.02 ± 12.67 (benign) 41.67 ± 11.14 (malignant)	62/238	MN	FNAC and Histopathology	Clinical (2)
Yoo, MR – 2015 (197)	Korea	Cohort (retrospective)	67 (49)	50 ± 10 (benign) 47 ± 11 (malignant)	10/57	MN	FNAC and Histopathology	Clinical (3)
Yoon, JH – 2014 (198)	Korea	Cohort (retrospective)	275 (83)	50 ± 12 (benign) 47 ± 11 (malignant)	47/228	MN	FNAC and Histopathology	Sociodemographic (1) Clinical (5)
Yu, Z – 2021 (199)	China	Cross-sectional	1159 (285)	39.94 ± 12.10	327/1,014	PTC	FNAC and Histopathology	Sociodemographic (2)
Yu, B – 2022 (200)	China	Cross-sectional	129 (28)	4-84	43/86	MN	Histopathology	Sociodemographic (2) Clinical (5)
Zeng, R – 2016 (201)	China	Case-control (retrospective)	1,198 (578)	9-86	181/1,017	PTC	Histopathology	Sociodemographic (4) Clinical (4)
Zhang, YF – 2014 (202)	China	Cross-sectional	173 (96)	54 ± 8 (benign) 49 ± 11 (malignant)	35/122	MN	Histopathology	Sociodemographic (1) Clinical (2)
Zhang, H – 2014 (203)	China	Cross-sectional	71 (32)	50.5 ± 9.1	16/43	MN	Histopathology	Clinical (3)
Zhang, J – 2015 (204)	Singapore	Cross-sectional	388 (27)	54 ± 14 (benign) 48 ± 16 (malignant)	69/319	MN	FNAC and Histopathology	Sociodemographic (2) Clinical (9)
Zhang, X – 2018 (205)	China	Cohort (retrospective)	500 (250)	49.92 ± 12.36 (benign) 48.05 ± 13.87 (malignant)	106/394	DTC	Histopathology	Clinical (2)
Zhang, F – 2019 (206)	USA	Cohort (retrospective)	188 (14)	25-97	26/162	MN	FNAC and Histopathology	Sociodemographic (3) Clinical (4)
Zhang, X – 2022 (207)	China	Cohort (retrospective)	2,090 (1,519)	50.77 ± 13.09 (benign) 43.22 ± 12.38 (malignant)	546/1,544	PTC	Histopathology	Sociodemographic (1) Clinical (2)
Zhang, F – 2024 (208)	China	Cohort (retrospective)	645 (115)	46.5 ± 13.8 (benign) 46.1 ± 15.6 (malignant)	159/486	FTC	Histopathology	Clinical (6)
Zhao, RN – 2015 (209)	China	Cross-sectional	83 (58)	49.20 ± 10.01 (benign) 44.20 ± 10.25 (malignant)	22/61	MN	FNAC and Histopathology	Sociodemographic (1) Clinical (2)
Zhao, H – 2018 (210)	China	Cohort (retrospective)	2,041 (1,120)	F: 49.7 ± 11.08 (benign) F: 45.1 ± 10.6 (malignant) M: 50.6 ± 12.5 (benign) M: 44.6 ± 11.9 (malignant)	469/1,572	PTC	Histopathology	Sociodemographic (1) Clinical (4)
Zhao, S – 2019 (211)	China	Case-control (retrospective)	9,765 (5,114)	50.03 ± 11.66 (benign) 44.21 ± 10.97 (malignant)	2,816/6,949	PTC	Histopathology	Sociodemographic (2) Clinical (3)
Zhao, RN – 2019 (212)	China	Cohort (prospective)	367 (217)	49.6 ± 9.9 (benign) 43.1 ± 10.9 (malignant)	84/223	MN	Histopathology	Sociodemographic (1) Clinical (7)
Zhao, Z – 2022 (213)	China	Cross-sectional	86 (44)	≥18		PTC	Histopathology	Sociodemographic (2) Clinical (1)
Zivic, R – 2016 (214)	Serbia	Case-control (retrospective)	256 (114)	15-77 (benign) 26-89 (malignant)	48/208	HCC	Histopathology	Clinical (1)
Zoric, GV – 2020 (215)	Serbia	Case-control (retrospective)	263 (166)	19-79 (benign) 15-78 (malignant)	54/209	FTC	Histopathology	Sociodemographic (2) Clinical (7)

DRC, Democratic Republic of Congo; DTC, differentiated thyroid cancer; F, female; FNAC, fine-needle aspiration cytology; FTC, follicular thyroid cancer; HCC, Hurthle cell cancer; M, male; MN, malignant nodules; NR, not reported (standard deviation); OFTC, oxyphilic follicular thyroid carcinoma; PTC, papillary thyroid cancer; PTMC, papillary thyroid microcarcinoma; UK, United Kingdom; USA, United States of America; WDTC, well-differentiated thyroid cancer.

#### 3.2.1 Location

The included studies were geographically diverse, with a predominance of research conducted in Asia (126 out of 194 studies). Europe contributed with 41 studies, followed by North America with 19. Fewer studies originated from Africa (5), South America (2), and Australia (1).

#### 3.2.2 Study design

Cohort studies represented the majority of included articles, with a total of 138. Cross-sectional studies accounted for 42, whereas 14 followed a case-control design.

#### 3.2.3 Population and thyroid nodules diagnosis

Studies enrolling only adults were more common than those with mixed-age populations (i.e., adults and children), though both were well represented among the included evidence. No study was restricted exclusively to a pediatric population.

Gender distribution was reported in 185 of the 194 studies. Most studies reported a higher number of female than male participants. Two studies enrolled only women, 1 included only men, and in 1 study the number of male participants exceeded that of females.

Across included studies, 140 investigated thyroid malignancy as a general outcome without specifying histological subtype. Several others focused on specific types: 28 on PTC, of which 4 specifically addressed papillary thyroid microcarcinoma (PTMC); 11 on FTC, with 1 study examining exclusively the oncocytic variant (OFTC). DTC was the focus in 12 studies, while 1 investigated well-DTC (WDTC). Hurthle cell cancer (HCC) was addressed in just 1 study. Another study considered a mixed group comprising general malignancy, PTC, PTMC, and HCC.

Of the 193 studies reporting the diagnostic method, the majority (127) relied on histopathological confirmation. FNAC alone was used in 11 studies, while both histology and FNAC were employed in 55.

### 3.3 Predictors of malignancy in thyroid nodules

**Supplementary Table 1** provides an overview of the potential predictors of malignancy examined across included studies. The most frequently evaluated variables were age (108 studies), sex (92 studies), nodule size (87 studies), and calcifications (85 studies). To ensure consistency and interpretability of findings, only predictors assessed in five or more studies were considered in the descriptive synthesis below.

Effect estimates for eligible predictors, those assessed in more than 5 studies, were extracted regardless of inclusion in meta-analysis (**Supplementary Material**). Most studies reported ORs. However, four reported HRs (52, 68, 152, 164) and two reported RRs (29, 154). For quantitative synthesis, only crude ORs were pooled, as specified in the Methods. Predictors assessed in fewer than 5 studies are listed in the **Supplementary Material** and were not analysed further.

A detailed description of each predictor is provided in the following sections.

#### 3.3.1 Age

**Figure 2:**
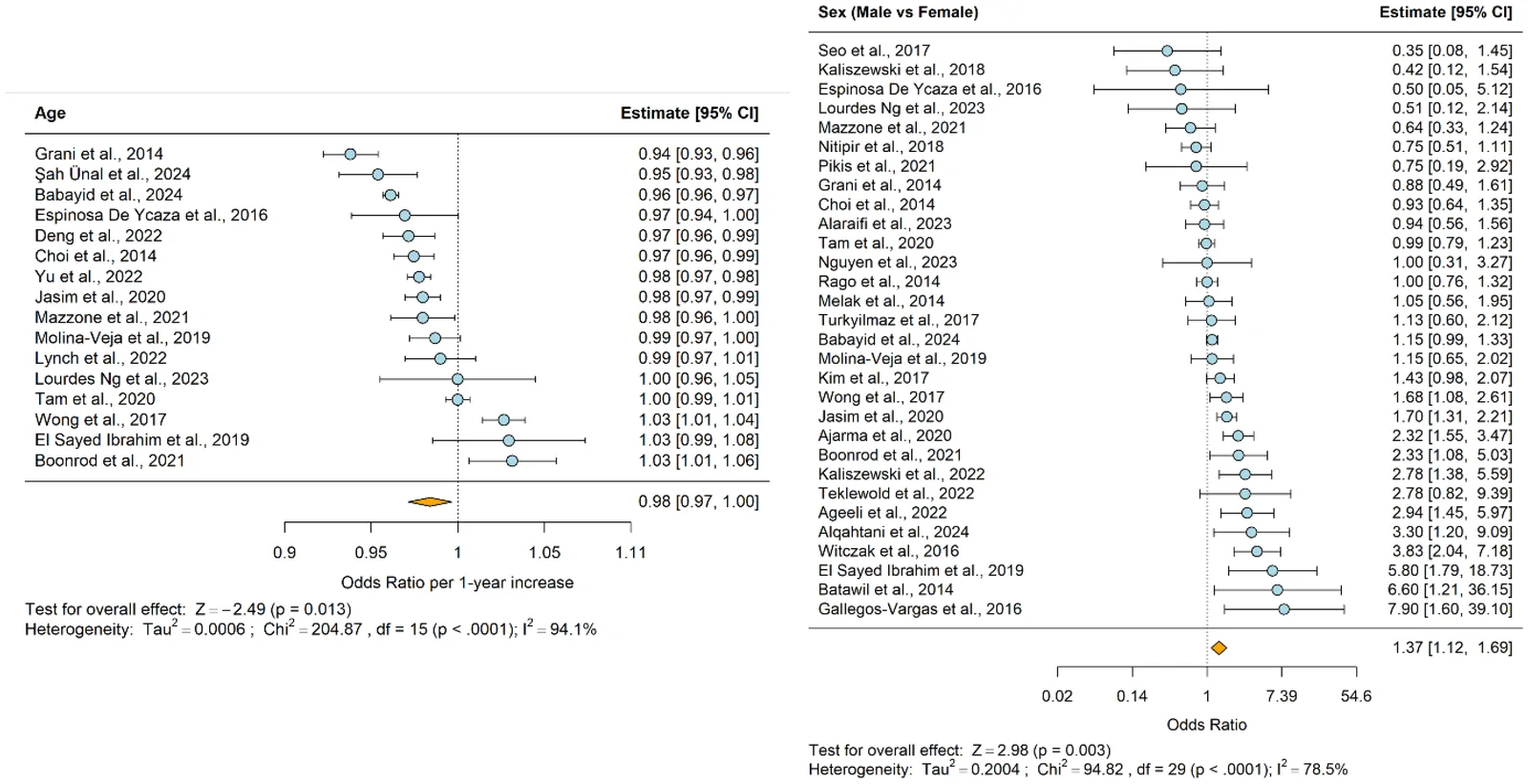
Forest plots of individual and pooled estimates for the associations between age (per 1-year increase) and malignancy (left) and sex and malignancy (right).

Age was evaluated heterogeneously across studies, either as a continuous variable or using categorical thresholds. Considering studies reporting statistically significant associations, increasing age was often associated with lower odds of malignancy, although exceptions were reported (38, 43, 63, 122, 136, 161, 171, 181, 189). In a meta-analysis restricted to studies treating age as a continuous predictor (per year), a statistically significant association between increasing age and decreased odds of malignancy emerged (OR = 0.98; 95% CI: 0.97-1.00), with high heterogeneity (I² = 94.1%) - **Figure 2**, left forest plot. In meta-regression, diagnostic method surged as a significant moderator (p = 0.024), explaining 22.9% of between-study heterogeneity. When stratified by diagnostic approach, the inverse association between age and malignancy was significant only in studies using both histopathology and FNAC (β = -0.0259, p = 0.0240; **Supplementary Figure 2**).

#### 3.3.2 Sex

Male sex was consistently associated with thyroid malignancy across studies, except for Chiefari et al. (50) and Du et al. (62), who observed increased risk in females. As demonstrated in **Figure 2** (right forest plot), meta-analysis indicated a statistically significant association (OR = 1.37; 95% CI: 1.12-1.69), with high heterogeneity (I² = 78.5%) unexplained by moderator variables. However, leave-one-out sensitivity analyses confirmed robustness of the pooled estimate (**Supplementary Table 2**).

#### 3.3.3 Body mass index

Most studies reported no significant association between body mass index (BMI) and malignancy, whereas those reporting an association demonstrated heterogeneous patterns. Arduc et al. (32) observed an overall increased risk with higher BMI, a pattern also seen by Xu et al. (187), but limited to DTC. In PTC, Huang et al. (86), and Zhao et al. (211) reported that BMI above 24 kg/m^2^ was associated with increased risk, while Crudele et al. (55) found that BMI above 29 kg/m^2^ increased overall malignancy risk. Yan et al. (190), exploring DTC, described a U-shaped pattern, with BMI between 24 and 28 kg/m^2^ linked to lower risk, and BMI below 18.5 kg/m^2^ or above 28 kg/m^2^ associated with higher risk. Due to heterogeneous BMI reporting and categorization, meta-analysis was not feasible.

#### 3.3.4 Family history of thyroid cancer

**Figure 3:**
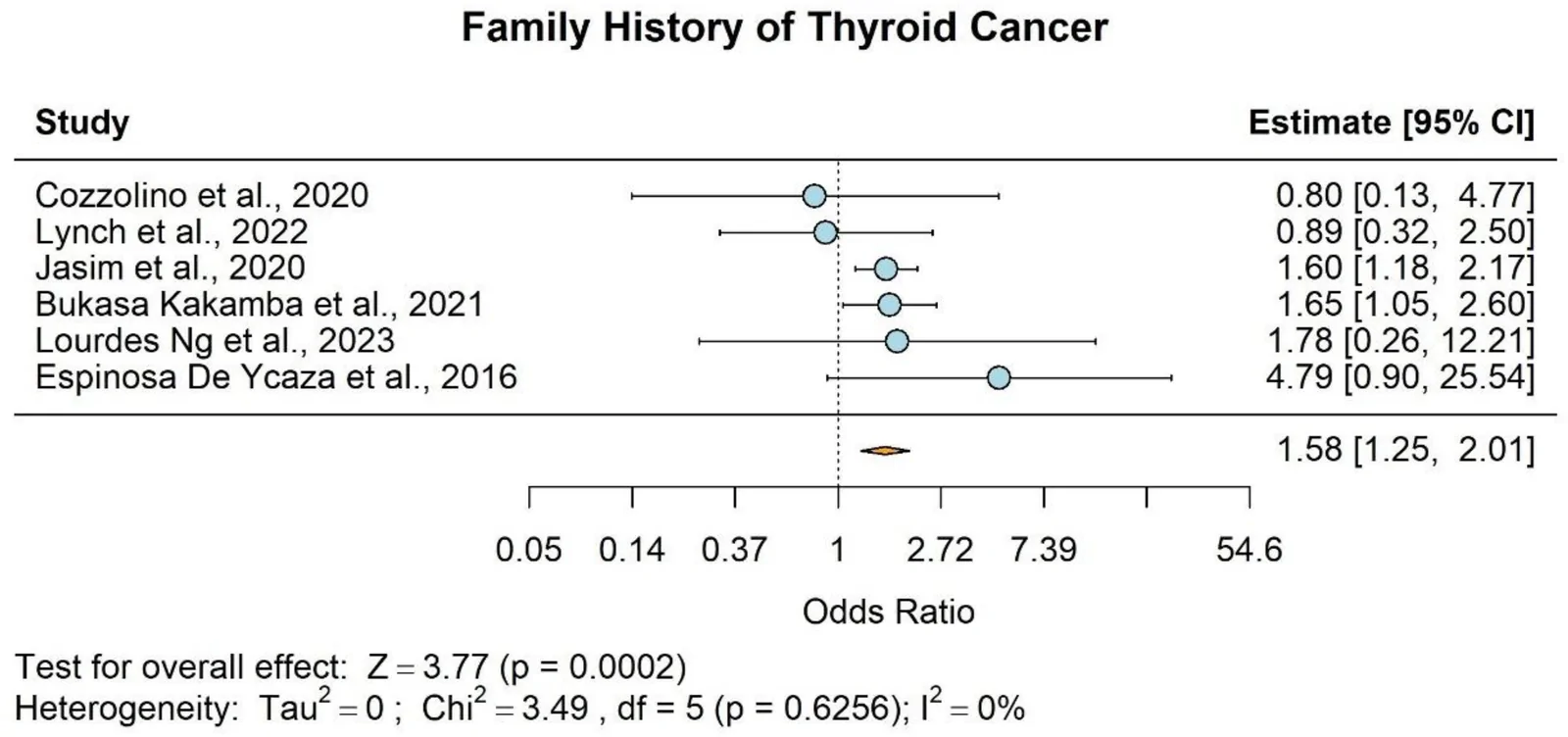
Forest plots of individual and pooled estimates for the associations between family history of thyroid cancer and malignancy.

The impact of a family history of TC on nodule malignancy has been explored in multiple studies, with most finding no significant relationship (40, 41, 54, 65, 69, 88, 117, 118, 126, 164). An exception was observed by Jasim et al., which reported an increased likelihood of malignancy among individuals with a family history of TC (91). In meta-analytic pooling of six studies (**Figure 3**), a significant effect was detected (OR = 1.58; 95% CI: 1.25–2.01), suggesting that TNs in patients with affected relatives are more prone to malignancy. Heterogeneity was negligible (I² = 0%), and leave-one-out sensitivity analyses confirmed robustness - **Supplementary Table 3**. Given the consistency of results, no further subgroup analyses were conducted.

#### 3.3.5 Compressive symptoms

Several studies reported no significant link between the presence of symptoms such as hoarseness and malignant potential (41, 117, 126, 164), whereas others observed a potential association. Teklewold et al. found that nodules causing compressive complaints were more likely to be malignant (169), whereas Canete et al. reported that the absence of such symptoms was unexpectedly associated with higher malignancy risk (42).

#### 3.3.6 Thyroid hormones: TSH, Tg, FT3 and FT4

**Figure 4:**
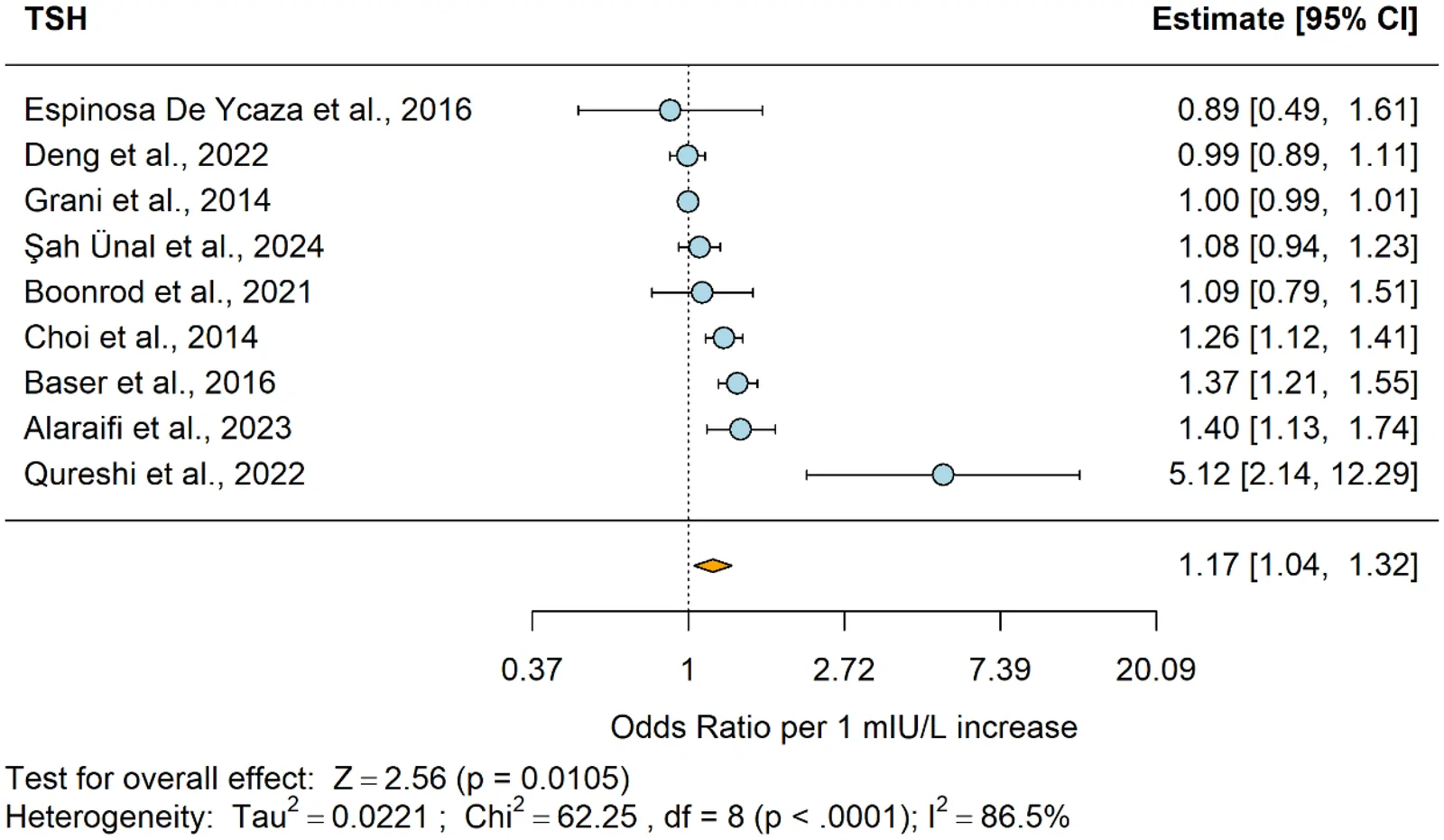
Forest plots of individual and pooled estimates for the associations between TSH levels (per 1 mIU/L increase) and malignancy.

While several studies reported that higher TSH levels were associated with increased odds of malignancy (15, 16, 35, 45, 64, 70, 71, 145, 149, 161, 163, 165, 173, 174, 179, 182, 192, 205, 210), others found no association (32, 38, 48, 52, 53, 58, 61, 62, 69, 75, 78, 88, 92, 116, 153, 171, 194, 211). Some authors suggested a protective effect of low TSH (27, 107, 155), whereas levels above specific thresholds consistently increased risk (96, 112, 190, 201). Evidence for Tg, Free Triiodothyronine (FT3), and Free Thyroxine (FT4) was more variable. Elevated Tg was linked to both increased (100, 112, 142, 192, 214, 215) and decreased risk (120, 201, 207, 213). Higher FT3 sometimes correlated with reduced odds of malignancy (182, 192, 213), while increased FT4 or altered FT4/FT3 ratios were occasionally associated with higher odds (155, 192). In a meta-analysis restricted to studies treating TSH as a continuous predictor (per 1 mIU/L increase), higher TSH was associated with higher odds of malignancy (OR = 1.17; 95% CI: 1.04–1.32), as reported in **Figure 4**. Between-study heterogeneity was high (I² = 86.5%). Meta-regression revealed that study design accounted for 24.3% of between-study heterogeneity, with cross-sectional studies showing stronger associations (β = 1.50; p=0.0013) than cohorts. Additionally, diagnostic method explained 65.5% of residual heterogeneity. Studies employing both histopathology and FNAC presented weaker associations (β = -0.26; p=0.0044) compared to those using only histopathology. Stratified meta-regression results are shown in **Supplementary Figure 3**.

#### 3.3.7 Thyroid antibodies: TgAb and TPOAb

Evidence regarding the association between thyroid antibodies and malignancy remains heterogeneous. Studies evaluating thyroid antibodies in general often pointed to a higher likelihood of malignancy (35, 61, 85, 180, 182), although others failed to confirm this link (62, 75). For Tg antibodies (TgAb), most studies indicated an association with malignant disease (48, 92, 112, 116, 140, 173, 174, 201, 205, 210), whereas several did not detected a significant effect (145, 167, 189, 194). By contrast, the role of Thyroid Peroxidase antibodies (TPOAb) was less consistent. Some authors suggested a contributory role (87, 107, 112, 182, 201, 210), while others observed neutral results (48, 92, 101, 116, 145, 167, 189, 194, 215). Huang et al. reported an unexpectedly inverse association for elevated TgAb in PTC (87).

#### 3.3.8 Nodule location

Nodule location was explored as a potential predictor, though reporting varied widely across studies. Isthmus location was consistently associated with higher odds of malignancy (24, 28, 91), with Jasim et al. further ranking risk by location: isthmus > upper pole > middle lobe (91). Upper pole nodules were also linked with an increased odds in several studies (28, 153, 206, 216), while middle lobe nodules showed variable associations, with Ramundo et al. (147), and Şah Ünal et al. (153) reporting increased malignancy risk. TNs confined to a single lobe were associated with higher risk (112), whereas bilateral nodules were linked to increased risk and those in the right or left lobes with decreased risk (192). Substernal nodules were also reported to carry higher risk (41). Heterogeneity in location definitions and reporting limited quantitative pooling.

#### 3.3.9 Nodule size

**Figure 5:**
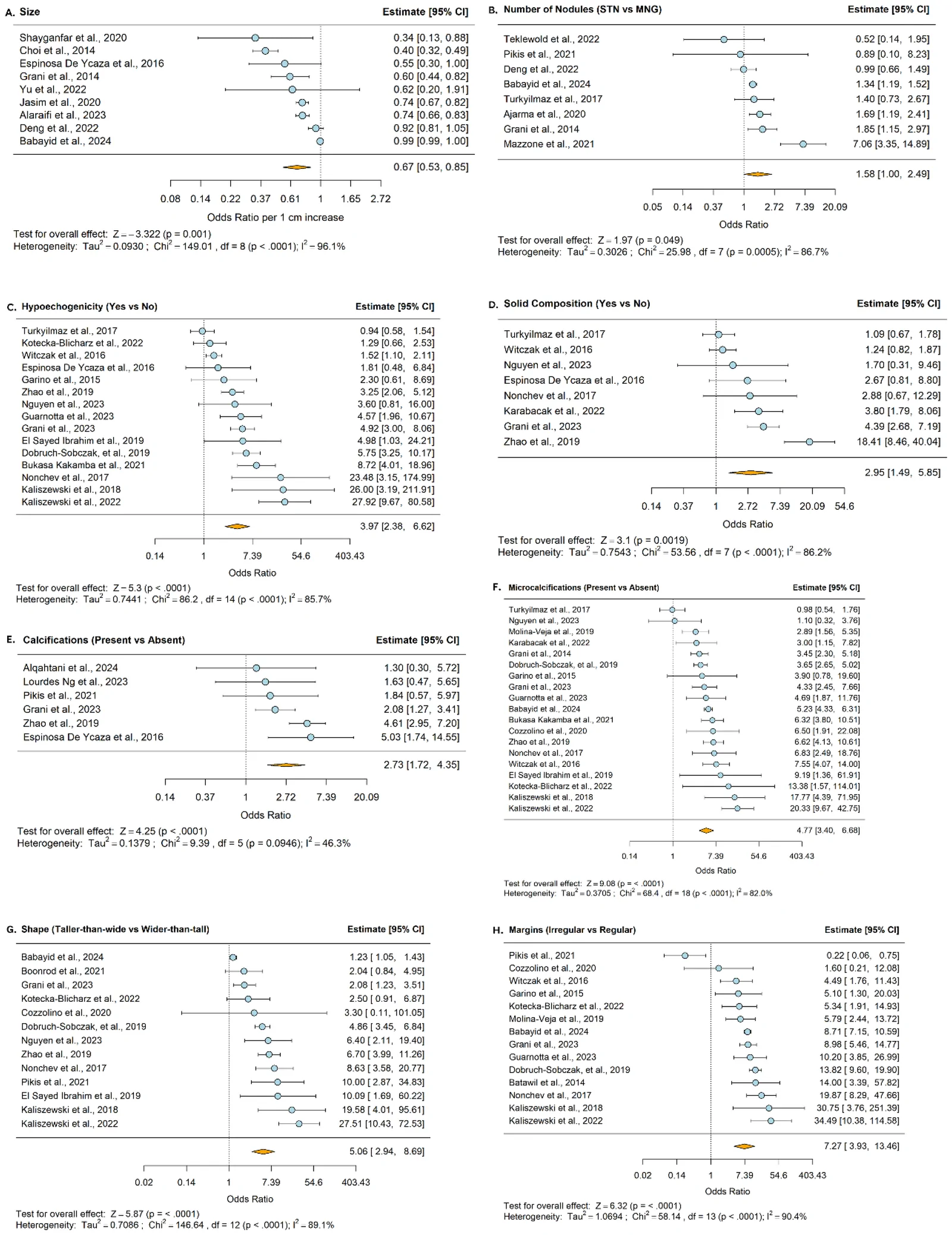
Forest plots showing individual and pooled estimates of the associations between analysed ultrasonographic features and malignancy. For nodule size **(A)**, effects are expressed per 1 cm increase. **(A)**, Size; **(B)**, number of nodules (STN vs MNG); **(C)**, hypoechogenicity (yes vs no); **(D)**, solid composition (yes vs no); **(E)**, calcifications (present vs absent); **(F)**, microcalcifications (present vs absent); **(G)**, shape (taller-than-wide vs wider-than-tall); **(H)**, margins (irregular vs regular).

Nodule size associations with malignancy were inconsistent across studies. Several authors reported that increasing size was associated with a higher probability of malignancy, either as a continuous measure (30, 38, 43, 119, 127, 191) or above specific thresholds such as 10 mm (40), 30 mm (141), or 40 mm (110, 118, 139, 169, 170). Conversely, multiple studies, particularly in PTC, observed the opposite, with larger nodules associated with decreased odds of malignancy (16, 34, 42, 61, 69, 87, 92, 123, 157, 207, 210, 211). Other authors suggested that smaller nodules (typically <1-1.5 cm) carried higher odds (35, 49, 51–54, 78, 85, 112, 125, 156, 168, 178). Rapid nodule growth (>2 mm/year or accelerated expansion) was consistently linked to increased malignancy risk (29, 93, 95). Given variability in reporting, meta-analysis was restricted to studies assessing nodule diameter as a continuous predictor. Effect estimates were rescaled to a common unit (per 1 cm increase) prior to pooling. Meta-analysis revealed high between-study heterogeneity (I^2^ > 90%), alongside a significant inverse association between increasing nodule diameter and malignancy (OR = 0.67; 95% CI: 0.53-0.85) - **Figure 5A**. Meta-regression indicated that the number of nodules per study explained nearly all the observed variability (R² = 99.2%, p < 0.0001), with studies with less than 514 nodules under analysis showing a stronger and statistically inverse association (β = -0.3098, p < 0.0001, **Supplementary Figure 4**), with no residual heterogeneity within this subgroup (I^2^ = 0%).

#### 3.3.10 Nodules number

The relationship between the number of TNs and malignancy has also been explored. Solitary TNs (STN) were often reported as carrying higher odds of malignancy, including DTC and FTC (34, 45, 63, 71, 88, 121, 122, 190), whereas multinodular goiter (MNG) or multiple nodules often presented reduced odds (30, 77, 192). Nevertheless, some studies described divergent findings, with multiple nodules being associated with increased malignancy risk in specific contexts, such as FTC or in female patients (112, 183, 210, 215), or risk varying according to nodule function and size (159, 164). In the quantitative synthesis represented in **Figure 5B**, comparing solitary versus multiple nodules, high between-study heterogeneity was observed (I² = 86.7%). STNs showed a borderline association with malignancy (OR = 1.58; 95% CI: 1.00-2.49). Meta-regression identified continent as a significant moderator (p = 0.037), explaining 60.2% of variability, as seen in **Supplementary Figure 5**. Asian studies exhibited a stronger, though non-significant, effect (β = 0.943, p = 0.229), while those from Europe demonstrated a significantly higher effect (β = 1.719, p = 0.036). Even after adjustment, residual heterogeneity remained substantial (I^2^ = 74.3%).

#### 3.3.11 Echogenicity

Echogenicity was one of the most consistently reported US predictors of malignancy. Hypoechogenic and markedly hypoechogenic nodules were repeatedly associated with higher malignancy rates (33, 34, 37, 40, 57, 60, 62, 67, 74, 76, 77, 80, 83–85, 90, 94–96, 106, 109–111, 113, 116, 129, 131, 133, 137, 141, 144, 148, 150, 160, 172, 176, 178, 184, 186, 193, 198, 202–204, 208, 212). Conversely, isoechogenic or hyperechogenic nodules tended to carry a lower risk (98, 123, 200). However, few studies described more complex patterns, such as risk increases associated with mixed echogenicity or population-specific patterns (57, 141, 148, 150). Meta-analysis focusing on the presence versus absence of hypoechogenicity confirmed a significant association with malignancy (OR = 3.97; 95% CI: 2.38-6.62), which remained robust across study subgroups - **Figure 5C**. Although the diagnostic methods explained a small portion of variability (R² = 12.9%), subgroup differences did not reach statistical significance (p = 0.1574). Residual heterogeneity remained high (I^2^ = 82.6%).

#### 3.3.12 Composition

Solid nodules, whether purely or predominantly solid, carried higher malignancy probability (30, 34, 36, 40, 60, 62, 76, 77, 80, 85, 90, 97, 109, 110, 113, 114, 125, 126, 137, 144, 147, 160, 172, 184, 186, 208), whereas cystic or mixed (solid-cystic) nodules showed comparatively lower malignancy rates (30, 75, 200, 204). Few studies, however, reported increased malignancy risk in mixed nodules or even in cystic composition under specific contexts (34, 135). As shown in **Figure 5D**, meta-analysis confirmed that solid composition was associated with higher odds of malignancy (OR = 2.95; 95% CI: 1.49-5.85), despite high heterogeneity (I² = 86.2%). Meta-regression suggested that studies employing both histopathology and FNAC reported a weaker effect (β = -1.43; p = 0.0221) than those using histopathology alone, as seen in **Supplementary Figure 6**. Despite the diagnostic method explained 44.67% of variability, residual heterogeneity remained high (I² = 77.6%).

#### 3.3.13 Calcifications

Among calcification patterns, microcalcifications emerged as the most consistent marker of malignancy (37, 39, 40, 42, 54, 59, 60, 62, 67, 75, 77, 79, 84, 87, 90, 93–98, 103, 104, 106, 109, 110, 113–116, 123, 125, 129, 133–136, 144, 150, 160, 162, 166, 172, 176, 177, 179, 184–186, 188, 198, 204, 212), whereas macrocalcifications were less predictive and were occasionally related to benign nodules (57, 60, 99, 135, 204). The pooled meta-analysis represented by **Figure 5E** confirmed calcifications a significant predictor (OR = 2.73; 95% CI: 1.72-4.35), with moderate heterogeneity (I² = 46.3%). Meta-regression further indicated a significant temporal effect (p <0.0001, R² = 100%), with more recent studies (2020–2024) reporting weaker associations than earlier ones (β = -0.8861, p = 0.0028) – **Supplementary Figure 7**. When microcalcifications were analysed separately (**Figure 5F**), the association remained robust (OR = 4.77; 95% CI: 3.40-6.68), though with high heterogeneity (I² = 82.0%). Subgroup analysis by diagnostic method, depicted in **Supplementary Figure 8**, explained 23.0% of heterogeneity, with studies combining histopathology and FNAC showing weaker estimates (β = -0.7052, p = 0.0265) compared with those using histopathology alone.

#### 3.3.14 Nodule shape

Taller-than-wide (TTW) nodules consistently presented a higher probability of malignancy (34, 59, 60, 62, 67, 77, 79, 84, 94–96, 98, 104, 114, 131, 133, 176, 178, 185, 186, 188, 195, 202–204, 209, 212), as did nodules with spherical or irregular shapes, or with a non-parallel orientation (85, 139, 175, 196, 198). Wider-than-tall orientation demonstrated lower malignancy rates (200). As represented in **Figure 5G**, meta-analysis confirmed that TTW morphology was significantly associated with higher odds of malignancy (OR = 5.06; 95% CI: 2.94–8.69), despite high heterogeneity (I² = 89.1%), which remained unexplained by moderator analyses. Leave-one-out analyses (**Supplementary Table 4**) indicated that no single study substantially altered the significance or direction of the association, supporting the robustness of the findings.

#### 3.3.15 Margins

Irregular or ill-defined margins were consistently associated with higher odds of malignancy (34, 36, 37, 39, 42, 57, 60, 62, 73, 76–78, 82, 84, 85, 94, 95, 103, 109, 112, 125, 133–135, 143, 148, 160, 162, 168, 176–179, 184–186, 188, 196–198, 203, 204, 208, 212), while well-defined margins tended to be associated with lower malignancy rates (200). Variants such as microlobulated, spiculated, or obscure borders also demonstrated a significant correlation with malignancy (82, 185, 186, 204). Meta-analysis reinforced these findings, showing a strong association between irregular margins and malignancy (OR = 7.27; 95% CI: 3.93–13.46), although with high heterogeneity (I² = 90.4%) – **Figure 5H**. Leave-one-out analyses identified the study by Pikis et al. (143), whose exclusion reduced heterogeneity to 40.8% while maintaining the significance and direction of the effect, as shown in **Supplementary Table 5**. Exploratory subgroup analysis based on the number of nodules evaluated per study partially explained the variability (R² = 14.2%), though the moderator did not reach statistical significance (p = 0.13).

#### 3.3.16 Vascularity

Increased intranodular or central vascularity was frequently linked to malignancy (73, 77, 78, 85, 98, 104, 109, 110, 116, 150, 160, 172, 186, 188, 208, 209, 212). Chaotic or mixed vascular patterns have also been shown to elevate malignancy risk (150, 172, 212). In contrast, nodules exhibiting predominantly peripheral blood flow or absent/weak intralesional flow were associated with a lower likelihood of malignancy (54, 77, 123, 188).

#### 3.3.17 Halo

The predictive value of a peripheral halo around TNs remains uncertain. Some authors reported that a thick, irregular, or absent halo may signal an increased risk of malignancy (28, 113, 183, 208, 212), whereas others described protective features, with thin or hypoechoic halos more often linked to benignity in some subgroups, particularly in FTC (183, 204). In contrast, other studies did not observe any meaningful association between halo characteristics and malignancy (54, 78, 80, 135, 171).

#### 3.3.18 Echotexture

The diagnostic relevance of echotexture appeared limited. Several studies reported no meaningful correlation between heterogeneous or equal echotexture and malignancy, including analyses restricted to PTC and FTC (28, 99, 135, 153, 183). By contrast, isolated findings suggested that absent or markedly reduced echotexture may be associated with a higher likelihood of malignancy (112, 135).

## 4 Discussion

The role of sociodemographic and clinical factors in predicting thyroid malignancy has long been a subject of interest within the research community. This systematic review and meta-analysis synthesizes evidence on sociodemographic and clinical factors associated with malignancy in TNs. Rather than suggesting that any single factor is determinative, our findings supported a tiered interpretation: i) several US features show the most consistent and clinically actionable associations; ii) a smaller set of patient-level and biochemical variables provide complementary context (notably sex, family history, and TSH); and iii) many other variables show heterogeneous or setting-dependent associations that are less suitable for standalone decision-making. Because several predictors showed substantial heterogeneity between studies, our pooled estimates are best interpreted showing a consistent direction of association, while the exact size of the effect may differ across populations and clinical settings.

Across studies, the strongest and most reproducible associations were observed for established suspicious US features, such as hypoechogenicity, solid composition, microcalcifications, TTW shape, and irregular margins, which are already incorporated into existing risk-stratification systems such as ATA guidelines, ACR-TIRADS, and EU-TIRADS (7–9, 217). However, these systems classify nodules into risk categories based on expert consensus and describes each feature’s contribution to malignancy risk in categorical rather than quantitative terms. Our meta-analysis extends this evidence in three complementary ways. First, by pooling data from a large and heterogeneous population, we provide updated effect estimates with greater precision for each US feature, which may help refine the weight assigned to individual features in existing systems. Second, we demonstrate that these associations are consistent across different study populations, diagnostic settings, and reference standards, as supported by leave-one-out sensitivity analysis. Third, we simultaneously evaluate patient-level and biochemical variables that are not part of current sonographic systems, offering a more integrated view of malignancy risk. The suspicious US features likely reflect underlying tissue characteristics of malignant nodules, such as increased cellularity/fibrosis, infiltrative growth, and psammoma bodies in PTC, and their value is greatest when interpreted in combination (218–224). The robustness of these associations across sensitivity analyses supports their continued role as the backbone of pre-biopsy risk assessment.

Patient-level variables added incremental information not captured by current ultrasound-based systems, which do not formally include factors such as sex or family history. Rather than acting as independent predictors of malignancy, these variables may be more useful as factors that adjust the pre-test probability of malignancy, complementing rather than replacing ultrasound-based risk stratification. Male sex was associated with higher odds of malignancy despite the higher overall incidence of TC in women (11). This pattern may reflect a mixture of biological differences and clinical pathways, including presentation and diagnostic intensity (225–228). Family history of TC showed a modest but statistically significant association with malignancy. Although most individual studies did not reach statistical significance, this likely reflects limited statistical power at the study level rather than conflicting effects, as most point estimates were directionally consistent (OR >1). Leave-one-out analysis confirmed that this result does not depend on any single study. Excluding the largest study, by Jasim et al. (91), still yielded a significant pooled estimate (OR = 1.55; 95% CI: 1.06-2.29). Pooling therefore increases precision around a consistent underlying effect, explaining the negligible heterogeneity observed (I^2^ = 0%), although we cannot exclude a contribution from surveillance bias, as heightened clinical monitoring in relatives of TC patients may increase nodule detection independently of true biological risk (229). Both mechanisms may coexist and should be considered when translating pooled estimates to clinical practice.

Among laboratory markers, TSH may similarly function as an adjunctive rather than independent factor. Higher TSH (per 1 mIU/L increase) was associated with higher odds of malignancy. While a biologically plausible link exists through trophic stimulation of follicular cells (230), heterogeneity was substantial and partly explained by study design and diagnostic reference standard. This suggests that biomarker-outcome associations are sensitive to how populations are selected and how malignancy is defined and confirmed (16, 231). In contrast, evidence for FT3, FT4, and Tg was inconsistent and often study-specific, limiting synthesis and clinical applicability. Similarly, thyroid autoantibodies showed heterogeneous patterns. TgAb was more often reported as positively associated with malignancy, whereas TPOAb findings were inconsistent and may be strongly influenced by autoimmune thyroiditis and its complex relationship with cancer detection and tumor phenotype (11, 232). Overall, these laboratory variables appear adjunctive rather than primary decision drivers.

Patient-level and biochemical variables are likely to add clinical value in specific scenarios. First, in TNs with intermediate ultrasound risk (7–9), where the decision between FNAC and active surveillance is often uncertain, factors such as male sex, a first-degree family history of TC, or elevated TSH may support the decision to biopsy, while their absence may favour continued surveillance. Second, in cases of discordance between clinical suspicion and sonographic appearance, for instance, a clinically suspicious nodule with only mildly suspicious US features, these variables provide contextual information that ultrasound alone cannot capture. Third, in subcentimetric TNs that fall below current size thresholds for FNAC, these factors may help identify patients who could still benefit from earlier cytological evaluations (7). Conversely, in TNs with clearly benign or unequivocally malignant sonographic patterns, patient-level variables are unlikely to alter management. Identifying the situations in which these factors influence clinical decisions, rather than applying them uniformly, will be essential for their future integration in risk-stratification systems.

Some associations observed in TN-based cohorts are difficult to interpret as “risk factors” in a causal sense because of selection effects. For example, increasing age (per year) and larger nodule size (per 1 cm increase) were associated with lower odds of malignancy in pooled analyses, but both showed marked heterogeneity. These patterns may reflect how nodules enter diagnostic pathways: younger patients may undergo more intensive work-up, and smaller nodules can be preferentially selected for biopsy when they carry high-risk US features, whereas larger nodules may be evaluated or treated because of size-related symptoms or other clinical considerations even when ultrasound suspicion is lower (7, 217, 233, 234). This is consistent with current nodule risk-stratification practice, where sonographic features, rather than age or size alone, guide biopsy decisions (7). More broadly, differences between cytology-based and surgical series can amplify such selection effects (45). Our meta-regression results support this interpretation, as diagnostic method and study size (number of nodules included) explained substantial proportions of heterogeneity for some predictors. The need to rescale nodule size units prior to pooling further illustrates how seemingly minor reporting differences can materially affect synthesis and underscores the importance of standardized measurement and reporting.

Solitary versus multiple nodules showed only a borderline pooled association, with geographic differences. This may reflect differences in diagnostic practices (e.g., selective biopsy of the dominant nodule in multinodular goitre), underlying iodine status, and thresholds for surgery or cytology rather than a uniform biological difference between solitary nodules and multinodular disease (235–237). Future studies that consistently define the unit of analysis (patient vs nodule) and apply comparable reference standards will be better positioned to clarify these relationships.

Compressive symptoms and several secondary US features, including vascularity, halo, and echotexture, showed inconsistent associations. Symptom-based associations are particularly vulnerable to confounding by nodule size and thyroid volume and to variability in symptom ascertainment (233). For US features such as vascularity and halo, inconsistent definitions, operator dependence, and variation in ultrasound equipment and reporting lexicon likely contribute to mixed findings (114, 223, 238). These predictors may still be informative in specific clinical contexts, but current evidence does not support treating them as universally reliable markers.

Taken together, our findings support a pragmatic approach to pre-biopsy assessment: prioritize structured US patterns (especially hypoechogenicity, solid composition, microcalcifications, TTW shape, and irregular margins), integrate patient-level context (sex and family history), and consider TSH as an adjunctive marker while acknowledging heterogeneity and potential confounding. Importantly, these pooled associations should be seen as correlational. For predictors with high heterogeneity, the direction of the effect is more reliable than its exact magnitude, which may vary with local disease prevalence, referral pathways, and diagnostic practice.

For future research, the most actionable gaps relate to standardization and design. Studies should i) adopt standardized ultrasound lexicons and explicit definitions for each feature; ii) report continuous predictors with clear units and modelling assumptions (including whether effects are per 1 unit, per 10 units, or per standard deviation); iii) use consistent and transparent reference standards, ideally with separate reporting for histology-confirmed outcomes versus cytology-only definitions; and iv) prioritize prospective designs that reduce selection bias and support clinically useful prediction modelling.

This systematic review has limitations that should be acknowledged. Substantial heterogeneity was observed for several predictors, partly driven by differences in study design, patient selection, diagnostic reference standards, and variable definitions (including thresholds and units). Although meta-regression and sensitivity analyses explored some sources of variability, residual confounding and selection bias cannot be excluded. Funnel plots suggested possible small-study effects for some predictors, as sex, nodule size, hypoechogenicity, microcalcifications, and TSH, but these assessments are exploratory and can be difficult to interpret in the presence of high heterogeneity and limited numbers of studies for certain predictors. No age restriction was applied during study selection, and a small number of included studies had mixed adult-pediatric populations. As predictors of malignancy may differ by age group, this variation may explain part of the heterogeneity observed for age. Similarly, the discordance between non-significant individual estimates and a significant pooled effect for family history shows that pooling can reveal a true effect by increasing precision, but can also be vulnerable to bias, such as surveillance effects. Despite these limitations, this review also has some strengths that merit discussion. Strengths include PROSPERO registration and PRISMA-compliant reporting, a comprehensive multi-database search, dual independent screening and extraction, structured risk of bias appraisal by study design, and random-effects meta-analyses complemented by moderator and sensitivity analyses. By consolidating commonly reported predictors and quantifying pooled associations where feasible, this review provides a clinician-oriented synthesis and highlights methodological priorities for future studies with more comparable evidence.

This systematic review and meta-analysis support a multifactorial view of malignancy risk in TNs, in which structured ultrasound assessment remains central and is meaningfully complemented by selected patient-level and laboratory variables. Associations for age, nodule size, BMI, and several secondary US features were inconsistent and appeared strongly influenced by diagnostic pathways and study design. Given the heterogeneity across studies, our pooled estimates are best interpreted as evidence of a consistent direction of association, whose magnitude may vary across populations and clinical settings. Future prospective studies using standardized ultrasound lexicons and consistent reference standards are needed to refine these estimates and support clinically useful prediction models.

## Data Availability

The original contributions presented in the study are included in the article/**Supplementary Material**. Further inquiries can be directed to the corresponding author.
