# Correction: Sociodemographic and clinical predictors of malignancy in thyroid nodules: a systematic review and meta-analysis

**DOI:** 10.3389/fendo.2026.1981738

**Published:** 2026-10-05

**Authors:** Vanessa Neto, Catarina Leitão, Cláudia Martins, Margarida Fardilha, Maria Teresa Herdeiro, Alexandra Nunes

**Affiliations:** iBiMED - Institute of Biomedicine, Department of Medical Sciences, University of Aveiro, Aveiro, Portugal

**Keywords:** meta-analysis, risk factors, systematic review, thyroid cancer, thyroid nodules

In the published article, the sentence “No restriction was applied regarding participants’ age.”, which described the age eligibility criteria, was not sufficiently clear.

A correction has been made to the section **Methods**, *Study selection and eligibility criteria*, Paragraph 2:

“Studies enrolling adults, with no restriction on whether the study population consisted exclusively of adults or included both adults and pediatric participants (mixed-age populations), were eligible. Studies restricted exclusively to children and adolescents were excluded.”

The related sentence in the **Discussion** section, “No age restriction was applied during study selection, and a small number of included studies had mixed adult-pediatric populations.”, was revised for consistency.

A correction has been made to the section **Discussion**, Paragraph 11:

“Some of the included studies had mixed adult-pediatric populations. As predictors of malignancy may differ by age group, this variation may explain part of the heterogeneity observed for age.”

The original version of this article has been updated.

