# Supplementary material for: Sociodemographic and clinical predictors of malignancy in thyroid nodules: a systematic review and meta-analysis
Source: Front Endocrinol (Lausanne). 2026 Aug 26;17:1885823. doi: 10.3389/fendo.2026.1885823 (PMC13553268; doi:10.3389/fendo.2026.1885823)
Supplement: Supplementary file 1 [file SupplementaryFile1.docx]

**Supplementary material**

**Text S1.** Full search expression for each database.

**PUBMED – 12270 papers**

(determinant* OR factor* OR demographic) AND thyroid AND (nodule* OR cancer OR carcinoma OR neoplas* OR tumor OR adenoma) AND (progression OR incidence OR risk)

**SCOPUS – 10910 papers**

TITLE-ABS-KEY ((determinant* OR factor* OR demographic) AND thyroid AND (nodule* OR cancer OR carcinoma OR neoplas* OR tumor OR adenoma) AND (progression OR incidence OR risk))

**ISI web of Science – 7935 papers**

ALL FIELDS (determinant* OR factor* OR demographic) AND thyroid AND (nodule* OR cancer OR carcinoma OR neoplas* OR tumor OR adenoma) AND (progression OR incidence OR risk)

**EMBASE – 4244 papers**

(determinant$ OR factor$ OR demographic).af. (thyroid).af. (nodule$ OR cancer OR carcinoma OR neoplas$ OR tumor OR adenoma).af. (progression OR incidence OR risk).af. 1 and 2 and 3 and 4

**Figure 1.** Funnel plots for the meta-analysis of the association between each analysed sociodemographic and clinical factor and malignancy.

**Table 1.** Sociodemographic and clinical risk factors assessed in each of the included studies.

|  | **Sociodemographic**  **risk factors** | | | **Clinical**  **risk factors** | | | | | | | | | | | | | | | | |
| --- | --- | --- | --- | --- | --- | --- | --- | --- | --- | --- | --- | --- | --- | --- | --- | --- | --- | --- | --- | --- |
| **Author and year** | Age | Sex | NFA:  (O) Occupation  (Re) Residence  (Ra) Race  (E) Ethnicity | BMI | Location | Size | Nodule Number | Echogenicity | Composition | Calcifications | Shape | Margins | Vascularity | Halo | Echotexture | TH | TA | Family History of TC | Compressive Symptoms | NFA:  (H/W) Height/Weight  (WC/HC) WC/HC  (BSA) BSA  (TV/TW) TV/TW  (C) Consistency  (M) Mobility |
| **Ageeli, RS - 2022** (1) | ⚫ | ⚫ | - | - | ⚫ | - | - | - | - | - | - | - | - | - | - | - | - | - | - | - |
| **Ajarma, KY - 2020** (2) | ⚫ | ⚫ | - | - | - | ⚫ | ⚫ | - | - | - | - | - | - | - | - | - | - | - | - | - |
| **Al Dawish, MA - 2018** (3) | - | - | - | - | - | ⚫ | - | - | - | - | - | - | - | - | - | ⚫ | - | - | - | - |
| **Al Dawish, MA - 2020** (4) | - | - | - | - | - | - | - | ⚫ | - | ⚫ | - | - | - | - | - | - | - | - | - | - |
| **Alaraifi, AK - 2023** (5) | ⚫ | ⚫ | - | ⚫ | - | ⚫ | - | - | - | - | - | - | - | - | - | ⚫ | - | - | - | - |
| **Al-Bader, A - 2015** (6) | - | - | - | - | - | - | - | - | - | - | - | - | - | - | - | ⚫ | - | - | - | - |
| **Alqahtani, SM - 2024** (7) | ⚫ | ⚫ | - | - | ⚫ | ⚫ | - | ⚫ | ⚫ | ⚫ | ⚫ | ⚫ | ⚫ | ⚫ | ⚫ | - | - | - | - | - |
| **Angell, TE - 2017** (8) | - | - | - | - | - | ⚫ | - | - | - | - | - | - | - | - | - | - | - | - | - | - |
| **Angell, TE - 2019** (9) | ⚫ | ⚫ | - | - | - | ⚫ | ⚫ | - | ⚫ | - | - | - | - | - | - | - | - | - | - | - |
| **Apostolou, K - 2021** (10) | ⚫ | ⚫ | - | - | - | ⚫ | - | - | - | - | - | - | - | - | - | - | - | - | - | - |
| **Arduc, A - 2015** (11) | ⚫ | - | - | ⚫ | - | ⚫ | - | ⚫ | - | ⚫ | ⚫ | - | ⚫ | - | - | ⚫ | - | - | - | ⚫**^WC/HC^** |
| **Aydin, Y - 2014** (12) | - | - | - | - | - | - | - | ⚫ | - | - | - | - | - | - | - | - | - | - | - | - |
| **Babayid, Y - 2024** (13) | ⚫ | ⚫ | - | - | ⚫ | ⚫ | ⚫ | ⚫ | ⚫ | ⚫ | ⚫ | ⚫ | - | - | - | - | - | - | - | - |
| **Baser, H - 2016** (14) | - | - | - | - | - | ⚫ | - | - | - | - | - | - | - | - | - | ⚫ | ⚫ | - | - | - |
| **Batawil, N - 2014** (15) | ⚫ | ⚫ | - | - | - | ⚫ | - | ⚫ | ⚫ | ⚫ | - | ⚫ | - | - | - | - | - | - | - | - |
| **Behbahaninia, M - 2022** (16) | - | - | - | - | - | ⚫ | - | ⚫ | ⚫ | ⚫ | ⚫ | ⚫ | - | - | - | - | - | - | - | - |
| **Boonrod, A - 2021** (17) | ⚫ | ⚫ | - | - | - | ⚫ | - | - | - | - | ⚫ | - | - | - | - | ⚫ | - | - | - | - |
| **Brophy, C - 2016** (18) | - | - | - | - | - | - | - | - | - | ⚫ | - | ⚫ | - | - | - | - | - | - | - | - |
| **Bukasa Kakamba, J - 2021** (19) | ⚫ | - | - | - | - | ⚫ | - | ⚫ | ⚫ | ⚫ | - | - | - | - | - | - | - | ⚫ | - | - |
| **Campbell, MJ - 2015** (20) | ⚫ | ⚫ | - | - | ⚫ | - | - | - | - | - | - | - | - | - | - | - | - | ⚫ | ⚫ | - |
| **Canete, EJ - 2014** (21) | - | ⚫ | - | - |  | ⚫ | - | ⚫ | ⚫ | ⚫ | - | ⚫ | - | - | - | - | - | - | ⚫ | ⚫**^C+M^** |
| **Cao, SL - 2024** (22) | ⚫ | ⚫ | - | - | ⚫ | ⚫ | - | - | - | ⚫ | ⚫ | ⚫ | - | - | - | - | - | - | - | - |
| **Cappelli, C - 2019** (23) | ⚫ | ⚫ | - | - | - | - | - | ⚫ | - | ⚫ | - | ⚫ | ⚫ | - | - | - | - | - | - | - |
| **Castagna, MG - 2014** (24) | - | - | - | - | - | - | ⚫ | - | - | - | - | - | - | - | - | ⚫ | - | - | - | - |
| **Cavallo, A – 2017** (25) | - | - | - | - | - | ⚫ | - | - | - | - | - | - | - | - | - | - | - | - | - | - |
| **Chen, WH – 2022** (26) | ⚫ | - | - | - | - | ⚫ | ⚫ | - | - | - | - | - | - | - | - | - | - | - | - | ⚫**^TV/TW^** |
| **Chen, Z – 2022** (27) | ⚫ | ⚫ | - | - | - | ⚫ | - | - | - | - | - | - | - | - | - | ⚫ | ⚫ | - | - | - |
| **Chen, X – 2023** (28) | - | - | - | - | - | ⚫ | - | - | - | ⚫ | - | - | - | - | - | - | - | - | - | - |
| **Chiefari, E – 2024** (29) | - | ⚫ | - | - | - | - | - | - | - | - | - | - | - | - | - | - | - | - | - | - |
| **Chiofalo, MG – 2018** (30) | ⚫ | ⚫ | - | - | - | ⚫ | - | - | - | - | - | - | - | - | - | - | - | - | - | ⚫**^C^** |
| **Cho, YY - 2021** (31) | ⚫ | ⚫ | - | ⚫ | - | ⚫ | ⚫ | - | - | - | - | - | - | - | - | ⚫ | - | - | - | - |
| **Choi, JS – 2015** (32) | ⚫ | ⚫ | - | - | - | ⚫ | - | - | - | - | - | - | - | - | - | ⚫ | - | - | - | - |
| **Cozzolino, A – 2020** (33) | ⚫ | ⚫ | - | - | ⚫ | ⚫ | - | ⚫ | - | ⚫ | ⚫ | ⚫ | ⚫ | ⚫ | - | - | - | ⚫ | - | - |
| **Crudele, L – 2023** (34) | - | - | - | ⚫ | - | - | - | - | - | - | - | - | - | - | - | - | - | - | - | ⚫**^WC/HC^** |
| **Delman, AM – 2023** (35) | ⚫ | ⚫ | - | - | - | - | - | - | - | - | - | - | - | - | - | - | - | - | - | - |
| **Deng, XH – 2017** (36) | ⚫ | - | - | - | - | ⚫ | - | ⚫ | - | ⚫ | - | ⚫ | - | - | - | - | - | - | - | - |
| **Deng, Y – 2022** (37) | ⚫ | ⚫ | - | - | - | ⚫ | ⚫ | - | - | - | - | - | - | - | - | ⚫ | - | - | - | - |
| **Ding, J – 2019** (38) | ⚫ | ⚫ | - | - | - | - | - | - | - | ⚫ | ⚫ | ⚫ | - | - | - | - | - | - | - | - |
| **Dobruch-Sobczak, K – 2019** (39) | - | - | - | - | - | - | - | ⚫ | ⚫ | ⚫ | ⚫ | ⚫ | - | - | - | - | - | - | - | - |
| **Dong, S – 2021** (40) | ⚫ | - | - | - | - | ⚫ | - | - | ⚫ | ⚫ | - | - | - | - | - | ⚫ | ⚫ | - | - | - |
| **Du, J – 2023** (41) | ⚫ | ⚫ | - | - | - | - | - | ⚫ | ⚫ | ⚫ | ⚫ | ⚫ | - | - | - | ⚫ | ⚫ | - | - | - |
| **Ebrahim, H – 2023** (42) | ⚫ | ⚫ | - | - | - | ⚫ | ⚫ | - | - | - | - | - | - | - | - | - | - | - | - | - |
| **Egset, AV – 2017** (43) | - | - | - | - | - | - | - | - | - | - | - | - | - | - | - | ⚫ | - | - | - | - |
| **Eisa, N – 2018** (44) | ⚫ | ⚫ | - | - | - | ⚫ | - | - | - | - | - | - | - | - | - | - | - | ⚫ | - | - |
| **Eissa, MS – 2020** (45) | - | - | - | ⚫ | - | - | - | - | - | - | - | - | - | - | - | - | - | - | - | ⚫**^WC/HC^** |
| **El Sayed Ibrahim, MM – 2019** (46) | ⚫ | ⚫ | - | - | - | - | - | ⚫ | - | ⚫ | ⚫ | - | ⚫ | - | - | - | - | - | - | - |
| **Elbalka, SS – 2021** (47) | - | ⚫ | - | - | - | ⚫ | - | - | - | - | - | - | - | - | - | - | - | - | - | - |
| **Espinosa De Ycaza, AE – 2016** (48) | ⚫ | ⚫ | - | - | - | ⚫ | - | ⚫ | ⚫ | ⚫ | - | - | ⚫ | - | - | ⚫ | - | ⚫ | - | - |
| **Fernández-Trujillo, C – 2020** (49) | ⚫ | - | - | - | - | - | - | - | - | - | - | - | - | - | - | ⚫ | - | - | - | - |
| **Fighera, TM – 2015** (50) | ⚫ | ⚫ | - | - | - | - | ⚫ | - | ⚫ | - | - | - | - | - | - | ⚫ | - | - | - | - |
| **Gallegos-Vargas, JA – 2016** (51) | ⚫ | ⚫ | - | - | - | - | - | - | - | - | - | - | - | - | - | - | - | - | - | - |
| **Garino, F – 2015** (52) | - | - | - | - | - | - | - | ⚫ | - | ⚫ | - | ⚫ | ⚫ | - | - | - | - | - | - | - |
| **Gezer, E – 2022** (53) | ⚫ | ⚫ | - | - | ⚫ | - | - | ⚫ | - | - | - | - | - | - | - | - | - | - | - | - |
| **Grani, G – 2014** (54) | ⚫ | ⚫ | - | - | - | ⚫ | ⚫ | - | ⚫ | ⚫ | - | - | - | - | - | ⚫ | ⚫ | - | - | - |
| **Grani, G – 2023** (55) | - | - | - | - | - | - | - | ⚫ | ⚫ | ⚫ | ⚫ | ⚫ | - | - | - | - | - | - | - | - |
| **Gu, WJ – 2015** (56) | - | - | - | - | - | - | ⚫ | ⚫ | ⚫ | ⚫ | ⚫ | ⚫ | ⚫ | - | - | - | - | - | - | - |
| **Guarnotta, V – 2023** (57) | - | - | - | - | - | ⚫ | - | ⚫ | - | ⚫ | - | ⚫ | ⚫ | ⚫ | - | ⚫ | - | - | - | - |
| **Gweon, HM – 2017** (58) | ⚫ | - | - | - | - | - | - | - | - | ⚫ | ⚫ | ⚫ | - | - | - | - | - | - | - | - |
| **Hacim, NA – 2021** (59) | - | - | - | - | - | - | - | ⚫ | ⚫ | ⚫ | ⚫ | ⚫ | ⚫ | ⚫ | - | - | - | - | - | - |
| **Handelsman, RS – 2019** (60) | - | ⚫ | - | - | - | - | - | - | - | - | - | - | - | - | - | - | - | - | - | - |
| **Hong, IK – 2016** (61) | - | - | - | - | - | - | - | - | - | - | - | ⚫ | - | - | - | - | - | - | - | - |
| **Hong, SH – 2018** (62) | - | - | - | - | - | ⚫ | - | ⚫ | - | ⚫ | ⚫ | ⚫ | - | - | - | - | - | - | - | - |
| **Hou, CJ – 2018** (63) | - | - | - | - | - | - | - | ⚫ | - | ⚫ | ⚫ | ⚫ | - | - | - | - | - | - | - | - |
| **Huan, Q – 2014** (64) | ⚫ | - | ⚫**^O^** | - | - | ⚫ | - | ⚫ | ⚫ | ⚫ | ⚫ | ⚫ | ⚫ | - | - | - | ⚫ | - | - | - |
| **Huang, J – 2020** (65) | ⚫ | ⚫ | ⚫**^Re^** | ⚫ | - | - | - | - | - | - | - | - | - | - | - | - | - | - | - | ⚫**^BSA^** |
| **Huang, J – 2021** (66) | - | - | - | - | - | ⚫ | - | ⚫ | - | ⚫ | ⚫ | ⚫ | - | - | - | - | ⚫ | - | - | - |
| **Hwang, SH – 2016** (67) | ⚫ | ⚫ | - | - | - | - | ⚫ | - | - | - | - | - | - | - | - | ⚫ | - | ⚫ | - | - |
| **Ito, Y – 2016** (68) | ⚫ | ⚫ | - | - | - | ⚫ | - | - | - | - | - | - | - | - | - | ⚫ | - | - | - | - |
| **Jabar, ASS – 2019** (69) | - | - | - | - | - | - | - | ⚫ | ⚫ | ⚫ | ⚫ | ⚫ | - | - | - | - | - | - | - | - |
| **Jasim, S – 2020** (70) | ⚫ | ⚫ | - | - | ⚫ | ⚫ | - | - | - | - | - | - | - | - | - | - | - | ⚫ | - | - |
| **Jia, X – 2020** (71) | ⚫ | ⚫ | - | - | - | ⚫ | - | - | - | - | - | - | - | - | - | ⚫ | ⚫ | - | - | - |
| **Kaliszewski, K – 2018** (72) | ⚫ | ⚫ | - | - | - | ⚫ | - | ⚫ | - | ⚫ | ⚫ | ⚫ | ⚫ | - | - | - | - | - | - | - |
| **Kaliszewski, K – 2021** (73) | ⚫ | ⚫ | - | - | - | - | - | ⚫ | - | ⚫ | ⚫ | ⚫ | ⚫ | - | - | - | - | - | - | - |
| **Kaliszewski, K – 2021** (74) | ⚫ | - | - | - | - | ⚫ | - | ⚫ | - | ⚫ | ⚫ | ⚫ | ⚫ | - | - | - | - | - | - | - |
| **Kaliszewski, K – 2022** (75) | ⚫ | ⚫ | - | - | - | - | - | ⚫ | - | ⚫ | ⚫ | ⚫ | ⚫ | - | - | ⚫ | - | - | - | - |
| **Karabacak, U – 2022** (76) | - | - | - | - | - | - | - | - | ⚫ | ⚫ | - | - | - | - | - | - | - | - | - | - |
| **Khodabandelu, S – 2022** (77) | ⚫ | ⚫ | - | - | ⚫ | ⚫ | - | ⚫ | ⚫ | ⚫ | ⚫ | ⚫ | ⚫ | - | - | - | - | - | - | - |
| **Kim, GR – 2016** (78) | ⚫ | ⚫ | - | - | - | ⚫ | - | ⚫ | - | ⚫ | - | - | ⚫ | - | ⚫ | - | - | - | - | - |
| **Kim, HJ – 2017** (79) | ⚫ | ⚫ | - | - | - | ⚫ | - | - | - | - | - | - | - | - | - | ⚫ | - | - | - | - |
| **Kim, HJ – 2017** (80) | ⚫ | ⚫ | - | ⚫ | - | - | - | - | - | - | - | - | - | - | - | ⚫ | ⚫ | - | - | - |
| **Kim, K – 2021** (81) | ⚫ | ⚫ | - | - | - | - | - | - | - | - | - | - | - | - | - | - | - | - | - | - |
| **Kişioğlu, SV – 2022** (82) | - | - | - | - | - | - | - | ⚫ | - | ⚫ | - | ⚫ | ⚫ | - | - | - | - | - | - | - |
| **Kong, J – 2017** (83) | - | - | - | - | - | - | - | - | - | ⚫ | ⚫ | - | ⚫ | - | - | - | - | - | - | - |
| **Kornelius, E – 2020** (84) | ⚫ | ⚫ | - | - | - | ⚫ | ⚫ | - | - | - | - | - | - | - | - | - | - | - | - | - |
| **Kotecka-Blicharz, A – 2022** (85) | - | - | - | - | - | - | - | ⚫ | - | ⚫ | ⚫ | ⚫ | - | - | - | - | - | - | - | - |
| **Krátký, J – 2018** (86) | - | - | - | - | - | - | - | - | - | - | - | - | - | - | - | ⚫ | ⚫ | - | - | ⚫**^TV/TW^** |
| **Kuo, TC – 2020** (87) | - | - | - | - | - | - | - | - | - | ⚫ | - | ⚫ | - | - | - | - | - | - | - | - |
| **Kuru, B – 2016** (88) | - | - | - | - | - | - | - | ⚫ | ⚫ | ⚫ | - | ⚫ | ⚫ | - | - | - | - | - | - | - |
| **Kuru, B – 2018** (89) | - | - | - | - | - | ⚫ | - | ⚫ | ⚫ | ⚫ | - | - | ⚫ | - | - | - | - | - | - | - |
| **Lee, KH – 2014** (90) | ⚫ | - | - | - | - | ⚫ | - | ⚫ | - | ⚫ | ⚫ | ⚫ | - | - | - | - | - | - | - | - |
| **Li, T – 2015** (91) | - | ⚫ | - | - | ⚫ | ⚫ | ⚫ | - | - | - | - | ⚫ | - | - | ⚫ | ⚫ | ⚫ | - | - | - |
| **Li, W – 2021** (92) | - | - | - | - | - | - | - | ⚫ | ⚫ | ⚫ | - | - | - | ⚫ | - | - | - | - | - | - |
| **Li, W – 2024** (93) | - | - | - | - | - | - | - | - | ⚫ | ⚫ | ⚫ | - | - | - | - | - | - | - | - | - |
| **Liu, Y – 2016** (94) | - | - | - | - | - | - | - | - | - | ⚫ | ⚫ | - | - | - | - | - | - | - | - | - |
| **Liu, J – 2018** (95) | ⚫ | - | - | - | - | ⚫ | - | ⚫ | ⚫ | ⚫ | ⚫ | ⚫ | ⚫ | - | - | ⚫ | ⚫ | - | - | - |
| **Lourdes Ng, J – 2023** (96) | ⚫ | ⚫ | - | - | - | ⚫ | - | ⚫ | - | ⚫ | - | - | ⚫ | - | - | - | - | ⚫ | ⚫ | - |
| **Lynch, CA – 2022** (97) | ⚫ | ⚫ | ⚫**^Ra^** | - | - | ⚫ | - | - | - | - | - | - | - | - | - | - | - | ⚫ | - | - |
| **Macias, CA – 2015** (98) | ⚫ | - | - | - | - | ⚫ | - | - | - | ⚫ | - | - | - | - | - | - | - | - | - | - |
| **Maeda, H – 2016** (99) | - | - | - | - | - | - | - | - | - | - | - | - | - | - | - | ⚫ | - | - | - | - |
| **Mazzone, M – 2021** (100) | ⚫ | ⚫ | ⚫**^Re^** | ⚫ | - | - | ⚫ | - | - | - | - | - | - | - | - | - | - | - | - | - |
| **Melak, T – 2014** (101) | ⚫ | ⚫ | - | - | - | ⚫ | ⚫ | - | - | - | - | - | - | - | - | - | - | - | - | - |
| **Mileva, M – 2018** (102) | ⚫ | - | - | - | - | ⚫ | - | ⚫ | - | ⚫ | - | - | ⚫ | - | - | - | - | - | - | - |
| **Mohamed, TZ – 2022** (103) | - | ⚫ | - | - | - | - | - | - | - | - | - | - | - | - | - | - | - | - | - | - |
| **Molina-Vega, M – 2019** (104) | ⚫ | ⚫ | - | - | - | ⚫ | - | ⚫ | ⚫ | ⚫ | - | ⚫ | - | - | - | - | - | - | - | - |
| **Moosa, NA – 2021** (105) | ⚫ | ⚫ | - | - | - | ⚫ | ⚫ | - | ⚫ | ⚫ | - | - | ⚫ | - | - | - | - | ⚫ | ⚫ | - |
| **Mu, N – 2018** (106) | ⚫ | ⚫ | - | - | - | ⚫ | - | - | - | - | - | - | - | - | - | - | - | - | - | - |
| **Muhanhali, D – 2024** (107) | - | - | - | - | - | - | - | - | - | - | - | - | - | - | - | ⚫ | - | - | - | - |
| **Naidu, K – 2023** (108) | - | - | - | - | - | ⚫ | - | ⚫ | - | ⚫ | ⚫ | ⚫ | ⚫ | - | - | - | - | - | - | - |
| **Nawarathna, NJ – 2018** (109) | ⚫ | ⚫ | - | - | - | - | - | - | - | - | - | - | - | - | - | - | - | - | - | - |
| **Nguyen, BQ – 2023** (110) | ⚫ | ⚫ | - | - | - | ⚫ | - | ⚫ | ⚫ | ⚫ | ⚫ | ⚫ | - | - | - | - | - | - | - | - |
| **Nitipir, C – 2018** (111) | ⚫ | ⚫ | - | - | - | - | - | - | - | - | - | - | - | - | - | - | - | - | - | - |
| **Nonchev, BI – 2017** (112) | - | - | - | - | - | - | - | ⚫ | ⚫ | ⚫ | ⚫ | ⚫ | - | - | - | - | - | - | - | - |
| **Öcal, B – 2019** (113) | ⚫ | - | - | - | - | - | ⚫ | - | - | ⚫ | - | ⚫ | - | - | - | - | - | - | - | - |
| **Ou, D – 2020** (114) | - | - | - | - | - | ⚫ | - | - | ⚫ | ⚫ | ⚫ | ⚫ | ⚫ | ⚫ | ⚫ | - | - | - | - | - |
| **Öz, B – 2018** (115) | ⚫ | - | - | - | - | - | ⚫ | - | - | ⚫ | - | - | - | - | - | - | - | - | - | - |
| **Özdemi̇r, A – 2022** (116) | - | - | - | - | - | - | - | ⚫ | ⚫ | - | - | - | - | - | - | - | - | - | - | - |
| **Pak, K – 2017** (117) | ⚫ | ⚫ | - | ⚫ | - | - | - | - | - | - | - | - | - | - | - | - | - | - | - | ⚫**^H/W^** |
| **Pappa, T – 2022** (118) | ⚫ | ⚫ | - | - | - | ⚫ | - | - | - | - | ⚫ | - | - | - | - | - | - | - | - | - |
| **Peng, X – 2020** (119) | ⚫ | - | - | - | - | ⚫ | - | - | - | - | - | - | - | - | - | - | ⚫ | - | - | - |
| **Peng, B – 2024** (120) | ⚫ | - | - | - | - | ⚫ | - | ⚫ | - | ⚫ | - | - | - | - | - | - | - | - | - | - |
| **Petric, R – 2014** (121) | - | ⚫ | - | - | - | - | - | - | - | - | - | - | - | - | - | ⚫ | - | - | - | - |
| **Pikis, G – 2021** (122) | ⚫ | ⚫ | - | ⚫ | - | ⚫ | ⚫ | - | - | ⚫ | ⚫ | ⚫ | ⚫ | - | - | - | - | - | - | ⚫**^C^** |
| **Pompili, GG – 2018** (123) | - | - | - | - | - | - | ⚫ | ⚫ | ⚫ | ⚫ | - | ⚫ | ⚫ | - | - | - | - | - | - | - |
| **Qureshi, K – 2022** (124) | - | - | - | - | - | - | - | - | - | - | - | - | - | - | - | ⚫ | ⚫ | - | - | - |
| **Rago, T – 2014** (125) | ⚫ | ⚫ | - | - | - | ⚫ | - | - | - | - | - | - | - | - | - | - | - | - | - | - |
| **Ramundo, V – 2019** (126) | - | - | - | - | ⚫ | - | - | - | ⚫ | - | - | - | - | - | - | - | - | - | - | - |
| **Ren, T – 2023** (127) | - | - | - | - | - | - | - | ⚫ | - | ⚫ | ⚫ | ⚫ | - | - | - | - | - | - | - | - |
| **Rianto, BUD – 2019** (128) | - | - | - | - | - | - | - | - | - | - | - | - | - | - | - | ⚫ | - | - | - | - |
| **Rios, A – 2016** (129) | - | - | - | - | - | - | - | ⚫ | - | ⚫ | - | - | ⚫ | - | - | - | - | - | - | - |
| **Rosenblum, RC – 2019** (130) | - | ⚫ | - | - | - | - | - | - | - | - | - | - | - | - | - | - | - | - | - | - |
| **Ryu, YJ – 2014** (131) | ⚫ | - | - | - | - | ⚫ | - | - | - | - | - | - | - | - | - | - | - | - | - | - |
| **Şah Ünal, FT – 2024** (132) | ⚫ | ⚫ | - | - | ⚫ | ⚫ | - | - | - | - | - | - | - | - | ⚫ | ⚫ | - | - | - | - |
| **Sahib, AM – 2023** (133) | - | ⚫ | - | - | - | - | - | - | - | - | - | - | - | - | - | - | - | - | - | - |
| **Sasson, M – 2017** (134) | - |  | - | - | - | - | - | - | - | - | - | - | - | - | - | ⚫ | - | - | - | - |
| **Seo, JW – 2017** (135) | ⚫ | ⚫ | - | - | - | ⚫ | - | - | - | - | - | - | - | - | - | - | - | - | - | - |
| **Shayganfar, A – 2020** (136) | - | - | - | - | - | ⚫ | - | - | - | - | - | - | - | - | - | - | - | - | - | - |
| **Shin, TJ – 2020** (137) | ⚫ | ⚫ | ⚫**^Ra^** | - | - | ⚫ | - | - | - | - | - | - | - | - | - | - | - | - | - | - |
| **Slijepcevic, N – 2015** (138) | ⚫ | ⚫ | - | - | - | - | ⚫ | - | - | - | - | - | - | - | - | - | - | - | - | - |
| **Słowińska-Klencka, D – 2020** (139) | - | - | - | - | - | - | - | ⚫ | ⚫ | ⚫ | ⚫ | ⚫ | ⚫ | - | - | - | - | - | - | - |
| **Sohn, SY – 2014** (140) | ⚫ | ⚫ | - | - | - | - | ⚫ | - | - | - | - | - | - | - | - | ⚫ | - | - | - | - |
| **Song, Q – 2021** (141) | - | - | - | - | - | - | - | - | - | ⚫ | - | ⚫ | - | - | - | - | - | - | - | - |
| **Sun, J – 2023** (142) | - | - | - | - | - | - | - | - | - | - | - | - | - | - | - | ⚫ | - | - | - | - |
| **Sutton, W – 2021** (143) | ⚫ | ⚫ | ⚫**^Ra^** | - | - | ⚫ | ⚫ | - | - | - | - | - | - | - | - | - | - | ⚫ | ⚫ | - |
| **Swan, KZ – 2018** (144) | - | - | - | - | - | - | ⚫ | - | - | - | - | - | - | - | - | ⚫ | - | - | - | - |
| **Talmor, G – 2022** (145) | ⚫ | - | - | - | - | - | - | - | - | ⚫ | - | - | - | - | - | - | - | - | - | - |
| **Tam, AA – 2020** (146) | ⚫ | ⚫ | - | - | - | - | - | - | - | - | - | - | - | - | - | - | ⚫ | - | - | - |
| **Tang, Q – 2023** (147) | - | - | - | - | - | ⚫ | - | - | - | - | - | ⚫ | - | - | - | - | - | - | - | - |
| **Teklewold, B – 2022** (148) | ⚫ | ⚫ | - | - | - | ⚫ | ⚫ | - | - | - | - | - | - | - | - | - | - | - | ⚫ | ⚫**^C^** |
| **Trimboli, P – 2018** (149) | - | - | - | - | - | ⚫ | - | - | - | - | - | - | - | - | - | - | - | - | - | - |
| **Turkyilmaz, S – 2017** (150) | ⚫ | ⚫ | - | - | - | ⚫ | ⚫ | ⚫ | ⚫ | ⚫ | - | - | - | ⚫ | - | ⚫ | - | - | - | - |
| **Tutuncu, Y – 2014** (151) | - | - | - | - | - | - | - | ⚫ | ⚫ | ⚫ | - | - | ⚫ | - | - | - | - | - | - | - |
| **Vasileiadis, I – 2014** (152) | ⚫ | ⚫ | - | - | - | ⚫ | - | - | - | - | - | - | - | - | - | ⚫ | ⚫ | - | - | ⚫**^TV/TW^** |
| **Wang, L – 2015** (153) | - | - | - | - | - | - | - | - | - | - | - | - | - | - | - | ⚫ | ⚫ | - | - | - |
| **Wang, H – 2018** (154) | - | - | - | - | - | - | - | - | - | - | ⚫ | - | - | - | - | - | - | - | - | - |
| **Wang, Z – 2020** (155) | - | - | - | - | - | - | - | ⚫ | - | ⚫ | ⚫ | ⚫ | - | - | - | - | - | - | - | - |
| **Wang, Y – 2021** (156) | - | - | - | - | - | - | - | - | - | ⚫ | - | ⚫ | - | - | - | - | - | - | - | - |
| **Wang, G – 2022** (157) | ⚫ | - | - | - | - | ⚫ | - | ⚫ | - | ⚫ | ⚫ | ⚫ | - | - | - | - | - | - | - | - |
| **Witczak, J – 2016** (158) | ⚫ | ⚫ | - | - | - | ⚫ | ⚫ | ⚫ | ⚫ | ⚫ | - | ⚫ | - | - | - | ⚫ | - | - | - | - |
| **Wong, SL – 2015** (159) | - | - | - | - | - | - | - | - | - | - | - | - | - | - | - | - | ⚫ | - | - | - |
| **Wong, MW – 2017** (160) | ⚫ | ⚫ | - | - | - | - | - | - | - | - | - | - | - | - | - | - | - | - | - | ⚫**^TV/TW^** |
| **Wu, X – 2014** (161) | ⚫ | ⚫ | - | - | - | - | - | - | - | - | - | - | - | - | - | ⚫ | ⚫ | - | - | - |
| **Wu, SJ – 2022** (162) | - | - | - | - | - | - | ⚫ | - | - | - | - | - | ⚫ | ⚫ | ⚫ | - | - | - | - | - |
| **Xi, X – 2021** (163) | - | - | - | - | - | - | - | ⚫ | ⚫ | ⚫ | - | ⚫ | - | - | - | - | - | - | - | - |
| **Xin, Y – 2021** (164) | - | - | - | - | - | - | - | ⚫ | ⚫ | ⚫ | ⚫ | ⚫ | ⚫ | - | - | - | - | - | - | - |
| **Xu, SY – 2015** (165) | - | - | - | - | - | - | - | ⚫ | ⚫ | ⚫ | ⚫ | ⚫ | ⚫ | - | - | - | - | - | - | - |
| **Xu, DH – 2018** (166) | - | - | - | ⚫ | - | - | - | - | - | - | - | - | - | - | - | - | - | - | - | - |
| **Xu, S – 2022** (167) | ⚫ | ⚫ | - | - | - | - | - | - | - | ⚫ | ⚫ | ⚫ | ⚫ | - | - | - | - | - | - | - |
| **Xu, N – 2022** (168) | ⚫ | ⚫ | - | ⚫ | - | - | - | - | - | - | - | - | - | - | - | - | ⚫ | - | - | - |
| **Yan, HX – 2017** (169) | ⚫ | ⚫ | - | ⚫ | - | - | ⚫ | - | - | - | - | - | - | - | - | ⚫ | - | - | - | - |
| **Yang, TT – 2016** (170) | - | - | - | - | - | ⚫ | - | - | - | - | - | - | - | - | - | - | - | - | - | - |
| **Yazdani-Charati, J – 2018** (171) | - | - | - | - | ⚫ | - | ⚫ | - | - | - | - | - | - | - | - | ⚫ | - | - | - | - |
| **Yazgan, A – 2016** (172) | - | - | - | - | - | - | - | ⚫ | - | - | - | - | - | - | - | - | - | - | - | - |
| **Yazici, P – 2016** (173) | ⚫ | ⚫ | - | - | - | - | - | - | - | - | - | - | - | - | - | ⚫ | ⚫ | - | - | - |
| **Yeon, EK – 2020** (174) | ⚫ | - | - | - | - | - | - | ⚫ | ⚫ | ⚫ | ⚫ | ⚫ | - | - | - | - | - | - | - | - |
| **Yi, D – 2022** (175) | - | - | - | - | - | - | - | - | - | - | ⚫ | ⚫ | - | - | - | - | - | - | - | - |
| **Yoo, MR – 2015** (176) | - | - | - | - | - | - | - | - | ⚫ | - | ⚫ | ⚫ | - | - | - | - | - | - | - | - |
| **Yoon, JH – 2014** (177) | ⚫ | - | - | - | - | ⚫ | - | ⚫ | - | ⚫ | ⚫ | ⚫ | - | - | - | - | - | - | - | - |
| **Yu, Z – 2021** (178) | ⚫ | ⚫ | - | - | - | - | - | - | - | - | - | - | - | - | - | - | - | - | - | - |
| **Yu, B – 2022** (179) | ⚫ | ⚫ | - | - | - | ⚫ | - | ⚫ | ⚫ | - | ⚫ | ⚫ | - | - | - | - | - | - | - | - |
| **Zeng, R – 2016** (180) | ⚫ | ⚫ | ⚫**^Re+E^** | ⚫ |  | ⚫ | - | - | - | - | - | - | - | - | - | ⚫ | ⚫ | - | - | - |
| **Zhang, YF – 2014** (181) | - | ⚫ | - | - | - | - | - | ⚫ | - | - | ⚫ | - | - | - | - | - | - | - | - | - |
| **Zhang, H – 2014** (182) | - | - | - | - | - | - | - | ⚫ | - | - | ⚫ | ⚫ | - | - | - | - | - | - | - | - |
| **Zhang, J – 2015** (183) | ⚫ | ⚫ | - | - | - | ⚫ | ⚫ | ⚫ | ⚫ | ⚫ | ⚫ | ⚫ | ⚫ | ⚫ | - | - | - | - | - | - |
| **Zhang, X – 2018** (184) | - | - | - | - | - | - | - | - | - | - | - | - | - | - | - | ⚫ | ⚫ | - | - | - |
| **Zhang, F – 2019** (185) | ⚫ | ⚫ | ⚫**^E^** | ⚫ | ⚫ | - | ⚫ | - | - | - | ⚫ | - | - | - | - | - | - | - | - | - |
| **Zhang, X – 2022** (186) | ⚫ | - | - | - | - | ⚫ | - | - | - | - | - | - | - | - | - | ⚫ | - | - | - | - |
| **Zhang, F – 2024** (187) | - | - | - | - | - | - | - | ⚫ | ⚫ | ⚫ | - | ⚫ | ⚫ | ⚫ | - | - | - | - | - | - |
| **Zhao, RN – 2015** (188) | ⚫ | - | - | - | - | - | - | - | - | - | ⚫ | - | ⚫ | - | - | - | - | - | - | - |
| **Zhao, H – 2018** (189) | **⚫** | - | - | - | - | ⚫ | ⚫ | - | - | - | - | - | - | - | - | ⚫ | ⚫ | - | - | - |
| **Zhao, S – 2019** (190) | **⚫** | **⚫** | - | ⚫ | - | ⚫ | - | - | - | - | - | - | - | - | - | ⚫ | - | - | - | - |
| **Zhao, RN – 2019** (191) | ⚫ | - | - | - | - | - | - | ⚫ | ⚫ | ⚫ | ⚫ | ⚫ | ⚫ | ⚫ | - | - | - | - | - | - |
| **Zhao, Z – 2022** (192) | ⚫ | ⚫ | - | - | - | - | - | - | - | - | - | - | - | - | - | ⚫ | - | - | - | - |
| **Zivic, R – 2016** (193) | - | - | - | - | - | - | - | - | - | - | - | - | - | - | - | ⚫ | - | - | - | - |
| **Zoric, GV – 2020** (194) | ⚫ | ⚫ | - | - | - | ⚫ | ⚫ | ⚫ | - | ⚫ | - | - | ⚫ | - | - | ⚫ | ⚫ | - | - | - |
| **Total** | **108** | **92** | **1-3-3-2** | **16** | **14** | **87** | **35** | **70** | **46** | **85** | **56** | **66** | **41** | **11** | **6** | **54** | **25** | **11** | **6** | **1-3-1-4-4-1** |
| BMI, body mass index, BSA – body surface area, C - consistency, E- ethnicity, H – height, HC – hip circumference, M – mobility, NFA – not further assessed, O – occupation, Ra – race, Re – residence, TA – thyroid antibodies, TC – thyroid cancer, TH – thyroid hormones, TV – thyroid volume, TW – thyroid weight, W – weight, WC – waist circumference | | | | | | | | | | | | | | | | | | | | |

**Figure 2.** Forest plots of individual and pooled estimates for the association between age and malignancy, stratified by diagnostic method.

**Table 2**. Leave-one-out analyses for sex.

| **estimate** | **se** | **zval** | **pval** | **ci.lb** | **ci.ub** | **Q** | **Qp** | **tau2** | **I2** | **H2** |
| --- | --- | --- | --- | --- | --- | --- | --- | --- | --- | --- |
| **0.2889** | 0.1054 | 2.7410 | 0.0061 | 0.0823 | 0.4955 | 88.9335 | 0.0000 | 0.1845 | 77.4227 | 4.4292 |
| **0.2910** | 0.1077 | 2.7021 | 0.0069 | 0.0799 | 0.5021 | 85.0712 | 0.0000 | 0.1927 | 77.5988 | 4.4640 |
| **0.3360** | 0.1108 | 3.0325 | 0.0024 | 0.1188 | 0.5531 | 93.6647 | 0.0000 | 0.2105 | 79.3915 | 4.8524 |
| **0.2951** | 0.1061 | 2.7809 | 0.0054 | 0.0871 | 0.5031 | 91.1612 | 0.0000 | 0.1911 | 78.1671 | 4.5803 |
| **0.3305** | 0.1136 | 2.9094 | 0.0036 | 0.1079 | 0.5532 | 93.5301 | 0.0000 | 0.2223 | 75.7328 | 4.1208 |
| **0.2981** | 0.1054 | 2.8291 | 0.0047 | 0.0916 | 0.5046 | 91.0686 | 0.0000 | 0.1907 | 78.2204 | 4.5914 |
| **0.3004** | 0.1085 | 2.7677 | 0.0056 | 0.0877 | 0.5131 | 92.1427 | 0.0000 | 0.2014 | 78.9586 | 4.7525 |
| **0.3383** | 0.1110 | 3.0478 | 0.0023 | 0.1207 | 0.5558 | 92.5728 | 0.0000 | 0.2098 | 78.8821 | 4.7353 |
| **0.2859** | 0.1026 | 2.7863 | 0.0053 | 0.0848 | 0.4870 | 88.0891 | 0.0000 | 0.1744 | 76.6041 | 4.2743 |
| **0.3252** | 0.1073 | 3.0301 | 0.0024 | 0.1149 | 0.5356 | 94.2426 | 0.0000 | 0.2024 | 79.2432 | 4.8177 |
| **0.2934** | 0.1042 | 2.8150 | 0.0049 | 0.0891 | 0.4977 | 89.6198 | 0.0000 | 0.1845 | 77.6475 | 4.4738 |
| **0.3364** | 0.1101 | 3.0553 | 0.0022 | 0.1206 | 0.5522 | 93.6142 | 0.0000 | 0.2079 | 79.3303 | 4.8380 |
| **0.3084** | 0.1127 | 2.7363 | 0.0062 | 0.0875 | 0.5292 | 88.4749 | 0.0000 | 0.2181 | 78.5831 | 4.6692 |
| **0.3381** | 0.1062 | 3.1824 | 0.0015 | 0.1299 | 0.5464 | 92.1752 | 0.0000 | 0.1936 | 78.4429 | 4.6388 |
| **0.2912** | 0.1062 | 2.7415 | 0.0061 | 0.0830 | 0.4993 | 89.5676 | 0.0000 | 0.1886 | 77.7944 | 4.5034 |
| **0.3184** | 0.1132 | 2.8119 | 0.0049 | 0.0965 | 0.5404 | 94.1954 | 0.0000 | 0.2223 | 79.8381 | 4.9599 |
| **0.3332** | 0.1073 | 3.1052 | 0.0019 | 0.1229 | 0.5436 | 93.3619 | 0.0000 | 0.1999 | 78.9974 | 4.7613 |
| **0.3447** | 0.1063 | 3.2440 | 0.0012 | 0.1365 | 0.5530 | 91.0203 | 0.0000 | 0.1885 | 77.7478 | 4.4939 |
| **0.3300** | 0.1113 | 2.9647 | 0.0030 | 0.1118 | 0.5482 | 94.5621 | 0.0000 | 0.2147 | 79.8843 | 4.9712 |
| **0.3272** | 0.1119 | 2.9232 | 0.0035 | 0.1078 | 0.5467 | 94.7580 | 0.0000 | 0.2176 | 80.0294 | 5.0074 |
| **0.3254** | 0.1092 | 2.9783 | 0.0029 | 0.1112 | 0.5395 | 94.7003 | 0.0000 | 0.2088 | 79.6826 | 4.9219 |
| **0.3466** | 0.1071 | 3.2352 | 0.0012 | 0.1366 | 0.5566 | 88.4783 | 0.0000 | 0.1896 | 77.2682 | 4.3991 |
| **0.3287** | 0.1084 | 3.0329 | 0.0024 | 0.1163 | 0.5411 | 94.3092 | 0.0000 | 0.2052 | 79.4162 | 4.8582 |
| **0.3364** | 0.1120 | 3.0036 | 0.0027 | 0.1169 | 0.5559 | 92.5012 | 0.0000 | 0.2144 | 78.5134 | 4.6541 |
| **0.3380** | 0.1059 | 3.1918 | 0.0014 | 0.1305 | 0.5456 | 91.8069 | 0.0000 | 0.1925 | 78.3584 | 4.6207 |
| **0.3377** | 0.1119 | 3.0176 | 0.0025 | 0.1184 | 0.5571 | 90.1950 | 0.0000 | 0.2133 | 77.3573 | 4.4164 |
| **0.3038** | 0.1076 | 2.8230 | 0.0048 | 0.0929 | 0.5148 | 93.0935 | 0.0000 | 0.2004 | 79.0130 | 4.7649 |
| **0.3273** | 0.1116 | 2.9326 | 0.0034 | 0.1085 | 0.5460 | 94.7450 | 0.0000 | 0.2164 | 80.0163 | 5.0041 |
| **0.2709** | 0.0983 | 2.7559 | 0.0059 | 0.0783 | 0.4636 | 82.0988 | 0.0000 | 0.1494 | 73.4293 | 3.7635 |
| **0.3102** | 0.1121 | 2.7664 | 0.0057 | 0.0904 | 0.5300 | 92.8369 | 0.0000 | 0.2170 | 79.7158 | 4.9300 |

**Table 3.** Leave-one-out analyses for family history of TC.

| **estimate** | **se** | **zval** | **pval** | **ci.lb** | **ci.ub** | **Q** | **Qp** | **tau2** | **I2** | **H2** |
| --- | --- | --- | --- | --- | --- | --- | --- | --- | --- | --- |
| **0.4429** | 0.1428 | 3.1012 | 0.0019 | 0.1630 | 0.7228 | 3.4403 | 0.4870 | 0.0000 | 0.0000 | 1.0000 |
| **0.4712** | 0.1228 | 3.8381 | 0.0001 | 0.2306 | 0.7118 | 2.9147 | 0.5722 | 0.0000 | 0.0000 | 1.0000 |
| **0.4358** | 0.1229 | 3.5458 | 0.0004 | 0.1949 | 0.6767 | 1.7676 | 0.7784 | 0.0000 | 0.0000 | 1.0000 |
| **0.4405** | 0.1971 | 2.2349 | 0.0254 | 0.0542 | 0.8268 | 3.4715 | 0.4822 | 0.0000 | 0.0000 | 1.0000 |
| **0.4569** | 0.1226 | 3.7269 | 0.0002 | 0.2166 | 0.6972 | 3.4707 | 0.4823 | 0.0000 | 0.0010 | 1.0000 |
| **0.4911** | 0.1250 | 3.9278 | 0.0001 | 0.2460 | 0.7361 | 2.2289 | 0.6937 | 0.0000 | 0.0000 | 1.0000 |

**Figure 3.** Forest plots of individual and pooled estimates for the association between TSH levels and malignancy, stratified by study design (A) and by diagnostic method (B).

**Figure 4**. Forest plots of individual and pooled estimates for the association between nodule size and malignancy, stratified by number of nodules analysed per study.

**Figure 5.** Forest plots of individual and pooled estimates for the association between number of nodules and malignancy, stratified by study continent.

**Figure 6.** Forest plots of individual and pooled estimates for the association between solid composition and malignancy, stratified by diagnostic method.

**Figure 7.** Forest plots of individual and pooled estimates for the association between calcifications and malignancy, stratified by year of publication.

**Figure 8.** Forest plots of individual and pooled estimates for the association between microcalcifications and malignancy, stratified by diagnostic method.

**Table 4.** Leave-one-out analyses for nodule shape.

| **estimate** | **se** | **zval** | **pval** | **ci.lb** | **ci.ub** | **Q** | **Qp** | **tau2** | **I2** | **H2** |
| --- | --- | --- | --- | --- | --- | --- | --- | --- | --- | --- |
| **1.7706** | 0.2528 | 7.0048 | 0.0000 | 1.2752 | 2.2661 | 36.0252 | 0.0002 | 0.4756 | 75.0276 | 4.0044 |
| **1.7058** | 0.2914 | 5.8535 | 0.0000 | 1.1346 | 2.2769 | 146.6327 | 0.0000 | 0.7269 | 89.6525 | 9.6641 |
| **1.6322** | 0.2824 | 5.7803 | 0.0000 | 1.0788 | 2.1857 | 146.5520 | 0.0000 | 0.7300 | 90.1342 | 10.1360 |
| **1.6363** | 0.3071 | 5.3281 | 0.0000 | 1.0344 | 2.2382 | 116.6849 | 0.0000 | 0.8105 | 87.3077 | 7.8788 |
| **1.5874** | 0.2877 | 5.5184 | 0.0000 | 1.0236 | 2.1513 | 143.4557 | 0.0000 | 0.7361 | 90.1263 | 10.1279 |
| **1.7178** | 0.2917 | 5.8899 | 0.0000 | 1.1462 | 2.2894 | 146.6090 | 0.0000 | 0.7141 | 88.4739 | 8.6760 |
| **1.5377** | 0.2786 | 5.5184 | 0.0000 | 0.9915 | 2.0838 | 138.6161 | 0.0000 | 0.6737 | 89.2761 | 9.3250 |
| **1.4444** | 0.2517 | 5.7392 | 0.0000 | 0.9511 | 1.9376 | 118.0235 | 0.0000 | 0.4980 | 85.6877 | 6.9870 |
| **1.6862** | 0.2951 | 5.7142 | 0.0000 | 1.1078 | 2.2645 | 146.4404 | 0.0000 | 0.7557 | 90.1170 | 10.1184 |
| **1.6091** | 0.2975 | 5.4081 | 0.0000 | 1.0260 | 2.1923 | 142.3360 | 0.0000 | 0.7758 | 90.4080 | 10.4253 |
| **1.5771** | 0.2957 | 5.3328 | 0.0000 | 0.9975 | 2.1568 | 135.7423 | 0.0000 | 0.7544 | 89.9830 | 9.9830 |
| **1.5737** | 0.2907 | 5.4128 | 0.0000 | 1.0039 | 2.1435 | 140.1581 | 0.0000 | 0.7374 | 90.0217 | 10.0218 |
| **1.5995** | 0.3032 | 5.2762 | 0.0000 | 1.0053 | 2.1937 | 124.3423 | 0.0000 | 0.7891 | 89.4271 | 9.4581 |

**Table 5.** Leave-one-out analyses for margins.

| **estimate** | **se** | **zval** | **pval** | **ci.lb** | **ci.ub** | **Q** | **Qp** | **tau2** | **I2** | **H2** |
| --- | --- | --- | --- | --- | --- | --- | --- | --- | --- | --- |
| **1.9630** | 0.3489 | 5.6266 | 0.0000 | 1.2792 | 2.6468 | 57.9323 | 0.0000 | 1.2349 | 86.4939 | 7.4040 |
| **1.9385** | 0.3351 | 5.7851 | 0.0000 | 1.2818 | 2.5953 | 57.7590 | 0.0000 | 1.1587 | 91.5351 | 11.8135 |
| **2.0578** | 0.3202 | 6.4268 | 0.0000 | 1.4302 | 2.6853 | 55.3310 | 0.0000 | 1.0577 | 90.8971 | 10.9855 |
| **1.9199** | 0.3414 | 5.6230 | 0.0000 | 1.2507 | 2.5891 | 51.6977 | 0.0000 | 1.1730 | 88.5413 | 8.7270 |
| **2.0061** | 0.3378 | 5.9386 | 0.0000 | 1.3440 | 2.6682 | 57.4778 | 0.0000 | 1.1796 | 91.6596 | 11.9898 |
| **1.9611** | 0.3472 | 5.6477 | 0.0000 | 1.2805 | 2.6417 | 58.1408 | 0.0000 | 1.2268 | 90.6236 | 10.6650 |
| **1.9540** | 0.3423 | 5.7087 | 0.0000 | 1.2831 | 2.6248 | 58.0727 | 0.0000 | 1.2010 | 91.6125 | 11.9225 |
| **1.9167** | 0.3228 | 5.9372 | 0.0000 | 1.2839 | 2.5494 | 56.8145 | 0.0000 | 1.0816 | 91.0872 | 11.2198 |
| **1.8734** | 0.3141 | 5.9644 | 0.0000 | 1.2578 | 2.4890 | 53.2361 | 0.0000 | 0.9791 | 90.0543 | 10.0545 |
| **2.0059** | 0.3416 | 5.8726 | 0.0000 | 1.3364 | 2.6753 | 57.1412 | 0.0000 | 1.1973 | 91.6277 | 11.9441 |
| **2.0004** | 0.3438 | 5.8184 | 0.0000 | 1.3265 | 2.6742 | 57.1195 | 0.0000 | 1.2098 | 91.5658 | 11.8565 |
| **1.8990** | 0.3304 | 5.7481 | 0.0000 | 1.2515 | 2.5465 | 54.8753 | 0.0000 | 1.0965 | 90.7877 | 10.8551 |
| **2.2655** | 0.1383 | 16.3801 | 0.0000 | 1.9944 | 2.5366 | 22.2647 | 0.0347 | 0.0744 | 40.7755 | 1.6885 |
| **2.0213** | 0.3404 | 5.9373 | 0.0000 | 1.3541 | 2.6886 | 55.9763 | 0.0000 | 1.1837 | 91.4672 | 11.7194 |

**Bibliography**

1.	Ageeli RS, Mossery RA, Othathi RJ, Khawaji EA, Tarshi MM, Khormi GJ, Bingasem SM, Khmees RA, Aburasain NS, Al Ghadeeb M. The Importance of the Thyroid Nodule Location in Determining the Risk of Malignancy: A Retrospective Study. *Cureus* 14:e29421. doi: 10.7759/cureus.29421

2.	Ajarma KY, Al-Faouri AF, Al Ruhaibeh MK, Almbaidien FA, Nserat RT, Al-Shawabkeh AO, Al-Sarihin KK, Al-Harazi YA, Rbihat HS, Aljbour ME. The risk of thyroid carcinoma in multinodular goiter compared to solitary thyroid nodules: A retrospective analysis of 600 patients. *Med J Armed Forces India* (2020) 76:23–29. doi: 10.1016/j.mjafi.2018.05.001

3.	Al Dawish MA, Alwin Robert A, Thabet MA, Braham R. Thyroid Nodule Management: Thyroid-Stimulating Hormone, Ultrasound, and Cytological Classification System for Predicting Malignancy. *Cancer Inform* (2018) 17:1176935118765132. doi: 10.1177/1176935118765132

4.	Al Dawish M, Alwin Robert A, Al Shehri K, Hawsawi S, Mujammami M, Al Basha IA, Alrasheed M, Asiri S, Alzouman M, Alkharashi E. Risk Stratification of Thyroid Nodules with Bethesda III Category: The Experience of a Territorial Healthcare Hospital. *Cureus* (2020) 12:e8202. doi: 10.7759/cureus.8202

5.	Alaraifi AK, Alessa M, Hijazi LO, Alayed AM, Alsalem AA. TSH level as a risk factor of thyroid malignancy for nodules in euthyroid patients. *Acta Otorhinolaryngol Ital* (2023) 43:183–188. doi: 10.14639/0392-100X-N2288

6.	Al-Bader A., Zawawi F., Singer Z., Mlynarek A., Hier M., Tamilia M., Payne R. Preoperative TSH and thyroglobulin levels: Would it predict thyroid cancer? *Otolaryngol Pol* (2015) 69:21–25. doi: 10.5604/00306657.1156331

7.	Alqahtani SM, Altalhi BA, Alalawi YS, Al-Sobhi SS. Is the nodule location a predictive risk factor for cancer in AUS/FLUS thyroid nodules? A retrospective cohort study. *Asian J Surg* (2024) 47:2574–2578. doi: 10.1016/j.asjsur.2024.02.096

8.	Angell TE, Vyas CM, Medici M, Wang Z, Barletta JA, Benson CB, Cibas ES, Cho NL, Doherty GM, Doubilet PM, et al. Differential Growth Rates of Benign vs. Malignant Thyroid Nodules. *J Clin Endocrinol Metab* (2017) 102:4642–4647. doi: 10.1210/jc.2017-01832

9.	Angell TE, Maurer R, Wang Z, Kim MI, Alexander CA, Barletta JA, Benson CB, Cibas ES, Cho NL, Doherty GM, et al. A Cohort Analysis of Clinical and Ultrasound Variables Predicting Cancer Risk in 20,001 Consecutive Thyroid Nodules. *J Clin Endocrinol Metab* (2019) 104:5665–5672. doi: 10.1210/jc.2019-00664

10.	Apostolou K, Zivaljevic V, Tausanovic K, Zoric G, Chelidonis G, Slijepcevic N, Jovanovic M, Paunovic I. Prevalence and risk factors for thyroid cancer in patients with multinodular goitre. *BJS Open* (2021) 5:zraa014. doi: 10.1093/bjsopen/zraa014

11.	Arduc A, Dogan BA, Tuna MM, Tutuncu Y, Isik S, Berker D, Guler S. Higher body mass index and larger waist circumference may be predictors of thyroid carcinoma in patients with Hürthle-cell lesion/neoplasm fine-needle aspiration diagnosis. *Clin Endocrinol (Oxf)* (2015) 83:405–411. doi: 10.1111/cen.12628

12.	Aydin Y., Turgut M., Erkan M.E., Ozaydin I., Oktay M., Kutlucan A., Onder E., Gur M., Sav T. Comparison of folicular, Hurthle cell neoplasm and oncocytic changes diagnosed by thyroid fine needle aspiration with postoperative histopathological results. *Turk J Endocrinol Metab* (2014) 18:5–12. doi: 10.4274/tjem.2408

13.	Babayid Y, Gökçay Canpolat A, Elhan AH, Ceyhan K, Çorapçıoğlu D, Şahin M. Should there be a paradigm shift for the evaluation of isthmic thyroid nodules? *J Endocrinol Invest* (2024) 47:2225–2233. doi: 10.1007/s40618-024-02313-6

14.	Baser H, Topaloglu O, Tam AA, Evranos B, Alkan A, Sungu N, Dumlu EG, Ersoy R, Cakir B. Higher TSH can be used as an additional risk factor in prediction of malignancy in euthyroid thyroid nodules evaluated by cytology based on Bethesda system. *Endocrine* (2016) 53:520–529. doi: 10.1007/s12020-016-0919-4

15.	Batawil N, Alkordy T. Ultrasonographic features associated with malignancy in cytologically indeterminate thyroid nodules. *Eur J Surg Oncol* (2014) 40:182–186. doi: 10.1016/j.ejso.2013.11.015

16.	Behbahaninia M, Nabahati M, Moazezi Z, Mehraeen R, Shirafkan H. Diagnostic performance of ACR-TIRADS, Korean TIRADS, and American Thyroid Association guidelines for risk stratification of thyroid nodules: a prospective study. *J Ultrasound* (2022) 25:887–894. doi: 10.1007/s40477-022-00669-0

17.	Boonrod A, Akkus Z, Castro MR, Zeinodini A, Philbrick K, Stan M, Erickson D, Erickson B. Thyroid Nodule Size as a Predictor of Malignancy in Follicular and Hurthle Neoplasms. *Asian Pac J Cancer Prev* (2021) 22:2597–2602. doi: 10.31557/APJCP.2021.22.8.2597

18.	Brophy C, Stewart J, O’Donovan N, McCarthy J, Murphy M, Sheahan P. Impact of Microcalcifications on Risk of Malignancy in Thyroid Nodules with Indeterminate or Benign Cytology. *Otolaryngol Head Neck Surg* (2016) 154:46–51. doi: 10.1177/0194599815605326

19.	Bukasa Kakamba J, Sabbah N, Bayauli P, Massicard M, Bidingija J, Nkodila A, Mbunga B, Ditu S, Beckers A, Potorac I. Thyroid cancer in the Democratic Republic of the Congo: Frequency and risk factors. *Ann Endocrinol (Paris)* (2021) 82:606–612. doi: 10.1016/j.ando.2021.09.002

20.	Campbell MJ, Candell L, Seib CD, Gosnell JE, Duh Q-Y, Clark OH, Shen WT. Unanticipated thyroid cancer in patients with substernal goiters: are we underestimating the risk? *Ann Surg Oncol* (2015) 22:1214–1218. doi: 10.1245/s10434-014-4143-2

21.	Canete EJ, Sison-Pena CM, Jimeno CA. Clinicopathological, Biochemical, and Sonographic Features of Thyroid Nodule Predictive of Malignancy among Adult Filipino Patients in a Tertiary Hospital in the Philippines. *Endocrinol Metab (Seoul)* (2014) 29:489–497. doi: 10.3803/EnM.2014.29.4.489

22.	Cao S-L, Shi W-Y, Niu Y-R, Zhao Z-L, Wei Y, Wu J, Peng L-L, Li Y, Yu M-A. Influence of maximum diameter on fine-needle aspiration biopsy outcomes in ACR TI-RADS 5 thyroid nodules. *Front Endocrinol* (2024) 15:1374888. doi: 10.3389/fendo.2024.1374888

23.	Cappelli C, Pirola I, Gandossi E, Marini F, Cristiano A, Casella C, Lombardi D, Agosti B, Ferlin A, Castellano M. Ultrasound Microvascular Blood Flow Evaluation: A New Tool for the Management of Thyroid Nodule? *Int J Endocrinol* (2019) 2019:7874890. doi: 10.1155/2019/7874890

24.	Castagna MG, Belardini V, Memmo S, Maino F, Di Santo A, Toti P, Carli AF, Caruso G, Pacini F. Nodules in autoimmune thyroiditis are associated with increased risk of thyroid cancer in surgical series but not in cytological series: evidence for selection bias. *J Clin Endocrinol Metab* (2014) 99:3193–3198. doi: 10.1210/jc.2014-1302

25.	Cavallo A, Johnson DN, White MG, Siddiqui S, Antic T, Mathew M, Grogan RH, Angelos P, Kaplan EL, Cipriani NA. Thyroid Nodule Size at Ultrasound as a Predictor of Malignancy and Final Pathologic Size. *Thyroid* (2017) 27:641–650. doi: 10.1089/thy.2016.0336

26.	Chen W-H, Chen C-Y. Clinicopathologic characteristics of incidental thyroid carcinoma in euthyroid patients receiving total thyroidectomy for multinodular goiter: A retrospective cohort study. *J Chin Med Assoc* (2022) 85:839–844. doi: 10.1097/JCMA.0000000000000758

27.	Chen Z, Lin Y, Lai S, Wang P, Li J, Wang L, Guan H, Kuang J. The utility of serum anti-thyroglobulin antibody and thyroglobulin in the preoperative differential diagnosis of thyroid follicular neoplasms. *Endocrine* (2022) 76:369–376. doi: 10.1007/s12020-022-02993-1

28.	Chen X, Hu Z, Sun D, Luo H, Zhao C, Liao M. Diagnostic value of American College of Radiology Thyroid Imaging Reporting and Data System combined with elastography in differentiating clinically atypical subacute thyroiditis from papillary thyroid carcinoma: a single retrospective research. *Egyptian Journal of Radiology and Nuclear Medicine* (2023) 54:214. doi: 10.1186/s43055-023-01159-x

29.	Chiefari E, Innaro N, Gervasi R, Mirabelli M, Giuliano S, Donnici A, Obiso S, Brunetti FS, Foti DP, Brunetti A. Incidental thyroid carcinoma in an endemic goiter area in Italy: histopathological features and predictors of a common finding. *Endocrine* (2024) 84:589–597. doi: 10.1007/s12020-023-03659-2

30.	Chiofalo MG, Signoriello S, Fulciniti F, Avenia N, Ristagno S, Lombardi CP, Nicolosi A, Pelizzo MR, Perigli G, Polistena A, et al. Predictivity of clinical, laboratory and imaging findings in diagnostic definition of palpable thyroid nodules. A multicenter prospective study. *Endocrine* (2018) 61:43–50. doi: 10.1007/s12020-018-1577-5

31.	Cho YY, Chung YJ, Kim HS. Malignancy Rate of Bethesda Class III Thyroid Nodules Based on the Presence of Chronic Lymphocytic Thyroiditis in Surgical Patients. *Front Endocrinol (Lausanne)* (2021) 12:745395. doi: 10.3389/fendo.2021.745395

32.	Choi JS, Nam CM, Kim EK, Moon HJ, Han KH, Kwak JY. Evaluation of serum thyroid-stimulating hormone as indicator for fine-needle aspiration in patients with thyroid nodules. *Head Neck* (2015) 37:498–504. doi: 10.1002/hed.23616

33.	Cozzolino A, Pozza C, Pofi R, Sbardella E, Faggiano A, Isidori AM, Giannetta E, Pernazza A, Rullo E, Ascoli V, et al. Predictors of malignancy in high-risk indeterminate (TIR3B) cytopathology thyroid nodules. *J Endocrinol Invest* (2020) 43:1115–1123. doi: 10.1007/s40618-020-01200-0

34.	Crudele L, Novielli F, De Matteis C, Petruzzelli S, Suppressa P, Berardi E, Antonica G, Piazzolla G, Sabbà C, Graziano G, et al. Thyroid nodule malignancy is associated with increased non-invasive hepatic fibrosis scores in metabolic subjects. *Front Oncol* (2023) 13:1233083. doi: 10.3389/fonc.2023.1233083

35.	Delman AM, Turner KM, Ammann AM, Sisak S, Farooqui Z, Holm TM. The national rate of malignancy among Bethesda III, IV, and V thyroid nodules is higher than expected: A NSQIP analysis. *Surgery* (2023) 173:645–652. doi: 10.1016/j.surg.2022.06.049

36.	Deng X-H, Tang L-N, Liu S-Q, Li X-L, He Y-P, Xu H-X. A Proposal to Stratify the Intermediate-Risk Thyroid Nodules According to the AACE/ACE/AME Guidelines with Ultrasound Features. *Sci Rep* (2017) 7:17901. doi: 10.1038/s41598-017-18207-y

37.	Deng Y, Zhang J, Zou G, Li S, Gong Z, Yue G, Fan P, Xu J. Peripheral Blood Inflammatory Markers Can Predict Benign and Malignant Thyroid Nodules. *Int J Endocrinol* (2022) 2022:2319660. doi: 10.1155/2022/2319660

38.	Ding J, Jiang L, Fang J, Jiang Y, Zhu Y, Hua T, Yuan Y, Wu W. Predictors for malignancy risk in subcentimeter thyroid nodules categorized as atypia/follicular lesion of undetermined significance by fine needle aspiration. *Sci Rep* (2019) 9:14973. doi: 10.1038/s41598-019-50597-z

39.	Dobruch-Sobczak K, Adamczewski Z, Szczepanek-Parulska E, Migda B, Woliński K, Krauze A, Prostko P, Ruchała M, Lewiński A, Jakubowski W, et al. Histopathological Verification of the Diagnostic Performance of the EU-TIRADS Classification of Thyroid Nodules-Results of a Multicenter Study Performed in a Previously Iodine-Deficient Region. *J Clin Med* (2019) 8:1781. doi: 10.3390/jcm8111781

40.	Dong S, Pan J, Shen Y-B, Zhu L-X, Xia Q, Xie X-J, Wu Y-J. Factors Associated with Malignancy in Patients with Maximal Thyroid Nodules ≥2 Cm. *Cancer Manag Res* (2021) 13:4473–4482. doi: 10.2147/CMAR.S303715

41.	Du J, Li W, Zhao X, Shen C, Zhang X. Establishment of a predictive nomogram for differentiated thyroid cancer: an inpatient-based retrospective study. *Endokrynol Pol* (2023) 74: doi: 10.5603/ep.97087

42.	Ebrahim H, Tilahun ,Mihret, Fiseha ,Temesgen, Debash ,Habtu, Bisetegn ,Habtye, Alemayehu ,Ermiyas, Fiseha ,Mesfin, Ebrahim ,Endris, Shibabaw ,Agumas, Seid ,Abdurahaman, et al. Patterns of Fine Needle Aspiration Cytology Diagnosed Thyroid Nodules Among Clinically Suspected Patients in Northeast Ethiopia. *Pathology and Laboratory Medicine International* (2023) 15:27–36. doi: 10.2147/PLMI.S399682

43.	Egset AV, Holm C, Larsen SR, Nielsen SH, Bach J, Helweg-Larsen JP, Larsen LV, Wanscher JH, Godballe C. Risk of malignancy in fine-needle aspiration biopsy in patients with thyroid nodules. *Dan Med J* (2017) 64:A5320.

44.	Eisa N, Khan A, Akhter M, Fensterwald M, Saleem S, Fananapazir G, Campbell MJ. Both Ultrasound Features and Nuclear Atypia are Associated with Malignancy in Thyroid Nodules with Atypia of Undetermined Significance. *Ann Surg Oncol* (2018) 25:3913–3918. doi: 10.1245/s10434-018-6826-6

45.	Eissa MS, Abdellateif MS, Elesawy YF, Shaarawy S, Al-Jarhi UM. Obesity and Waist Circumference are Possible Risk Factors for Thyroid Cancer: Correlation with Different Ultrasonography Criteria. *Cancer Manag Res* (2020) 12:6077–6089. doi: 10.2147/CMAR.S256268

46.	El Sayed Ibrahim MM, Omar W, Elhofy A. Prospective study evaluating malignancy in solitary thyroid nodule. *The Egyptian Journal of Surgery* (2019) 38:411. doi: 10.4103/ejs.ejs_8_19

47.	Elbalka SS, Metwally IH, Shetiwy M, Awny S, Hamdy O, Kotb SZ, Shoman AM, Shahda E, Elzahaby IA. Prevalence and predictors of thyroid cancer among thyroid nodules: a retrospective cohort study of 1,000 patients. *Ann R Coll Surg Engl* (2021) 103:683–689. doi: 10.1308/rcsann.2021.0057

48.	Espinosa De Ycaza AE, Lowe KM, Dean DS, Castro MR, Fatourechi V, Ryder M, Morris JC, Stan MN. Risk of Malignancy in Thyroid Nodules with Non-Diagnostic Fine-Needle Aspiration: A Retrospective Cohort Study. *Thyroid* (2016) 26:1598–1604. doi: 10.1089/thy.2016.0096

49.	Fernández-Trujillo C, Pérez-Zaballos J, Rodríguez-Pérez CA, López-Plasencia Y, Marrero-Arencibia D, Cabrera-Galván JJ, Boronat M. TSH Level and Risk of Malignancy in Patients with Bethesda Category IV Thyroid Nodules. *Horm Cancer* (2020) 11:200–204. doi: 10.1007/s12672-020-00384-4

50.	Fighera TM, Perez CLS, Faris N, Scarabotto PC, da Silva TT, Cavalcanti TCS, Mesa Junior CO, Miasaki F, da Paz Filho GJ, de Carvalho GA. TSH levels are associated with increased risk of thyroid carcinoma in patients with nodular disease. *Endokrynol Pol* (2015) 66:480–485. doi: 10.5603/EP.a2015.0059

51.	GallegosVargas J, SanchezRoldan J, RonquilloSanchez M, Carmona Aparicio L, FlorianoSanchez E, CardenasRodriguez N. Gene Expression of CYP1A1 and its Possible Clinical Application in Thyroid Cancer Cases. *Asian Pac J Cancer Prev* (2016) 17:3477–3482.

52.	Garino F, Deandrea M, Motta M, Mormile A, Ragazzoni F, Palestini N, Freddi M, Gasparri G, Sgotto E, Pacchioni D, et al. Diagnostic performance of elastography in cytologically indeterminate thyroid nodules. *Endocrine* (2015) 49:175–183. doi: 10.1007/s12020-014-0438-0

53.	Gezer E, Demirkan H, Cantürk Z, Selek A, Çetinarslan B, Sözen M, Çalis Kan M, Çağrı Karci A, Bilgen Özcan T, Topaloğlu Ö, et al. Association of ultrasonographically determined anatomical location of Bethesda 3 thyroid nodules with differentiated thyroid cancer. *Qatar Med J* (2022) 2022:51. doi: 10.5339/qmj.2022.51

54.	Grani G, Calvanese A, Carbotta G, D’Alessandri M, Nesca A, Bianchini M, Del Sordo M, Vitale M, Fumarola A. Thyroid autoimmunity and risk of malignancy in thyroid nodules submitted to fine-needle aspiration cytology. *Head Neck* (2015) 37:260–264. doi: 10.1002/hed.23587

55.	Grani G, Del Gatto V, Cantisani V, Mandel SJ, Durante C. A Reappraisal of Suspicious Sonographic Features of Thyroid Nodules: Shape Is Not an Independent Predictor of Malignancy. *J Clin Endocrinol Metab* (2023) 108:e816–e822. doi: 10.1210/clinem/dgad092

56.	Gu W-J, Yan H-X, Luo Y-K, Wang F-L, Yang G-Q, Guo Q-H, Jin N, Zang L, Chen K, Du J, et al. Characterization of papillary thyroid microcarcinomas using sonographic features in malignant papillary thyroid cancer: a retrospective analysis. *Medicine (Baltimore)* (2015) 94:e841. doi: 10.1097/MD.0000000000000841

57.	Guarnotta V, La Monica R, Ingrao VR, Di Stefano C, Salzillo R, Pizzolanti G, Giannone AG, Almasio PL, Richiusa P, Giordano C. Ultrasound Parameters Can Accurately Predict the Risk of Malignancy in Patients with “Indeterminate TIR3b” Cytology Nodules: A Prospective Study. *Int J Mol Sci* (2023) 24:8296. doi: 10.3390/ijms24098296

58.	Gweon HM, Son EJ, Kim J-A, Youk JH. Predictive Factors for Active Surveillance of Subcentimeter Thyroid Nodules with Highly Suspicious US Features. *Ann Surg Oncol* (2017) 24:1540–1545. doi: 10.1245/s10434-016-5728-8

59.	Hacim NA, Akbaş A, Ulgen Y, Aktokmakyan TV, Meric S, Tokocin M, Karabay O, Altinel Y. Impact of Ultrasonographic Features for Thyroid Malignancy in Patients With Bethesda Categories III, IV, and V: A Retrospective Observational Study in a Tertiary Center. *Cureus* (2021) 13:e16708. doi: 10.7759/cureus.16708

60.	Handelsman RS, Alvarez AL, Picado O, Farrá JC, Lew JI. Inverse Relationship of BMI to TSH and Risk of Papillary Thyroid Cancer in Surgical Patients. *J Surg Res* (2019) 244:96–101. doi: 10.1016/j.jss.2019.06.017

61.	Hong IK, Kim JH, Cho YU, Park S-Y, Kim SJ. Clinicopathological factors increased the risk of malignancy in thyroid nodules with atypical or follicular lesions of undetermined significance (AUS/FLUS) risk factor of malignancy in thyroid nodule with AUS/FLUS. *Ann Surg Treat Res* (2016) 90:201–206. doi: 10.4174/astr.2016.90.4.201

62.	Hong S-H, Lee H, Cho M-S, Lee JE, Sung Y-A, Hong YS. Malignancy Risk and Related Factors of Atypia of Undetermined Significance/Follicular Lesion of Undetermined Significance in Thyroid Fine Needle Aspiration. *Int J Endocrinol* (2018) 2018:4521984. doi: 10.1155/2018/4521984

63.	Hou C, Wei R, Tang J, Hu Q, He H, Fan X. Diagnostic value of ultrasound features and sex of fetuses in female patients with papillary thyroid microcarcinoma. *Sci Rep* (2018) 8:7510. doi: 10.1038/s41598-018-26003-5

64.	Huan Q, Wang K, Lou F, Zhang L, Huang Q, Han Y, Sun H, Zhu L, Lin P, Song J, et al. Epidemiological characteristics of thyroid nodules and risk factors for malignant nodules: a retrospective study from 6,304 surgical cases. *Chin Med J (Engl)* (2014) 127:2286–2292.

65.	Huang J, Lin C, Chen Y, Li X. Clinical preliminary study on the correlation between nodular goitre and papillary thyroid carcinoma. *Transl Cancer Res* (2020) 9:3794–3803. doi: 10.21037/tcr-19-2951

66.	Huang J, Shi H, Song M, Liang J, Zhang Z, Chen X, Liu Y, Wang S, Wu Z. Surgical Outcome and Malignant Risk Factors in Patients With Thyroid Nodule Classified as Bethesda Category III. *Front Endocrinol (Lausanne)* (2021) 12:686849. doi: 10.3389/fendo.2021.686849

67.	Hwang SH, Kim E-K, Moon HJ, Yoon JH, Kwak JY. Risk of Thyroid Cancer in Euthyroid Asymptomatic Patients with Thyroid Nodules with an Emphasis on Family History of Thyroid Cancer. *Korean J Radiol* (2016) 17:255–263. doi: 10.3348/kjr.2016.17.2.255

68.	Ito Y, Hirokawa M, Miyauchi A, Kihara M, Yabuta T, Masuoka H, Fukushima M, Higashiyama T, Kobayashi K, Miya A. Diagnosis and surgical indications of oxyphilic follicular tumors in Japan: Surgical specimens and cytology. *Endocr J* (2016) 63:977–982. doi: 10.1507/endocrj.EJ16-0268

69.	Jabar ASS, Koteshwara P, Andrade J. Diagnostic reliability of the Thyroid Imaging Reporting and Data System (TI-RADS) in routine practice. *Pol J Radiol* (2019) 84:e274–e280. doi: 10.5114/pjr.2019.86823

70.	Jasim S, Baranski TJ, Teefey SA, Middleton WD. Investigating the Effect of Thyroid Nodule Location on the Risk of Thyroid Cancer. *Thyroid* (2020) 30:401–407. doi: 10.1089/thy.2019.0478

71.	Jia X, Pang P, Wang L, Zhao L, Jiang L, Song Y, Fan X, Wang Y, Zhao S, Ba J, et al. Clinical Analysis of Preoperative Anti-thyroglobulin Antibody in Papillary Thyroid Cancer Between 2011 and 2015 in Beijing, China: A Retrospective Study. *Front Endocrinol (Lausanne)* (2020) 11:452. doi: 10.3389/fendo.2020.00452

72.	Kaliszewski K, Diakowska D, Wojtczak B, Forkasiewicz Z. Evaluation of selected ultrasound features of thyroid nodules with atypia of undetermined significance/follicular lesion of undetermined significance for the Bethesda reporting system for thyroid cytology. *Cancer Manag Res* (2018) 10:2223–2229. doi: 10.2147/CMAR.S168409

73.	Kaliszewski K, Diakowska D, Rzeszutko M, Nowak Ł, Tokarczyk U, Rudnicki J. Atypia and Follicular Lesions of Undetermined Significance in Subsequent Biopsy Result: What Clinicians Need to Know. *J Clin Med* (2021) 10:3082. doi: 10.3390/jcm10143082

74.	Kaliszewski K, Diakowska D, Rzeszutko M, Wojtczak B, Rudnicki J. The Correlation of Age with Prognosis of Atypia of Undetermined Significance and Follicular Lesion of Undetermined Significance in Thyroid Nodules. *Cancer Manag Res* (2021) 13:3101–3111. doi: 10.2147/CMAR.S304686

75.	Kaliszewski K, Diakowska D, Rzeszutko M, Nowak Ł, Wojtczak B, Sutkowski K, Ludwig M, Ludwig B, Mikuła A, Greniuk M, et al. Assessment of Preoperative TSH Serum Level and Thyroid Cancer Occurrence in Patients with AUS/FLUS Thyroid Nodule Diagnosis. *Biomedicines* (2022) 10:1916. doi: 10.3390/biomedicines10081916

76.	Karabacak U, Derebey M, Tarim IA, Polat AK. FACTORS AFFECTING MALIGNANCY IN THYROID NODULES WITH NON-DIAGNOSTIC FINE NEEDLE ASPIRATION BIOPSY RESULT. *Acta Endocrinol (Buchar)* (2022) 18:187–193. doi: 10.4183/aeb.2022.187

77.	Khodabandelu S, Ghaemian N, Khafri S, Ezoji M, Khaleghi S. Development of a Machine Learning-Based Screening Method for Thyroid Nodules Classification by Solving the Imbalance Challenge in Thyroid Nodules Data. *J Res Health Sci* (2022) 22:e00555. doi: 10.34172/jrhs.2022.90

78.	Kim GR, Kim E-K, Kwak JY, Yoon JH, Moon HJ. Association between Bethesda Categories and Ultrasound Features of Conventional Papillary Thyroid Carcinoma. *Ultrasound Med Biol* (2016) 42:1066–1074. doi: 10.1016/j.ultrasmedbio.2015.12.019

79.	Kim HJ, Mok J-O, Kim CH, Kim YJ, Kim SJ, Park HK, Byun DW, Suh K, Yoo MH. Preoperative serum thyroglobulin and changes in serum thyroglobulin during TSH suppression independently predict follicular thyroid carcinoma in thyroid nodules with a cytological diagnosis of follicular lesion. *Endocr Res* (2017) 42:154–162. doi: 10.1080/07435800.2016.1262395

80.	Kim HJ, Kim NK, Park HK, Byun DW, Suh K, Yoo MH, Min Y-K, Kim SW, Chung JH. Strong association of relatively low and extremely excessive iodine intakes with thyroid cancer in an iodine-replete area. *Eur J Nutr* (2017) 56:965–971. doi: 10.1007/s00394-015-1144-2

81.	Kim K, Jung CK, Lim D-J, Bae JS, Kim JS. Clinical and pathologic features for predicting malignancy in thyroid follicular neoplasms. *Gland Surg* (2021) 10:50–58. doi: 10.21037/gs-20-500

82.	Kisioglu SV, Atacan H, Cakir E, Oguz S, Karakullukcu S, Ersoz HO. Efficacy of Multidisciplinary Approach in Thyroid Nodules: Analysis of Factors Used for Predicting Malignancy. *Turk J Endocrinol Metab* (2022) 26:125–130. doi: 10.5152/tjem.2022.22020

83.	Kong J, Li J-C, Wang H-Y, Wang Y-H, Zhao R-N, Zhang Y, Jin J. Role of Superb Micro-Vascular Imaging in the Preoperative Evaluation of Thyroid Nodules: Comparison With Power Doppler Flow Imaging. *J Ultrasound Med* (2017) 36:1329–1337. doi: 10.7863/ultra.16.07004

84.	Kornelius E, Lo S-C, Huang C-N, Yang Y-S. The Risk of Thyroid Cancer in Patients with Thyroid Nodule 3 Cm Or Larger. *Endocr Pract* (2020) 26:1286–1290. doi: 10.4158/EP-2020-0136

85.	Kotecka-Blicharz A, Krzempek M, Cortez AJ, Oczko-Wojciechowska M, Czarniecka A, Nożyńska E, Chmielik E, Jarząb B, Krajewska J. The role of thyroid sonographic malignancy risk features when the fine needle aspiration biopsy result is indeterminate. *Endokrynol Pol* (2022) 73:316–324. doi: 10.5603/EP.a2022.0027

86.	Krátký J, Ježková J, Kosák M, Vítková H, Bartáková J, Mráz M, Lukáš J, Límanová Z, Jiskra J. Positive Antithyroid Antibodies and Nonsuppressed TSH Are Associated with Thyroid Cancer: A Retrospective Cross-Sectional Study. *International Journal of Endocrinology* (2018) 2018:9793850. doi: 10.1155/2018/9793850

87.	Kuo T-C, Wu M-H, Chen K-Y, Hsieh M-S, Chen A, Chen C-N. Ultrasonographic features for differentiating follicular thyroid carcinoma and follicular adenoma. *Asian J Surg* (2020) 43:339–346. doi: 10.1016/j.asjsur.2019.04.016

88.	Kuru B, Atmaca A, Tarim IA, Kefeli M, Topgul K, Yoruker S, Elmali M, Danaci M. Risk factors associated with malignancy and with triage to surgery in thyroid nodules classified as Bethesda category III (AUS/FLUS). *Eur J Surg Oncol* (2016) 42:87–93. doi: 10.1016/j.ejso.2015.09.026

89.	Kuru B, Kefeli M. Risk factors associated with malignancy and with triage to surgery in thyroid nodules classified as Bethesda category IV (FN/SFN). *Diagn Cytopathol* (2018) 46:489–494. doi: 10.1002/dc.23923

90.	Lee KH, Shin JH, Oh YL, Hahn SY. Atypia of undetermined significance in thyroid fine-needle aspiration cytology: prediction of malignancy by US and comparison of methods for further management. *Ann Surg Oncol* (2014) 21:2326–2331. doi: 10.1245/s10434-014-3568-y

91.	Li T, Sheng J, Li W, Zhang X, Yu H, Chen X, Zhang J, Cai Q, Shi Y, Liu Z. A new computational model for human thyroid cancer enhances the preoperative diagnostic efficacy. *Oncotarget* (2015) 6:28463–28477. doi: 10.18632/oncotarget.4691

92.	Li W, Song Q, Lan Y, Li J, Zhang Y, Yan L, Li Y, Zhang Y, Luo Y. The Value of Sonography in Distinguishing Follicular Thyroid Carcinoma from Adenoma. *Cancer Manag Res* (2021) 13:3991–4002. doi: 10.2147/CMAR.S307166

93.	Li W, Gao L, Du Y, Wang Y, Yang X, Wang H, Li J. Ultrasound microflow patterns help in distinguishing malignant from benign thyroid nodules. *Cancer Imaging* (2024) 24:18. doi: 10.1186/s40644-024-00663-1

94.	Liu Y, Wu H, Zhou Q, Gou J, Xu J, Liu Y, Chen Q. Diagnostic Value of Conventional Ultrasonography Combined with Contrast-Enhanced Ultrasonography in Thyroid Imaging Reporting and Data System (TI-RADS) 3 and 4 Thyroid Micronodules. *Med Sci Monit* (2016) 22:3086–3094. doi: 10.12659/msm.897011

95.	Liu J, Zheng D, Li Q, Tang X, Luo Z, Yuan Z, Gao L, Zhao J. A predictive model of thyroid malignancy using clinical, biochemical and sonographic parameters for patients in a multi-center setting. *BMC Endocr Disord* (2018) 18:17. doi: 10.1186/s12902-018-0241-7

96.	Lourdes Ng J, Escueta LM, Dampil OA. Malignancy in Thyroid Nodules with Bethesda III Category on Repeat Fine Needle Aspiration Biopsy. *J ASEAN Fed Endocr Soc* (2023) 38:86–93. doi: 10.15605/jafes.038.02.19

97.	Lynch CA, Bethi M, Tang A, Lee P, Steward D, Holm TM. Thyroid nodules >4 cm with atypia of undetermined significance cytology independently associate with malignant pathology. *Surgery* (2022) 171:725–730. doi: 10.1016/j.surg.2021.08.017

98.	Macias CA, Arumugam D, Arlow RL, Eng OS, Lu S-E, Javidian P, Davidov T, Trooskin SZ. A risk model to determine surgical treatment in patients with thyroid nodules with indeterminate cytology. *Ann Surg Oncol* (2015) 22:1527–1532. doi: 10.1245/s10434-014-4190-8

99.	Maeda H, Kutomi G, Satomi F, Shima H, Mori M, Hirata K, Takemasa I. Clinicopathological characteristics of thyroid cancer misdiagnosed by fine needle aspiration. *Exp Ther Med* (2016) 12:2766–2772. doi: 10.3892/etm.2016.3651

100.	Mazzone M. Relation between Environmental, Lifestyle, Medical and Hormonal Risk Factors and Thyroid Tumour: A Cross-Sectional Study. *Clinics in Oncology* (2021) 6:

101.	Melak T, Mathewos B, Enawgaw B, Damtie D. Prevalence and types of thyroid malignancies among thyroid enlarged patients in Gondar, Northwest Ethiopia: a three years institution based retrospective study. *BMC Cancer* (2014) 14:899. doi: 10.1186/1471-2407-14-899

102.	Mileva M, Stoilovska B, Jovanovska A, Ugrinska A, Petrushevska G, Kostadinova-Kunovska S, Miladinova D, Majstorov V. Thyroid Cancer Detection Rate and Associated Risk Factors in Patients with Thyroid Nodules Classified As Bethesda Category III. *Radiol Oncol* (2018) 52:370–376. doi: 10.2478/raon-2018-0039

103.	Mohamed TZ, Sultan AAEA, Tag El-Din M, Mostafa AAE, Nafea MA, Kalmoush A-E, Nassar MS, Abdalgaleel MA, Hegab AM, Ibrahim AH, et al. Incidence and Risk Factors of Thyroid Malignancy in Patients with Toxic Nodular Goiter. *Int J Surg Oncol* (2022) 2022:1054297. doi: 10.1155/2022/1054297

104.	Molina-Vega M, Rodríguez-Pérez CA, Álvarez-Mancha AI, Baena-Nieto G, Riestra M, Alcázar V, Romero-Lluch AR, Galofré JC, Fernández-García JC. Clinical and Ultrasound Thyroid Nodule Characteristics and Their Association with Cytological and Histopathological Outcomes: A Retrospective Multicenter Study in High-Resolution Thyroid Nodule Clinics. *J Clin Med* (2019) 8:2172. doi: 10.3390/jcm8122172

105.	Moosa NA, Nasser MA, Alshehabi M. Thyroid cancer risk assessment indicators: A correlation between preoperative and postoperative criteria. *Neuro Endocrinol Lett* (2021) 42:417–422.

106.	Mu N, Juhlin CC, Tani E, Sofiadis A, Reihnér E, Zedenius J, Larsson C, Nilsson I-L. High Ki-67 index in fine needle aspiration cytology of follicular thyroid tumors is associated with increased risk of carcinoma. *Endocrine* (2018) 61:293–302. doi: 10.1007/s12020-018-1627-z

107.	Muhanhali D, Deng L, Ai Z, Ling Y. Impaired thyroid hormone sensitivity increases the risk of papillary thyroid cancer and cervical lymph node metastasis. *Endocrine* (2024) 83:659–670. doi: 10.1007/s12020-023-03508-2

108.	Naidu K, Saksenberg ,Victoria, and Goolam Mahyoodeen N. Clinical and ultrasound characteristics distinguishing benign and malignant thyroid nodules in Johannesburg, South Africa. *Journal of Endocrinology, Metabolism and Diabetes of South Africa* (2023) 28:62–68. doi: 10.1080/16089677.2023.2178274

109.	Nawarathna N, Ratnayake P, Hewage SN, Senevirathne RJ, Gunatilake SS, Kariyawasam NL, Shyamalie R, Yogachandran P. Association between Nonspecific Chronic Lymphocytic Thyroiditis and Differentiated Epithelial Thyroid Carcinoma: Clinicopathological Analysis of Patients Who underwent Thyroidectomy in a Tertiary Care Center in Sri Lanka. *World Journal of Endocrine Surgery* (2018) 10:119–126. doi: 10.5005/JP-JOURNALS-10002-1230

110.	Nguyen BQ, Tran HT, Nguyen HTT, Nguyen TX, Nguyen TTH, Nguyen TN, Vu HTT. Clinical, cytological and ultrasonographic features of incidental thyroid cancer in a hospital-based study in vietnam. *Endocrinol Diabetes Metab* (2023) 6:e415. doi: 10.1002/edm2.415

111.	Nitipir C, Alecu L, Slavu I, Tulin R, Jecan RC, Orlov C, Pituru S, Stanculeanu DL, Hainarosie R, Stoian AP, et al. Unmodifiable Variables Related to Thyroid Cancer Incidence. *Journal of Mind and Medical Sciences* (2018) 5:123–128. doi: 10.22543/7674.51.P123128

112.	Nonchev B, Danev V, Argatska A, Miteva M, Mateva N, Orbetzova M. Prognostic Model for Ultrasound Assessment of Malignancy Risk of Thyroid Nodules. *Endocrinologia* (2017) XXII:

113.	Öcal B, Korkmaz MH, Yılmazer D, Taşkın Türkmenoğlu T, Bayır Ö, Saylam G, Çadallı Tatar E, Karahan S, Çakal E. The Malignancy Risk Assessment of Cytologically Indeterminate Thyroid Nodules Improves Markedly by Using a Predictive Model. *Eur Thyroid J* (2019) 8:83–89. doi: 10.1159/000494720

114.	Ou D, Yao J, Jin J, Yan M, Shi K, Zheng Q, Yang C, Xu D. Ultrasonic identification and regression analysis of 294 thyroid follicular tumors. *J Cancer Res Ther* (2020) 16:1056–1062. doi: 10.4103/jcrt.JCRT_913_19

115.	Öz B, Doğan S, Emek E, Akyüz M, Akcan A, Sözüer E, Akyıldız H, Arslan E. Predictive Factors of Malignancy in Cytology of Indeterminate Follicular and Hürthle Cell Neoplasms of the Thyroid Gland. *International Surgery* (2018) 103:9–14. doi: 10.9738/INTSURG-D-15-00187.1

116.	Özdemi̇r A, Uyan M, Kalcan S, Çolakoğlu MK, Pergel A. Malignancy rates and risk factors in Bethesda category IV thyroid nodules: Is lobectomy enough in an endemic region? *J Exp Clin Med* (2022) 39:749–754. doi: 10.52142/omujecm.39.3.30

117.	Pak K, Shin S, Kim SJ, Kim K, Kim BS, Kim SJ, Jeon YK, Kim SS, Kim BH, Kim I-J. Risk of Malignancy in Thyroid Incidentaloma is Not Increased in Overweight or Obese Patients, but in Young Patients. *Nutr Cancer* (2017) 69:580–584. doi: 10.1080/01635581.2017.1299877

118.	Pappa T, Ahmadi S, Bikas A, Hwang S, Coleman A, Lobon I, Xiang P, Kim M, Marqusee E, Richman DM, et al. Thyroid Nodule Shape Independently Predicts Risk of Malignancy. *J Clin Endocrinol Metab* (2022) 107:1865–1870. doi: 10.1210/clinem/dgac246

119.	Peng X, Zhu X, Cheng F, Zhou B, Zhu X, Zhu L. Correlation between thyroid autoantibodies and the risk of thyroid papillary carcinoma. *Gland Surg* (2020) 9:950–955. doi: 10.21037/gs-20-445

120.	Peng B, Zhang S, Du F. Risk Factors and Prediction Models for Postoperative Pathologically Malignant TI-RADS 3 Thyroid Nodules. *Ear Nose Throat J* (2024)1455613241228078. doi: 10.1177/01455613241228078

121.	Petric R, Besic H, Besic N. Preoperative serum thyroglobulin concentration as a predictive factor of malignancy in small follicular and Hürthle cell neoplasms of the thyroid gland. *World J Surg Oncol* (2014) 12:282. doi: 10.1186/1477-7819-12-282

122.	Pikis G, Kandaraki E, Lamnisos D, Abbara S, Kyriakou K, Economides A, Economides PA. Prevalence of thyroid carcinoma in nodules with thy 3 cytology: the role of preoperative ultrasonography and strain elastography. *Thyroid Res* (2021) 14:7. doi: 10.1186/s13044-021-00098-x

123.	Pompili GG, Tresoldi S, Ravelli A, Primolevo A, Di Leo G, Carrafiello G. Use of the ultrasound-based total malignancy score in the management of thyroid nodules. *Ultrasonography* (2018) 37:315–322. doi: 10.14366/usg.17063

124.	Qureshi K, Rathore M, Waheed N-U-A, Lodhi S, Yasmin N, Gondal AJ, Choudhry N. Estimation of Serum Thyroid Stimulating Hormone and Anti-Thyroid Antibodies as Biomarkers of Malignancy in Thyroid Nodule Patients. *Pakistan Journal of Medical & Health Sciences* (2022) 16:177–177. doi: 10.53350/pjmhs20221611177

125.	Rago T, Scutari M, Latrofa F, Loiacono V, Piaggi P, Marchetti I, Romani R, Basolo F, Miccoli P, Tonacchera M, et al. The large majority of 1520 patients with indeterminate thyroid nodule at cytology have a favorable outcome, and a clinical risk score has a high negative predictive value for a more cumbersome cancer disease. *J Clin Endocrinol Metab* (2014) 99:3700–3707. doi: 10.1210/jc.2013-4401

126.	Ramundo V, Lamartina L, Falcone R, Ciotti L, Lomonaco C, Biffoni M, Giacomelli L, Maranghi M, Durante C, Grani G. Is thyroid nodule location associated with malignancy risk? *Ultrasonography* (2019) 38:231–235. doi: 10.14366/usg.18050

127.	Ren T, Jiang M, Wu J, Zhang F, Zhang C. Clinical value of grayscale ultrasound combined with real-time shear wave elastography nomogram in risk prediction of thyroid cancer. *BMC Med Imaging* (2023) 23:123. doi: 10.1186/s12880-023-01099-y

128.	Rianto BUD, Wibowo AS, Herdini C. The Difference in Thyroid Stimulating Hormone Levels between Differentiated Carcinoma and Benign Enlargement. *Int Arch Otorhinolaryngol* (2020) 24:e73–e79. doi: 10.1055/s-0039-1692406

129.	Rios A, Torregrosa B, Rodríguez JM, Rodríguez D, Cepero A, Abellán MD, Torregrosa NM, Hernández AM, Parrilla P. Ultrasonographic risk factors of malignancy in thyroid nodules. *Langenbecks Arch Surg* (2016) 401:839–849. doi: 10.1007/s00423-016-1451-y

130.	Rosenblum RC, Shtabsky A, Marmor S, Trejo L, Yaish I, Barnes S, Yehuda M, Stern N, Silman Z, Tordjman KM. Indeterminate nodules by the Bethesda system for reporting thyroid cytopathology in Israel: Frequency, and risk of malignancy after reclassification of follicular thyroid neoplasm with papillary-like features. *Eur J Surg Oncol* (2019) 45:1182–1187. doi: 10.1016/j.ejso.2019.03.015

131.	Ryu YJ, Jung YS, Yoon HC, Hwang MJ, Shin SH, Cho JS, Lee JS, Kim HK, Kang HC, Lim HS, et al. Atypia of undetermined significance on thyroid fine needle aspiration: surgical outcome and risk factors for malignancy. *Ann Surg Treat Res* (2014) 86:109–114. doi: 10.4174/astr.2014.86.3.109

132.	Şah Ünal FT, Gökçay Canpolat A, Elhan AH, Sevim S, Sak SD, Emral R, Demir Ö, Güllü S, Erdoğan MF, Çorapçıoğlu D, et al. Cancer rates and characteristics of thyroid nodules with macrocalcification. *Endocrine* (2024) 84:1021–1029. doi: 10.1007/s12020-023-03650-x

133.	Sahib A.M., Kadhim S.H., Shallan M.A. Preoperatively Undiagnosed Thyroid Cancer in Patients Undergoing Surgery for Benign Multinodular Goiter. *J Babol Univ Med Sci* (2023) 25:544–551.

134.	Sasson M, Kay-Rivest E, Shoukrun R, Florea A, Hier M, Forest V-I, Tamilia M, Payne RJ. The T4/T3 quotient as a risk factor for differentiated thyroid cancer: a case control study. *J Otolaryngol Head Neck Surg* (2017) 46:28. doi: 10.1186/s40463-017-0208-0

135.	Seo JW, Jang AL, Suh SH, Park HS, Kang MK, Hong JC. Atypia of undetermined significance on thyroid fine needle aspiration - risk factors for malignancy. *Clin Otolaryngol* (2017) 42:234–238. doi: 10.1111/coa.12700

136.	Shayganfar A, Hashemi P, Esfahani MM, Ghanei AM, Moghadam NA, Ebrahimian S. Prediction of thyroid nodule malignancy using thyroid imaging reporting and data system (TIRADS) and nodule size. *Clin Imaging* (2020) 60:222–227. doi: 10.1016/j.clinimag.2019.10.004

137.	Shin TJ, Rabbani CC, Murthy HD, Traylor K, Sim MW. Hürthle cell neoplasms of the thyroid: Pathologic outcomes and ultrasonographic analysis. *Laryngoscope Investig Otolaryngol* (2020) 5:1254–1259. doi: 10.1002/lio2.465

138.	Slijepcevic N, Zivaljevic V, Marinkovic J, Sipetic S, Diklic A, Paunovic I. Retrospective evaluation of the incidental finding of 403 papillary thyroid microcarcinomas in 2466 patients undergoing thyroid surgery for presumed benign thyroid disease. *BMC Cancer* (2015) 15:330. doi: 10.1186/s12885-015-1352-4

139.	Słowińska-Klencka D, Wysocka-Konieczna K, Klencki M, Popowicz B. Diagnostic Value of Six Thyroid Imaging Reporting and Data Systems (TIRADS) in Cytologically Equivocal Thyroid Nodules. *J Clin Med* (2020) 9:2281. doi: 10.3390/jcm9072281

140.	Sohn SY, Kim HJ, Jang HW, Kim SW, Chung JH. Lack of association between high serum thyroid-stimulating hormone level and risk of papillary thyroid microcarcinomas. *Head Neck* (2014) 36:43–46. doi: 10.1002/hed.23252

141.	Song Q, Tian X, Jiao Z, Yan L, Lan Y, Zhu Y, Luo Y. Value of Conventional Ultrasonography with Contrast-Enhanced Ultrasonography in the Differential Diagnosis of Partial Cystic Thyroid Nodules. *Ultrasound Med Biol* (2021) 47:2494–2501. doi: 10.1016/j.ultrasmedbio.2021.03.009

142.	Sun J, Liu J, Wu T-T, Gu Z-Y, Zhang X-W. Sensitivity to thyroid hormone indices are associated with papillary thyroid carcinoma in Chinese patients with thyroid nodules. *BMC Endocr Disord* (2023) 23:126. doi: 10.1186/s12902-023-01381-8

143.	Sutton W, Canner JK, Rooper LM, Prescott JD, Zeiger MA, Mathur A. Is Patient Age Associated with Risk of Malignancy in a ≥4cm Cytologically Benign Thyroid Nodule? *Am J Surg* (2021) 221:111–116. doi: 10.1016/j.amjsurg.2020.05.034

144.	Swan KZ, Nielsen VE, Godballe C, Thrane JF, Mortensen MR, Schytte S, Pedersen HB, Christiansen P, Bonnema SJ. Is serum TSH a biomarker of thyroid carcinoma in patients residing in a mildly iodine-deficient area? *Endocrine* (2018) 61:308–316. doi: 10.1007/s12020-018-1637-x

145.	Talmor G, Badash I, Zhou S, Kim YJ, Kokot NC, Hsueh W, Chambers T. Association of patient characteristics, ultrasound features, and molecular testing with malignancy risk in Bethesda III-V thyroid nodules. *Laryngoscope Investig Otolaryngol* (2022) 7:1243–1250. doi: 10.1002/lio2.847

146.	Tam AA, Özdemir D, Fakı S, Bilginer MC, Ersoy R, Çakır B. ABO Blood Groups, Rh Factor, and Thyroid Cancer Risk: To “B” or Not to “B.” *Endocr Res* (2020) 45:137–146. doi: 10.1080/07435800.2019.1695261

147.	Tang Q, Liu X, Jiang Q, Zhu L, Zhang J, Wu P-Y, Jiang Y, Zhou J. Unenhanced magnetic resonance imaging of papillary thyroid carcinoma with emphasis on diffusion kurtosis imaging. *Quant Imaging Med Surg* (2023) 13:2697–2707. doi: 10.21037/qims-22-172

148.	Teklewold B, Mekuria B, Chane W. Rate and Risk of Follicular Carcinoma in Patients with a Cytologic Diagnosis of Follicular Neoplasia: Experience from Tertiary Hospital in Ethiopia. *OAS* (2022) Volume 15:31–37. doi: 10.2147/OAS.S355808

149.	Trimboli P, Deandrea M, Mormile A, Ceriani L, Garino F, Limone PP, Giovanella L. American Thyroid Association ultrasound system for the initial assessment of thyroid nodules: Use in stratifying the risk of malignancy of indeterminate lesions. *Head Neck* (2018) 40:722–727. doi: 10.1002/hed.25038

150.	Turkyilmaz S, Ulusahin M, Celebi B, Cekic AB, Mungan S, Kucuktulu U, Tasdelen A, Guner A, Cinel A. Thyroid nodules classified as atypia or follicular lesions of undetermined significance deserve further research: Analysis of 305 surgically confirmed nodules. *Cytopathology* (2017) 28:391–399. doi: 10.1111/cyt.12438

151.	Tutuncu Y, Berker D, Isik S, Akbaba G, Ozuguz U, Kucukler FK, Göcmen E, Yalcın Y, Aydin Y, Guler S. The frequency of malignancy and the relationship between malignancy and ultrasonographic features of thyroid nodules with indeterminate cytology. *Endocrine* (2014) 45:37–45. doi: 10.1007/s12020-013-9922-1

152.	Vasileiadis I, Boutzios G, Charitoudis G, Koukoulioti E, Karatzas T. Thyroglobulin antibodies could be a potential predictive marker for papillary thyroid carcinoma. *Ann Surg Oncol* (2014) 21:2725–2732. doi: 10.1245/s10434-014-3593-x

153.	Wang L, Li H, Yang Z, Guo Z, Zhang Q. Preoperative Serum Thyrotropin to Thyroglobulin Ratio Is Effective for Thyroid Nodule Evaluation in Euthyroid Patients. *Otolaryngol Head Neck Surg* (2015) 153:15–19. doi: 10.1177/0194599815579877

154.	Wang H, Wei R, Liu W, Chen Y, Song B. Diagnostic efficacy of multiple MRI parameters in differentiating benign vs. malignant thyroid nodules. *BMC Med Imaging* (2018) 18:50. doi: 10.1186/s12880-018-0294-0

155.	Wang Z, Zhu X, Yu X, Guan H, Zhao L, Zhang Y, Li Y, Sang L, Han Y, Li Y, et al. The combination of ATA classification and FNA results can improve the diagnostic efficiency of malignant thyroid nodules. *Endocr Connect* (2020) 9:903–911. doi: 10.1530/EC-20-0303

156.	Wang Y, Dong T, Nie F, Wang G, Liu T, Niu Q. Contrast-Enhanced Ultrasound in the Differential Diagnosis and Risk Stratification of ACR TI-RADS Category 4 and 5 Thyroid Nodules With Non-Hypovascular. *Front Oncol* (2021) 11:662273. doi: 10.3389/fonc.2021.662273

157.	Wang G, Nie F, Wang Y, Wang P, Wang L, Fan X, Ma Z. Value of Echogenic Foci in Diagnosing Papillary Thyroid Carcinoma and Predicting Aggressive Biological Behavior. *J Ultrasound Med* (2022) 41:1237–1245. doi: 10.1002/jum.15815

158.	Witczak J, Taylor P, Chai J, Amphlett B, Soukias J-M, Das G, Tennant BP, Geen J, Okosieme OE. Predicting malignancy in thyroid nodules: feasibility of a predictive model integrating clinical, biochemical, and ultrasound characteristics. *Thyroid Res* (2016) 9:4. doi: 10.1186/s13044-016-0033-y

159.	Wong SL, Grodski S, Yeung MJ, Serpell JW. Anti-thyroid antibodies as a predictor of thyroid cancer. *ANZ J Surg* (2015) 85:849–853. doi: 10.1111/ans.12453

160.	Wong M.W., Hadi I.A., Musa K.I. Revisiting types and risk factors for thyroid malignancy in a state endemic of iodine deficiency goiter. *World J Endocr Surg* (2017) 9:51–54. doi: 10.5005/jp-journals-10002-1210

161.	Wu X, Lun Y, Jiang H, Gang Q, Xin S, Duan Z, Zhang J. Coexistence of thyroglobulin antibodies and thyroid peroxidase antibodies correlates with elevated thyroid-stimulating hormone level and advanced tumor stage of papillary thyroid cancer. *Endocrine* (2014) 46:554–560. doi: 10.1007/s12020-013-0121-x

162.	Wu S-J, Tan L, Ruan J-L, Qiu Y, Hao S-Y, Yang H-Y, Luo B-M. ACR TI-RADS classification combined with number of nodules, halo features optimizes diagnosis and prediction of follicular thyroid cancer. *Clin Hemorheol Microcirc* (2022) 82:323–334. doi: 10.3233/CH-221507

163.	Xi X, Wang Y, Gao L, Jiang Y, Liang Z, Ren X, Gao Q, Lai X, Yang X, Zhu S, et al. Establishment of an Ultrasound Malignancy Risk Stratification Model for Thyroid Nodules Larger Than 4 cm. *Front Oncol* (2021) 11:592927. doi: 10.3389/fonc.2021.592927

164.	Xin Y, Liu F, Shi Y, Yan X, Liu L, Zhu J. A Scoring System for Assessing the Risk of Malignant Partially Cystic Thyroid Nodules Based on Ultrasound Features. *Front Oncol* (2021) 11:731779. doi: 10.3389/fonc.2021.731779

165.	Xu S-Y, Zhan W-W, Wang W-H. Evaluation of Thyroid Nodules by a Scoring and Categorizing Method Based on Sonographic Features. *J Ultrasound Med* (2015) 34:2179–2185. doi: 10.7863/ultra.14.11041

166.	Xu D-H, Su C, Miao Z-Y, Jin M-S, Chen G, Meng X-Y, Wang G-M. Predictive role of high-density lipoprotein and body mass index in differentiated thyroid carcinoma. *Int J Clin Exp Med* (2018) 11:2172–2182.

167.	Xu S, Ni X, Zhou W, Zhan W, Zhang H. Development and validation of a novel diagnostic tool for predicting the malignancy probability of thyroid nodules: A retrospective study based on clinical, B-mode, color doppler and elastographic ultrasonographic characteristics. *Front Endocrinol (Lausanne)* (2022) 13:966572. doi: 10.3389/fendo.2022.966572

168.	Xu N, Liu H, Wang Y, Xue Y. Relationship between insulin resistance and thyroid cancer in Chinese euthyroid subjects without conditions affecting insulin resistance. *BMC Endocr Disord* (2022) 22:58. doi: 10.1186/s12902-022-00943-6

169.	Yan H, Pang P, Wang F, Tian W, Luo Y, Huang W, Yang G, Jin N, Zang L, Du J, et al. Dynamic profile of differentiated thyroid cancer in male and female patients with thyroidectomy during 2000–2013 in China: a retrospective study. *Sci Rep* (2017) 7:15832. doi: 10.1038/s41598-017-14963-z

170.	Yang T, Huang Y, Jing X, Gai X, Li W. CT-detected solitary thyroid calcification: an important imaging feature for papillary carcinoma. *Onco Targets Ther* (2016) 9:6273–6279. doi: 10.2147/OTT.S113369

171.	Yazdani-Charati J, Akha O, Khosravi F. Factors Affecting Thyroid Cancer in Patients with Thyroid Nodules Using Logistic Regression in Interval Censored Data. *Int J Cancer Manag* (2018) 11:e9111. doi: 10.5812/ijcm.9111

172.	Yazgan A, Balci S, Dincer N, Ersoy PE, Tuzun D, Ersoy R, Irkkan C, Cakir B, Guler G. Thyroid FNAC containing hürthle cells and hürthle-like cells: A study of 128 cases. *J Cytol* (2016) 33:214–219. doi: 10.4103/0970-9371.190447

173.	Yazici P, Mihmanli M, Bozkurt E, Ozturk FY, Uludag M. Which is the best predictor of thyroid cancer: thyrotropin, thyroglobulin or their ratio? *Hormones (Athens)* (2016) 15:256–263. doi: 10.14310/horm.2002.1677

174.	Yeon EK, Sohn Y-M, Seo M, Kim E-J, Eun Y-G, Park WS, Yun SJ. Diagnostic Performance of a Combination of Shear Wave Elastography and B-Mode Ultrasonography in Differentiating Benign From Malignant Thyroid Nodules. *Clin Exp Otorhinolaryngol* (2020) 13:186–193. doi: 10.21053/ceo.2019.01235

175.	Yi D, Fan L, Zhu J, Yao J, Peng C, Xu D. The diagnostic value of a nomogram based on multimodal ultrasonography for thyroid-nodule differentiation: A multicenter study. *Front Oncol* (2022) 12:970758. doi: 10.3389/fonc.2022.970758

176.	Yoo MR, Gweon HM, Park AY, Cho KE, Kim J-A, Youk JH, Son EJ. Repeat Diagnoses of Bethesda Category III Thyroid Nodules: What To Do Next? *PLoS One* (2015) 10:e0130138. doi: 10.1371/journal.pone.0130138

177.	Yoon JH, Lee HS, Kim E-K, Moon HJ, Kwak JY. A nomogram for predicting malignancy in thyroid nodules diagnosed as atypia of undetermined significance/follicular lesions of undetermined significance on fine needle aspiration. *Surgery* (2014) 155:1006–1013. doi: 10.1016/j.surg.2013.12.035

178.	Yu Z, Yu Y, Wan Y, Fan J, Meng H, Li S, Wang Y, Wang T, Ling R. Iodine intake level and incidence of thyroid disease in adults in Shaanxi province: a cross-sectional study. *Ann Transl Med* (2021) 9:1567. doi: 10.21037/atm-21-4928

179.	Yu B, Li Y, Yu X, Ai Y, Jin J, Zhang J, Zhang Y, Zhu H, Xie C, Shen M, et al. Differentiate Thyroid Follicular Adenoma from Carcinoma with Combined Ultrasound Radiomics Features and Clinical Ultrasound Features. *J Digit Imaging* (2022) 35:1362–1372. doi: 10.1007/s10278-022-00639-2

180.	Zeng R, Shou T, Yang K-X, Shen T, Zhang J-P, Zuo R-X, Zheng Y-Q, Yan X-M. Papillary thyroid carcinoma risk factors in the Yunnan plateau of southwestern China. *Ther Clin Risk Manag* (2016) 12:1065–1074. doi: 10.2147/TCRM.S105023

181.	Zhang Y-F, Liu C, Xu H-X, Xu J-M, Zhang J, Guo L-H, Zheng S-G, Liu L-N, Xu X-H. Acoustic radiation force impulse imaging: a new tool for the diagnosis of papillary thyroid microcarcinoma. *Biomed Res Int* (2014) 2014:416969. doi: 10.1155/2014/416969

182.	Zhang H, Shi Q, Gu J, Jiang L, Bai M, Liu L, Wu Y, Du L. Combined value of Virtual Touch tissue quantification and conventional sonographic features for differentiating benign and malignant thyroid nodules smaller than 10 mm. *J Ultrasound Med* (2014) 33:257–264. doi: 10.7863/ultra.33.2.257

183.	Zhang J, Chen Z, Anil G. Ultrasound-guided thyroid nodule biopsy: outcomes and correlation with imaging features. *Clin Imaging* (2015) 39:200–206. doi: 10.1016/j.clinimag.2014.10.019

184.	Zhang X, Zhang X, Chang Z, Wu C, Guo H. Correlation analyses of thyroid-stimulating hormone and thyroid autoantibodies with diﬀerentiated thyroid cancer. *J BUON* (2018) 23:1467–1471.

185.	Zhang F, Oluwo O, Castillo FB, Gangula P, Castillo M, Farag F, Zakaria S, Zahedi T. THYROID NODULE LOCATION ON ULTRASONOGRAPHY AS A PREDICTOR OF MALIGNANCY. *Endocr Pract* (2019) 25:131–137. doi: 10.4158/EP-2018-0361

186.	Zhang X, Ze Y, Sang J, Shi X, Bi Y, Shen S, Zhang X, Zhu D. Risk factors and diagnostic prediction models for papillary thyroid carcinoma. *Front Endocrinol (Lausanne)* (2022) 13:938008. doi: 10.3389/fendo.2022.938008

187.	Zhang F, Mei F, Chen W, Zhang Y. Role of Ultrasound and Ultrasound-Based Prediction Model in Differentiating Follicular Thyroid Carcinoma From Follicular Thyroid Adenoma. *J Ultrasound Med* (2024) 43:1389–1399. doi: 10.1002/jum.16461

188.	Zhao R-N, Zhang B, Yang X, Jiang Y-X, Lai X-J, Zhang X-Y. Logistic Regression Analysis of Contrast-Enhanced Ultrasound and Conventional Ultrasound Characteristics of Sub-centimeter Thyroid Nodules. *Ultrasound Med Biol* (2015) 41:3102–3108. doi: 10.1016/j.ultrasmedbio.2015.04.026

189.	Zhao H, Li H, Huang T. High Urinary Iodine, Thyroid Autoantibodies, and Thyroid-Stimulating Hormone for Papillary Thyroid Cancer Risk. *Biol Trace Elem Res* (2018) 184:317–324. doi: 10.1007/s12011-017-1209-6

190.	Zhao S, Jia X, Fan X, Zhao L, Pang P, Wang Y, Luo Y, Wang F, Yang G, Wang X, et al. Association of obesity with the clinicopathological features of thyroid cancer in a large, operative population: A retrospective case-control study. *Medicine (Baltimore)* (2019) 98:e18213. doi: 10.1097/MD.0000000000018213

191.	Zhao R-N, Zhang B, Jiang Y-X, Yang X, Lai X-J, Zhu S-L, Zhang X-Y. Ultrasonographic Multimodality Diagnostic Model of Thyroid Nodules. *Ultrason Imaging* (2019) 41:63–77. doi: 10.1177/0161734618815070

192.	Zhao Z, Ren T, Zhao Y, Xu W, Xie R, Lin J, Li H, Zheng L, Zhang C, Huo H, et al. Salivary biomarkers-assisted ultrasound-based differentiation of malignant and benign thyroid nodules. *Gland Surg* (2022) 11:196–206. doi: 10.21037/gs-21-864

193.	Zivic R, Diklic A, Sipetic Grujicic S, Paunovic I, Vekic B, Perunovic R, Radovanovic D, Zivaljevic V. Analysis of malignancy predictors for oxyphile thyroid tumors. *J BUON* (2016) 21:1496–1500.

194.	Zoric G.V., Nikolic-Djurovic M.M., Paunovic I.R., Diklic A.D., Bukumiric Z.M., Slijepcevic N.A., Tausanovic K.M., Odalovic B.A., Jovanovic M.D., Zivaljevic V.R. Analysis of malignancy predictors for follicular thyroid tumors. *Vojnosanit Pregl* (2020) 77:282–288. doi: 10.2298/VSP180314079Z
